# Supplementary material for: Synthesis and Cytotoxic Activity of 1,2,4-Triazolo-Linked Bis-Indolyl Conjugates as Dual Inhibitors of Tankyrase and PI3K
Source: Molecules. 2022 Nov 7;27(21):7642. doi: 10.3390/molecules27217642 (PMC9657870; doi:10.3390/molecules27217642)
Supplement: Supplementary file 1 [file molecules-27-07642-s001.zip › molecules-2013576-supplementary.pdf]

List of the Contents

Dose response curve……………………………………………………………..……..**Figure** S1

Docking images…………………………………………………...……...…….**Figure** S2

^{1}H, ^{13}C NMR and Mass copies of all products ………………..……………………**Figure** S3

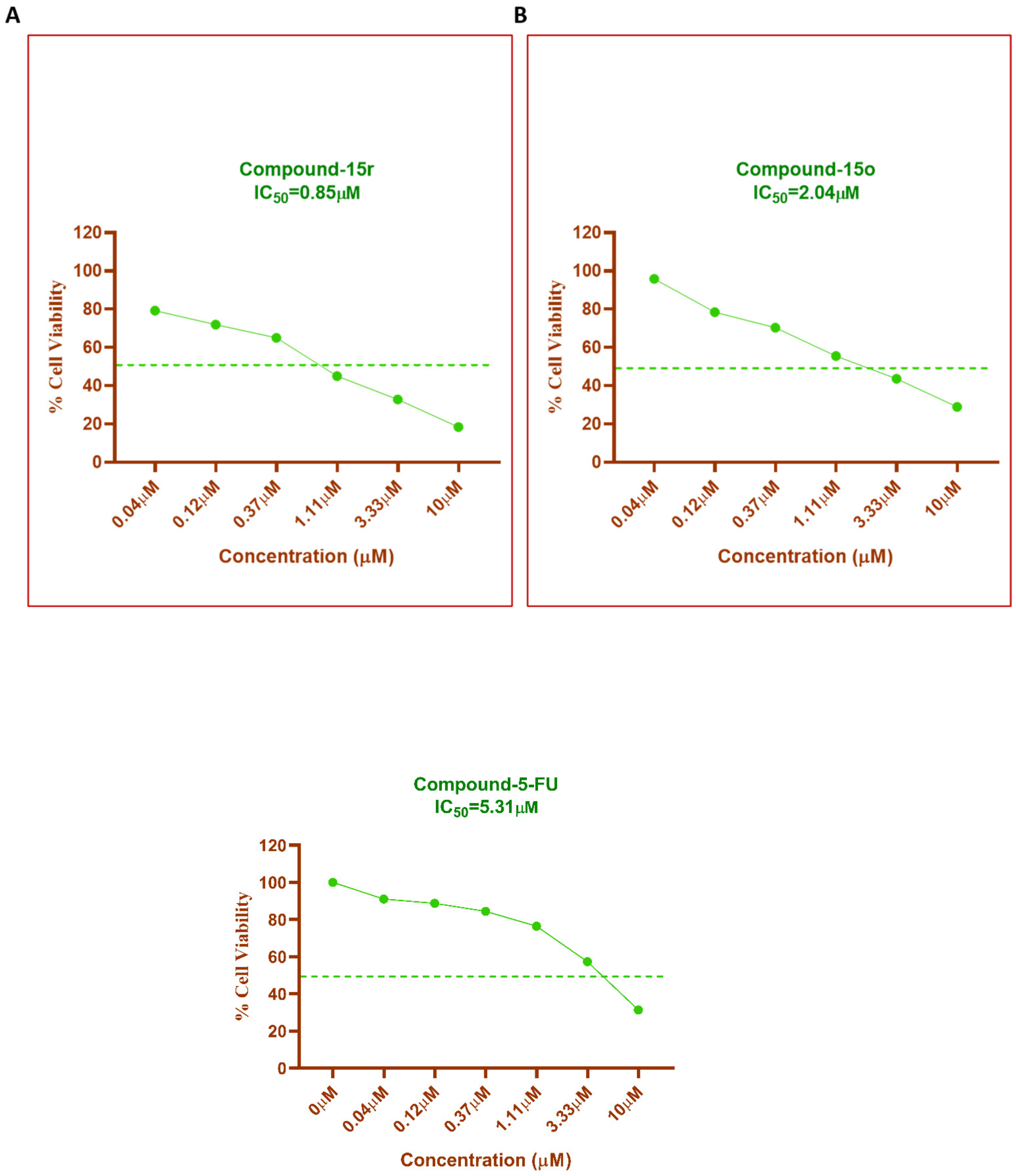

Figure S1: Dose Response Curves for 15r, 15o and Standard compounds against HT-29 colon cancer cell lines.

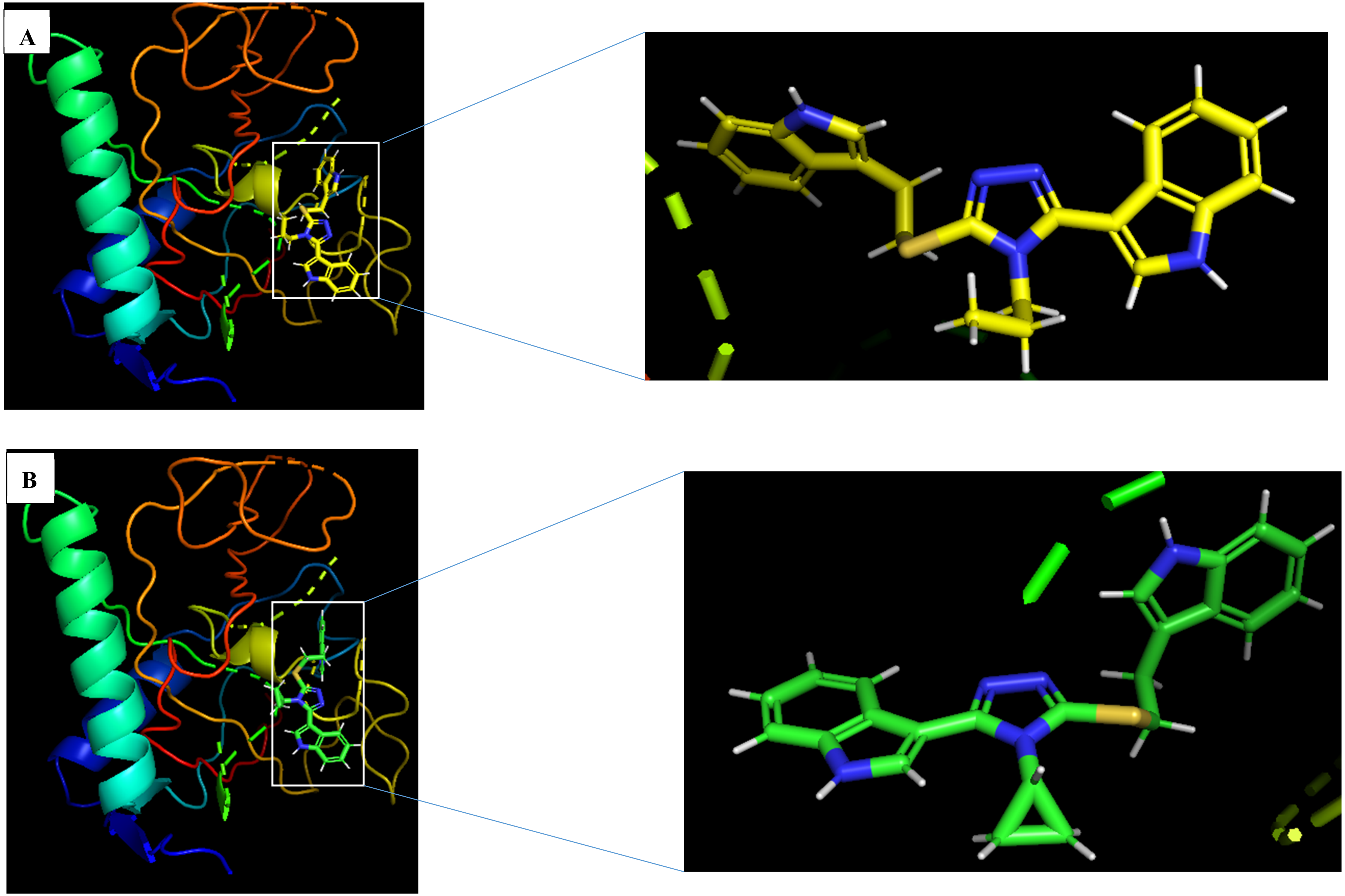
A
B

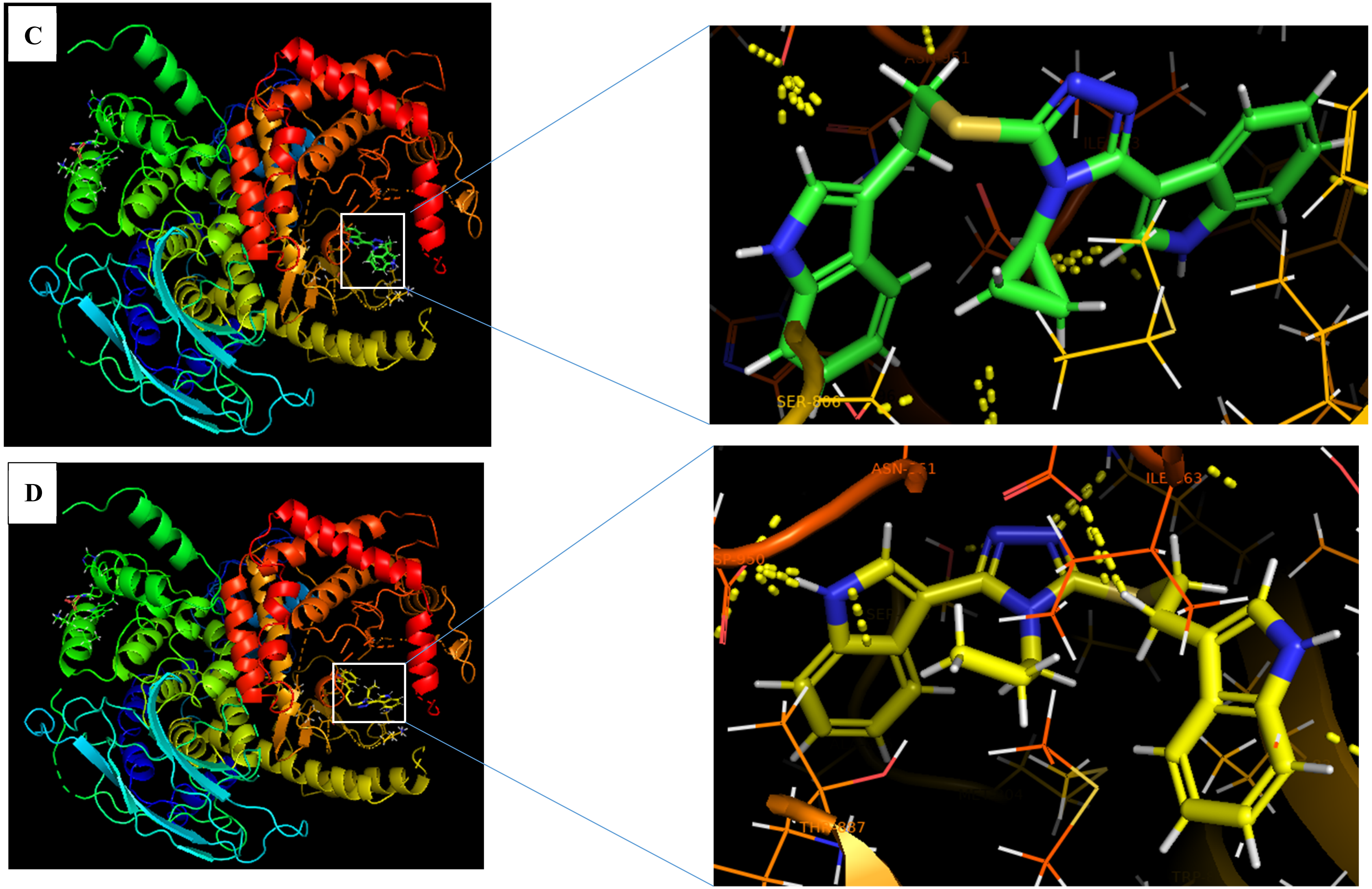
C
D

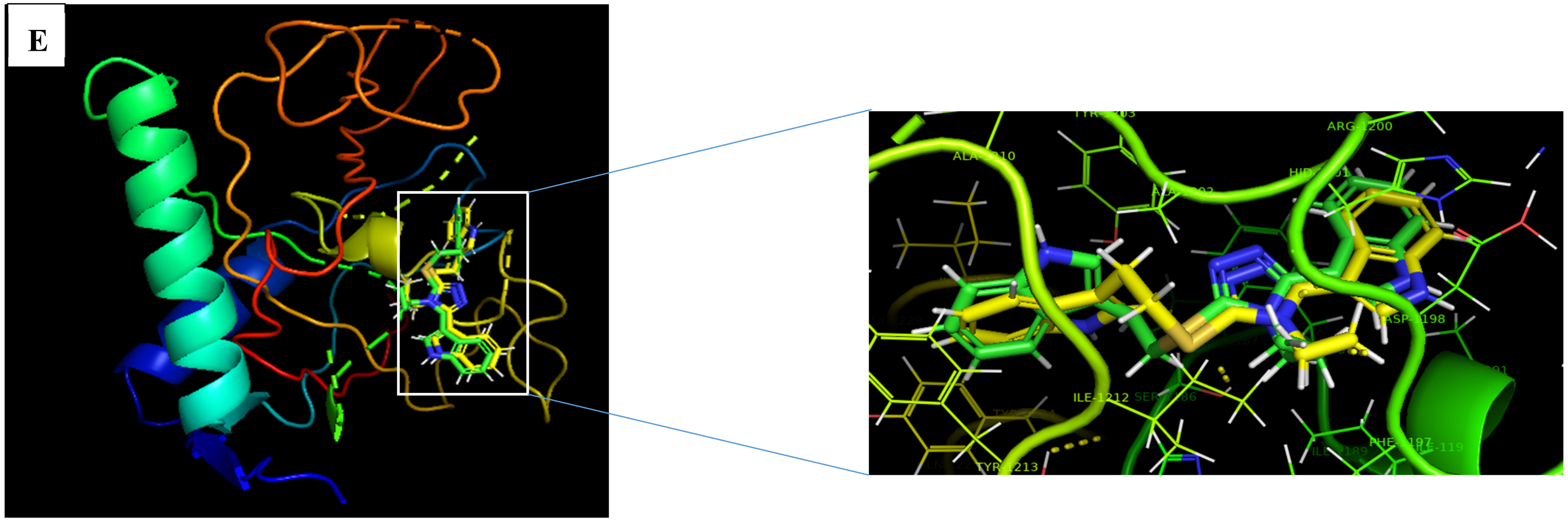
E
ARG-1200
ILE-1212
TYR-1213

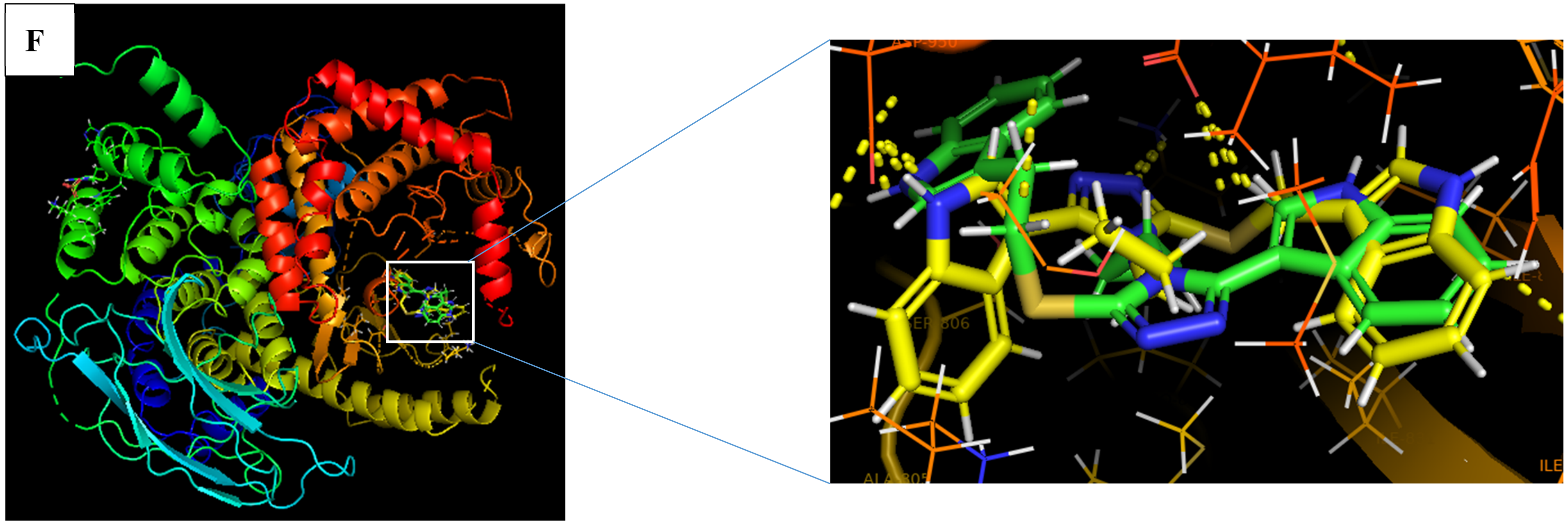
F

G

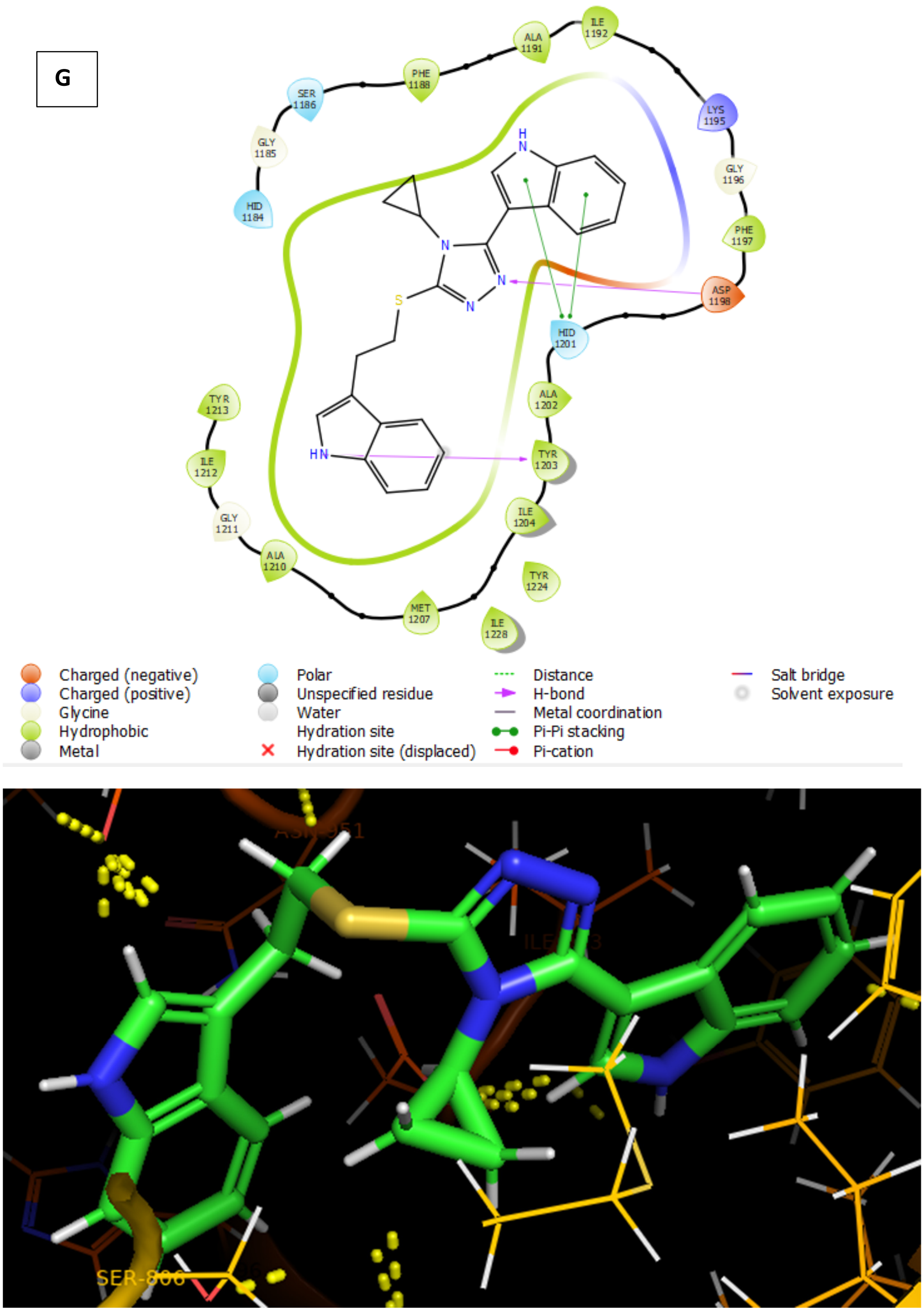
ILE 1192
ALA 1191
PHE 1188
SER 1186
GLY 1185
HID 1184
LYS 1195
GLY 1196
PHE 1197
ASP 1198
HID 1201
ALA 1202
TYR 1203
ILE 1204
TYR 1224
ILE 1228
MET 1207
ALA 1210
GLY 1211
ILE 1212
TYR 1213
H N
N
N
N
S
HN
Charged (negative)
Charged (positive)
Glycine
Hydrophobic
Metal
Polar
Unspecified residue
Water
Hydration site
Hydration site (displaced)
Distance
H-bond
Metal coordination
Pi-Pi stacking
Pi-cation
Salt bridge
Solvent exposure
SER-806

H

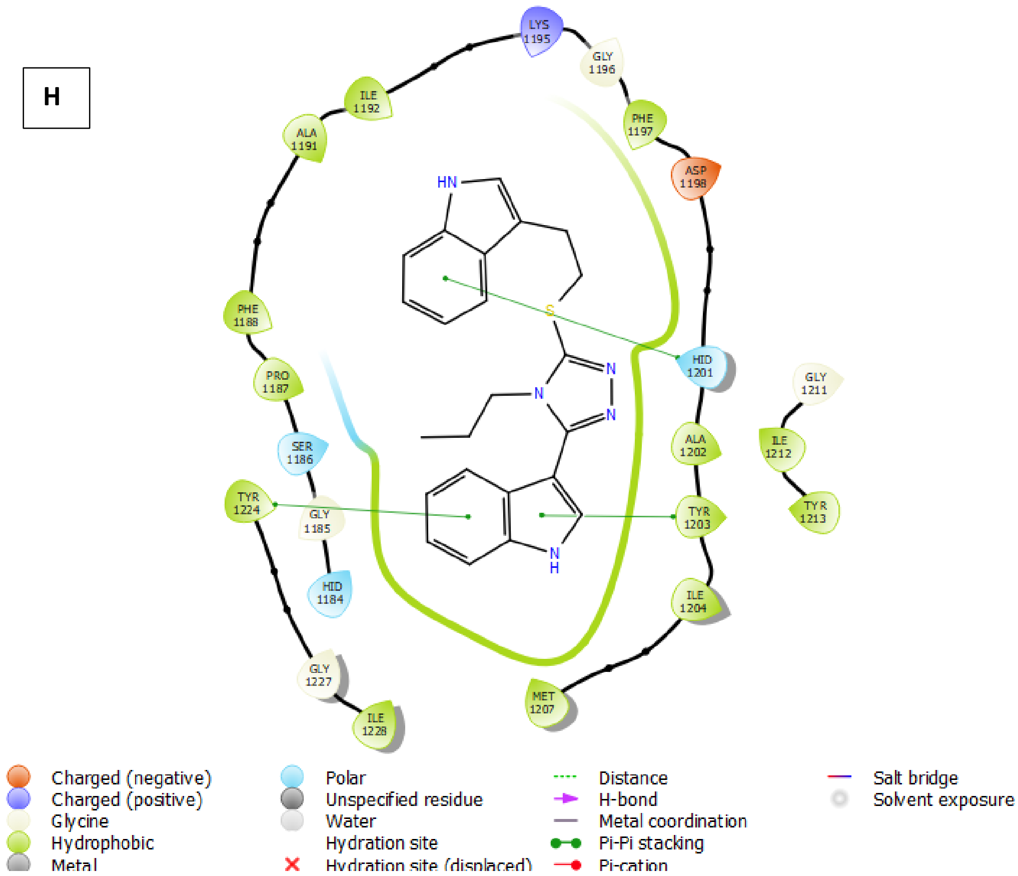
LYS 1195
GLY 1196
PHE 1197
ASP 1198
ILE 1192
ALA 1191
PHE 1188
PRO 1187
SER 1186
TYR 1224
GLY 1185
HID 1184
GLY 1227
ILE 1228
MET 1207
ILE 1204
TYR 1203
ALA 1202
HID 1201
GLY 1211
ILE 1212
TYR 1213
HN
S
N
N
N
N
H
Charged (negative)
Charged (positive)
Glycine
Hydrophobic
Metal
Polar
Unspecified residue
Water
Hydration site
Hydration site (displaced)
Distance
H-bond
Metal coordination
Pi-Pi stacking
Pi-cation
Salt bridge
Solvent exposure

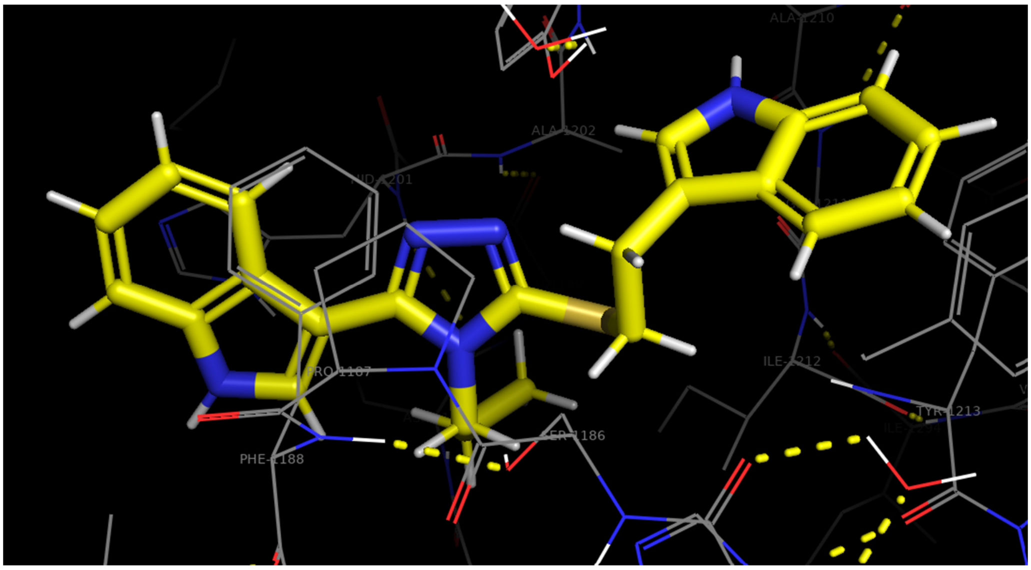
ALA 1210
ALA 1202
ILE 1212
TYR 1213
PRO 1187
PHE 1188

I

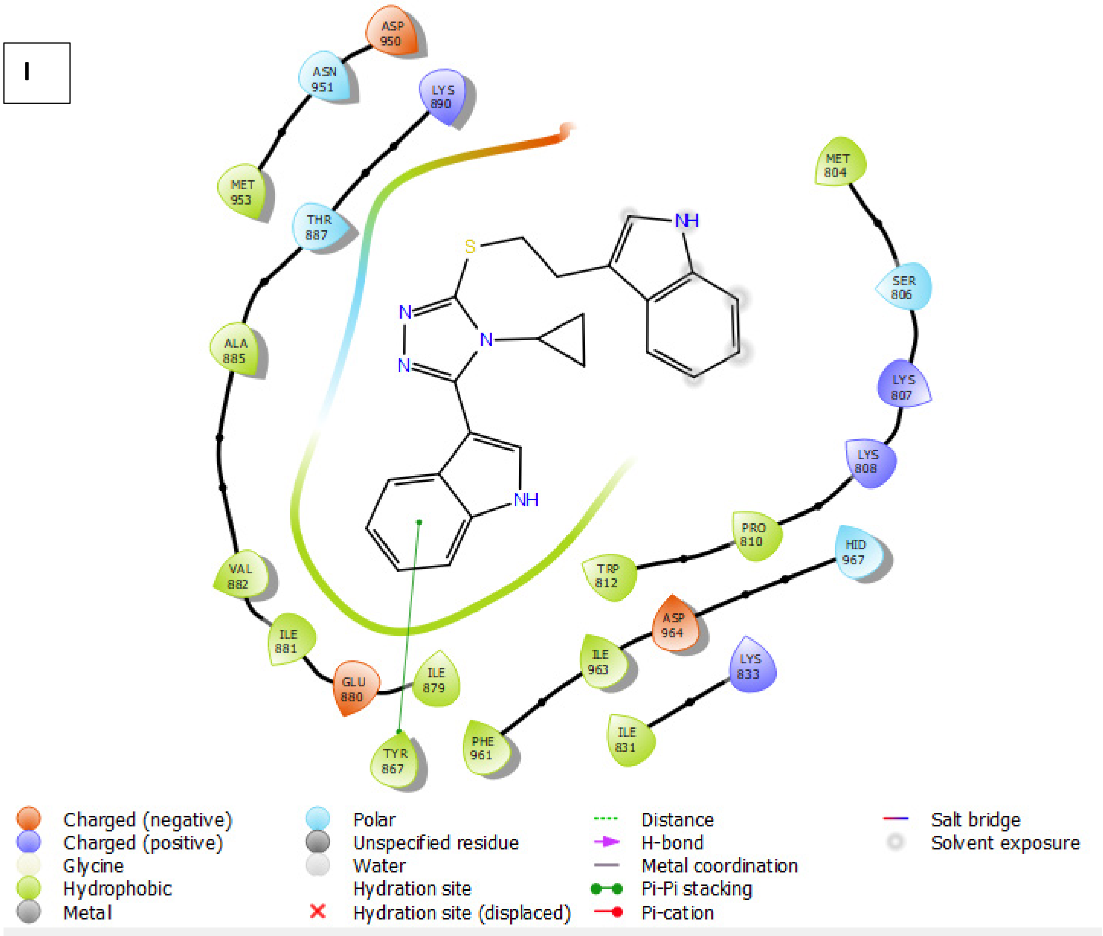
ASP 950
ASN 951
LYS 890
MET 953
THR 887
ALA 885
VAL 882
ILE 881
GLU 880
ILE 879
TYR 867
PHE 961
ILE 963
ASP 964
TRP 812
PRO 810
LYS 808
LYS 807
SER 806
MET 804
HID 967
LYS 833
ILE 831
S
N
N
N
NH
NH
Charged (negative)
Charged (positive)
Glycine
Hydrophobic
Metal
Polar
Unspecified residue
Water
Hydration site
Hydration site (displaced)
Distance
H-bond
Metal coordination
Pi-Pi stacking
Pi-cation
Salt bridge
Solvent exposure

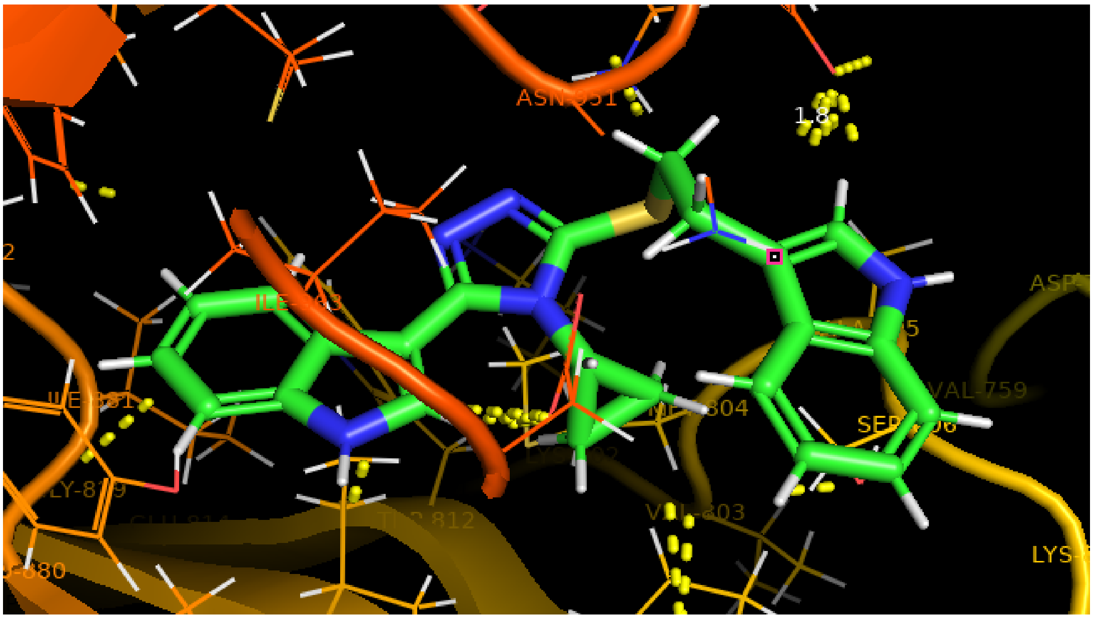
ASN-951
1.8
VAL-759

J

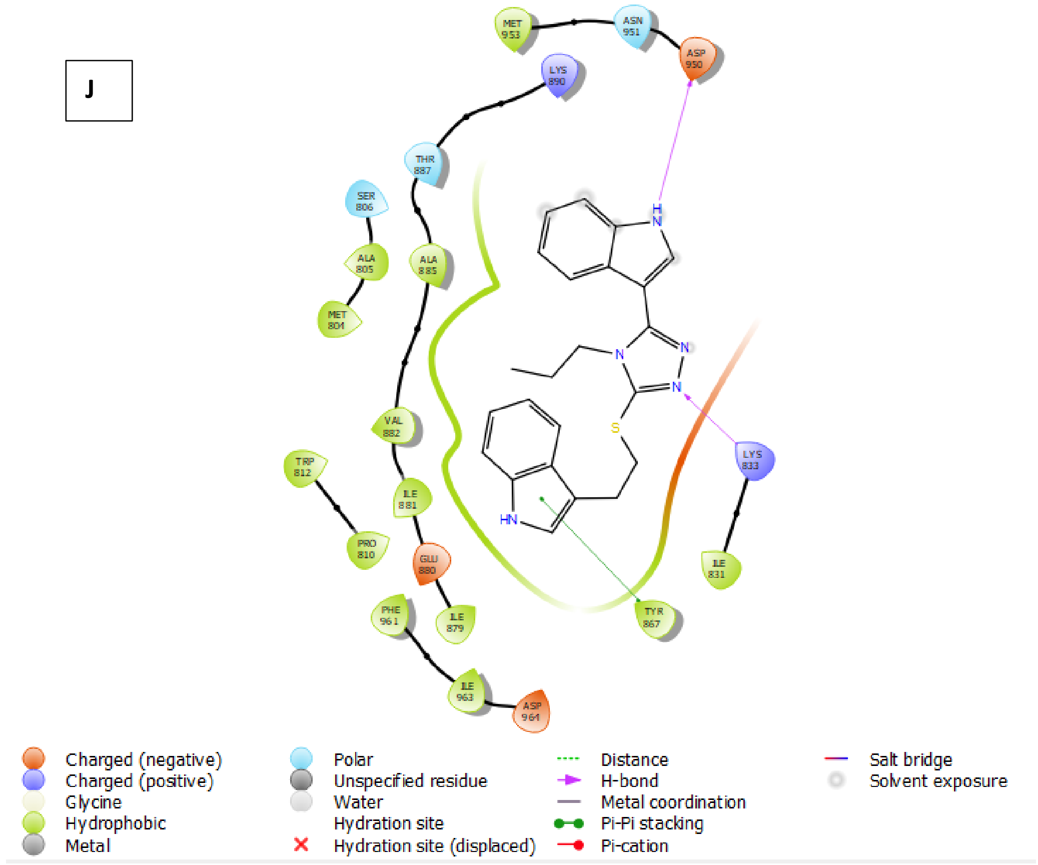
MET 953
ASN 951
ASP 950
LYS 890
THR 887
SER 806
ALA 805
ALA 885
MET 804
VAL 882
TRP 812
ILE 881
PRO 810
GLU 880
PHE 961
ILE 879
TYR 867
LYS 833
ILE 831
ILE 963
ASP 964
Charged (negative)
Charged (positive)
Glycine
Hydrophobic
Metal
Polar
Unspecified residue
Water
Hydration site
Hydration site (displaced)
Distance
H-bond
Metal coordination
Pi-Pi stacking
Pi-cation
Salt bridge
Solvent exposure

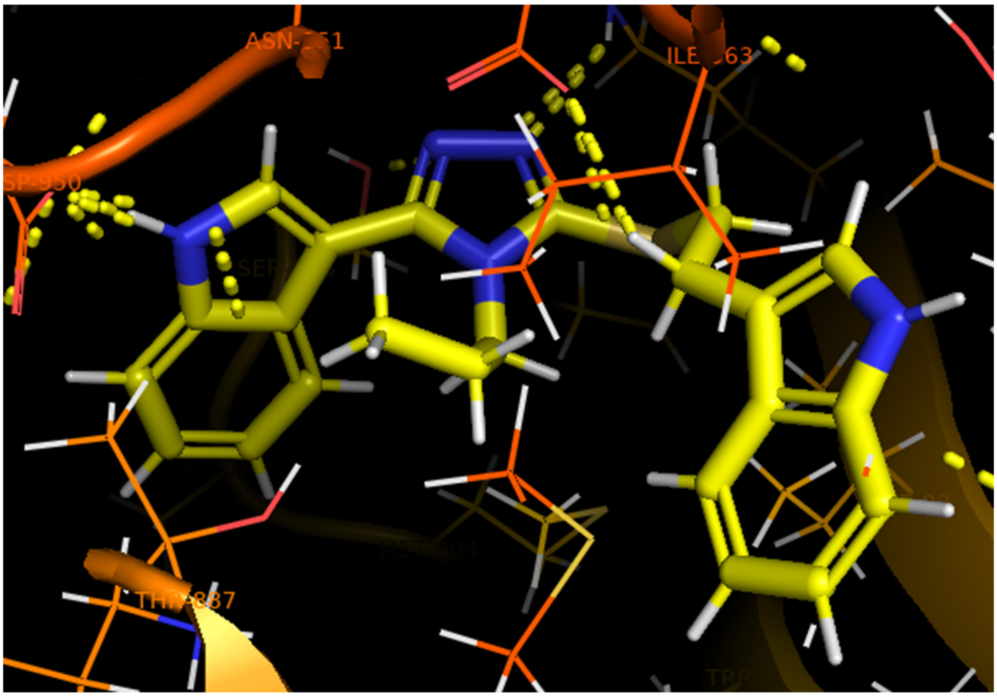

Figure S2. **(A)** Docking pose of 15o in PDB ID 4OA7 is shown in a 3D structure; **(B)** Docking pose of 15r in PDB ID 4OA7 is shown in a 3D structure. **(C)** Docking pose of 15r in PDB ID 3L54 is shown in a 3D structure; **(D)** Docking pose of 15o in PDB ID 3L54 is shown in a 3D structure. **(E)** Docking pose of 15o & 15r in PDB ID 4OA7 is shown in a 3D structure; **(F)** Docking pose of 15o & 15r in PDB ID 3L54 is shown in a 3D structure. **(G)** Docking pose of **15r** in PDB ID 4OA7 is shown in a 2D structure where indole NH bind with TYR 1203 *via* H-bonding is represented by pink arrow line similarly triazole N was formed with ASP 1198. On the other hand, the indole was shown a Pi-Pi staking with HID 1201 represented by a green line; **(H)** Docking pose of **15o** in PDB ID 4OA7 are shown in a 2D structure where both indoles was shown a Pi-Pi staking with HID 1201, TYR 1203, and TYR 1224 represented by green line; **(I)** Docking pose of **15r** in PDB ID 3L54 is shown in a 2D structure where indole was shown Pi-Pi staking with TYR 867 represented by green line; **(J)** Docking pose of **15o** in PDB ID 3L54 is shown in a 2D structure where indole NH and triazole N bind with LYS 833 and ASP 950 respectively via H-bonding is represented by pink arrow line. On the other hand, indole was shown Pi-Pi staking with TYR 867 represented by the green line.

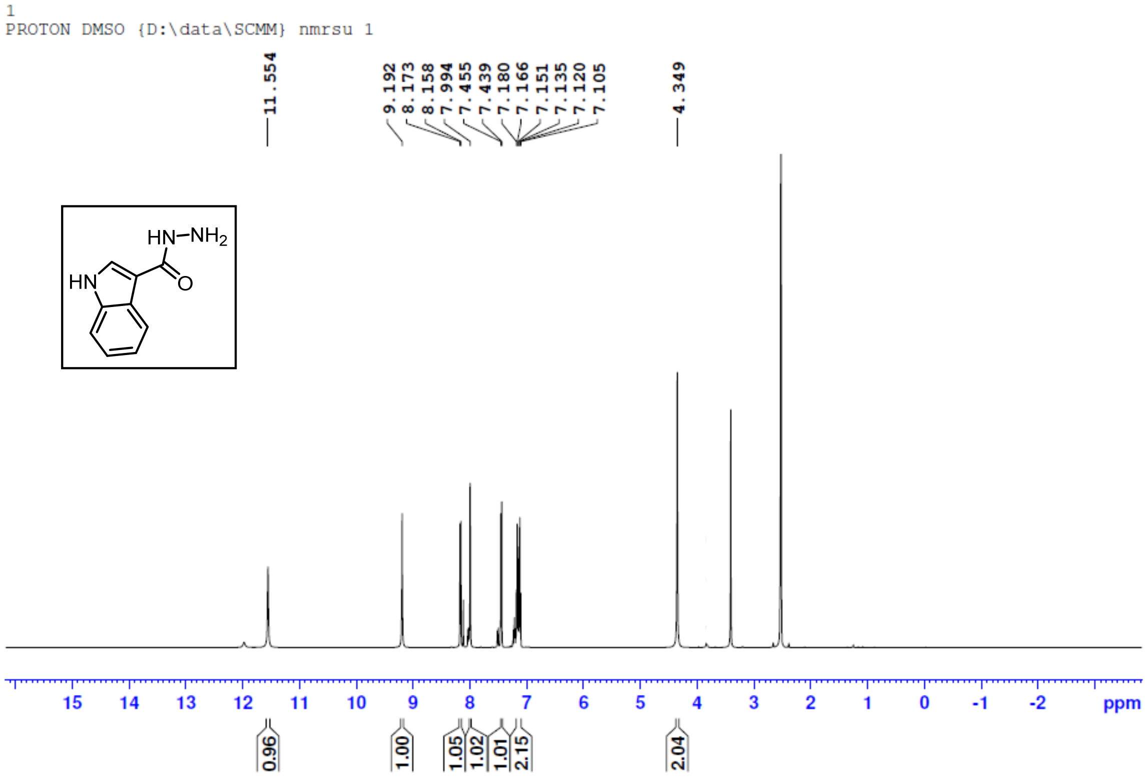
1
PROTON DMSO {D:\data\SCMM} nmrsu 1
11.554
9.192
8.173
8.158
7.994
7.455
7.439
7.180
7.166
7.151
7.135
7.120
7.105
4.349
HN–NH2
HN
O
15 14 13 12 11 10 9 8 7 6 5 4 3 2 1 0 -1 -2 ppm
0.96
1.00
1.05
1.02
1.01
2.15
2.04

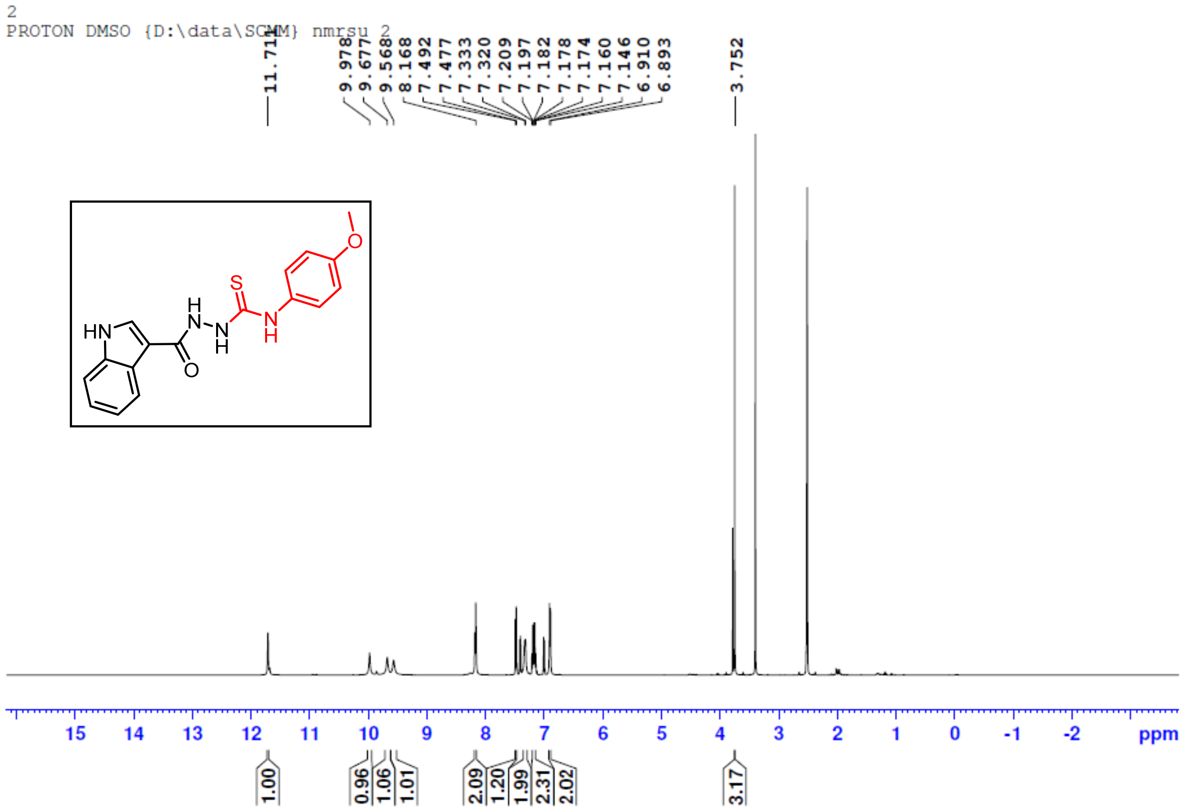
2
PROTON DMSO {D:\data\SCMM} nmrsu 2
11.711
9.978
9.677
9.568
8.168
7.492
7.477
7.333
7.320
7.209
7.197
7.182
7.178
7.174
7.160
7.146
6.910
6.893
3.752
HN
H
N
N
H
S
N
H
O
O
15 14 13 12 11 10 9 8 7 6 5 4 3 2 1 0 -1 -2 ppm
1.00
0.96
1.06
1.01
2.09
1.20
1.99
2.31
2.02
3.17

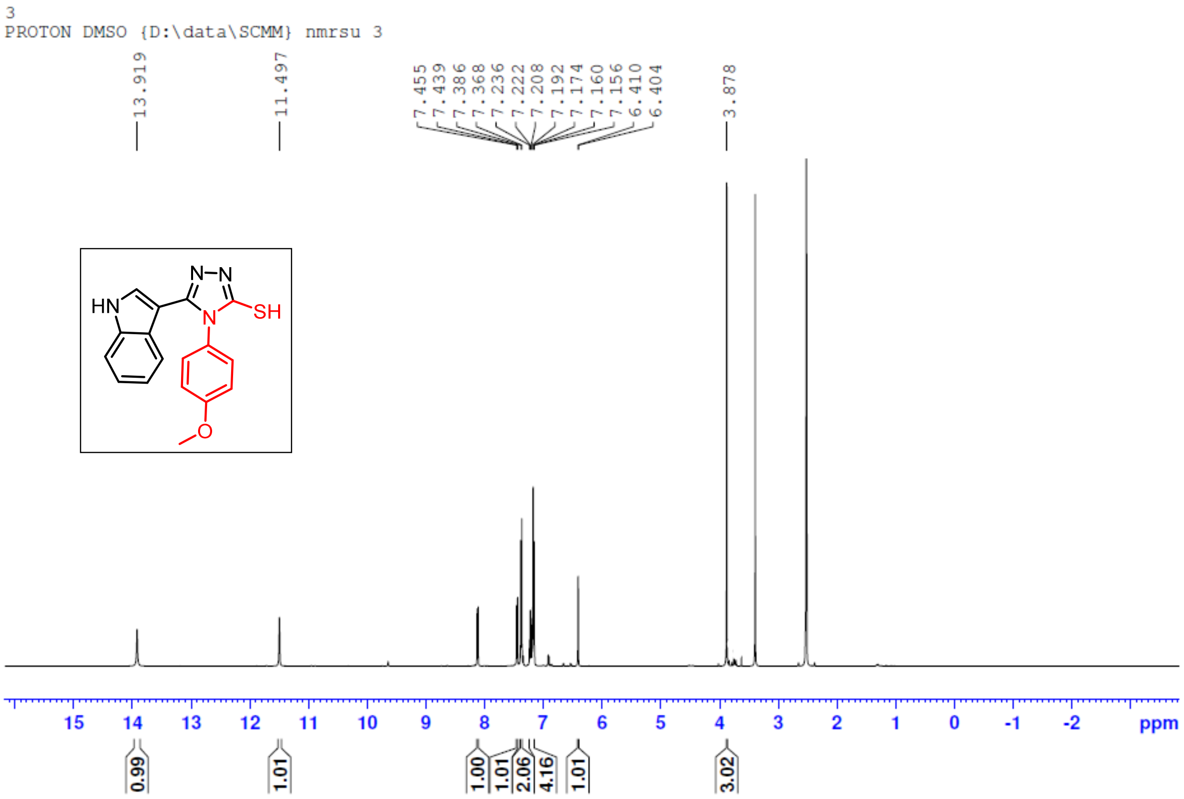
3
PROTON DMSO {D:\data\SCMM} nmrsu 3
13.919
11.497
7.455
7.439
7.386
7.368
7.236
7.222
7.208
7.192
7.174
7.160
7.156
6.410
6.404
3.878
HN
N–N
N
SH
O
15 14 13 12 11 10 9 8 7 6 5 4 3 2 1 0 -1 -2 ppm
0.99
1.01
1.00
1.01
2.06
4.16
1.01
3.02

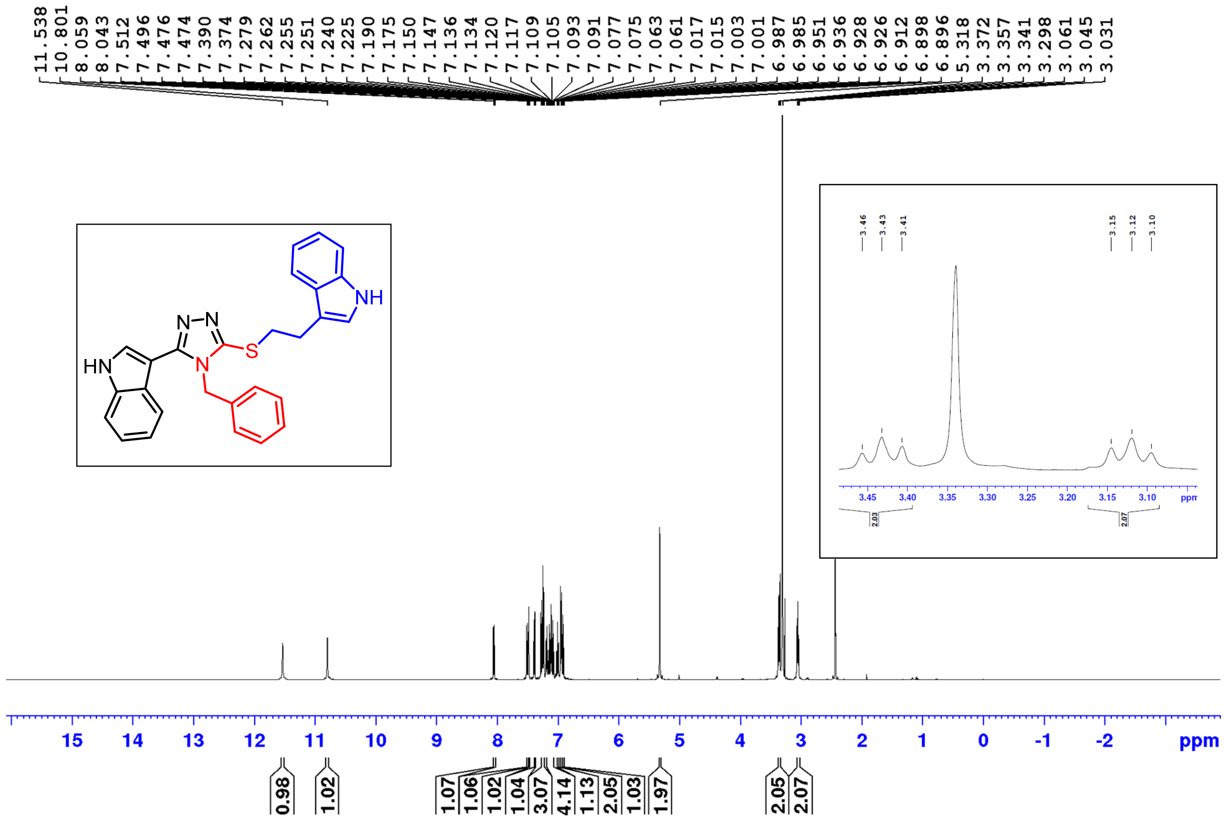
11.538
10.801
8.059
8.043
7.512
7.496
7.476
7.474
7.390
7.374
7.279
7.262
7.255
7.251
7.240
7.225
7.190
7.175
7.150
7.147
7.136
7.134
7.120
7.117
7.109
7.105
7.093
7.091
7.077
7.075
7.063
7.061
7.017
7.015
7.003
7.001
6.987
6.985
6.951
6.936
6.928
6.926
6.912
6.898
6.896
5.318
3.372
3.357
3.341
3.298
3.061
3.045
3.031
N–N
S
N
HN
NH
3.46
3.43
3.41
3.15
3.12
3.10
ppm
2.03
2.07
0.98
1.02
1.07
1.06
1.02
1.04
3.07
4.14
1.13
2.05
1.03
1.97
2.05
2.07
15 14 13 12 11 10 9 8 7 6 5 4 3 2 1 0 -1 -2 ppm

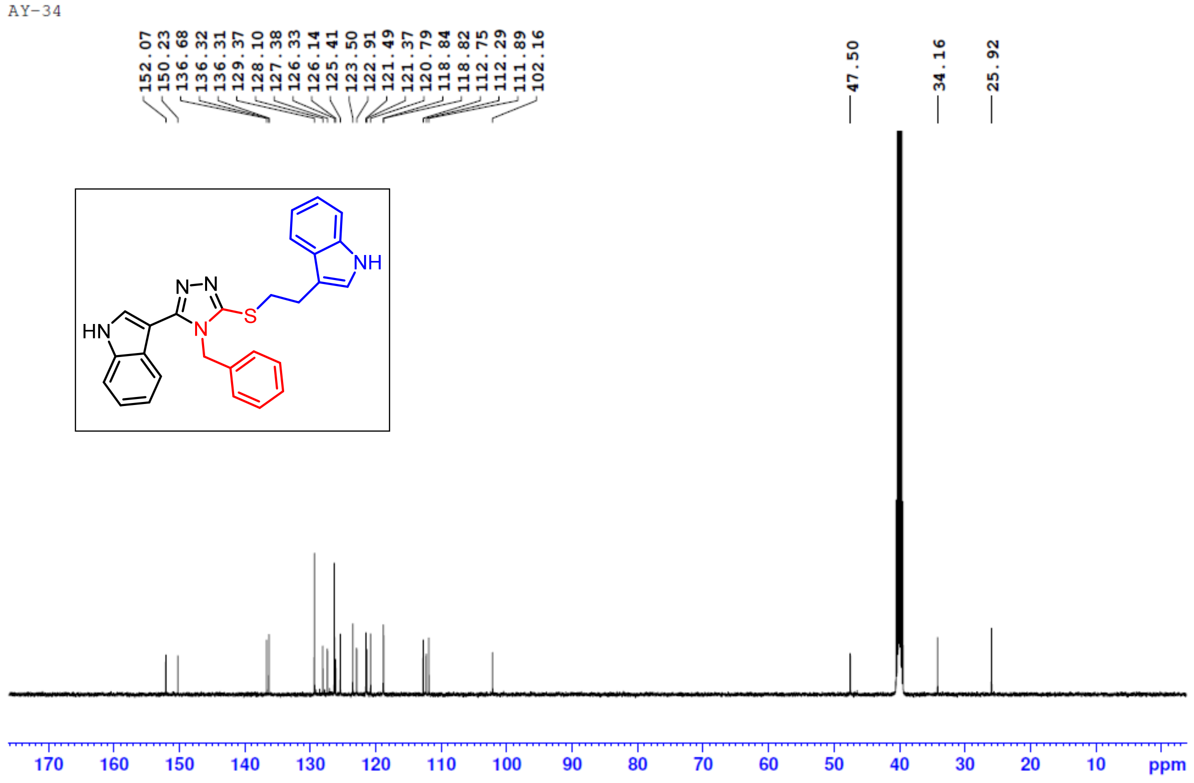
AY-34
152.07
150.23
136.68
136.32
136.31
129.37
128.10
127.38
126.33
126.14
125.41
123.50
122.91
121.49
121.37
120.79
118.84
118.82
112.75
112.29
111.89
102.16
47.50
34.16
25.92
N–N
S
N
HN
NH
170 160 150 140 130 120 110 100 90 80 70 60 50 40 30 20 10 ppm

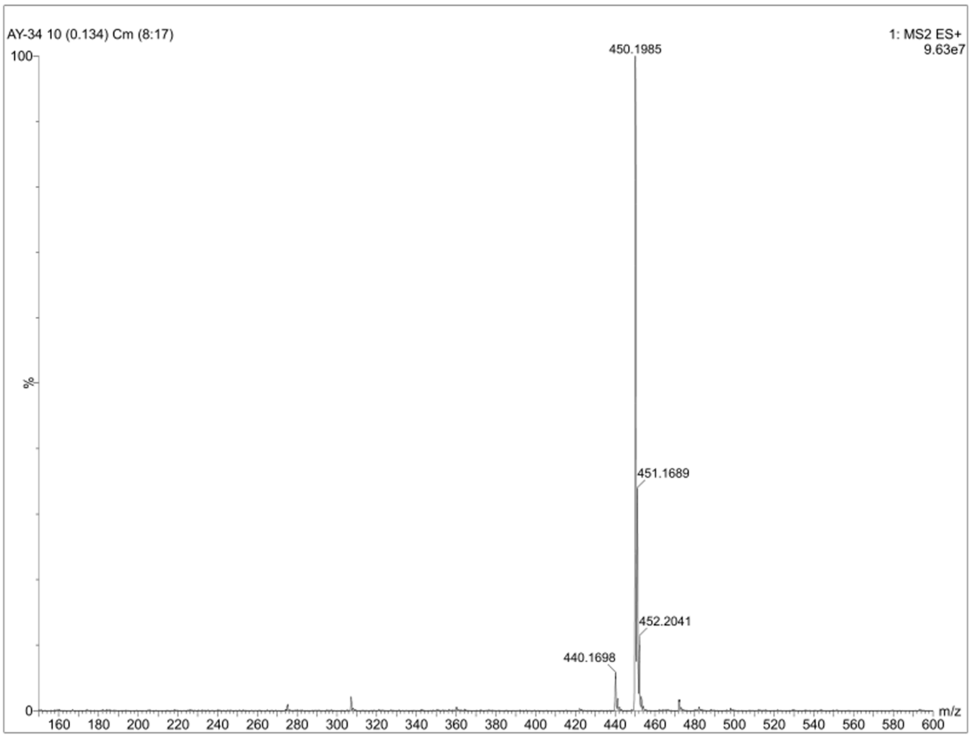
AY-34 10 (0.134) Cm (8:17)
1: MS2 ES+
9.63e7
450.1985
451.1689
452.2041
440.1698
100
%
0
m/z
160 180 200 220 240 260 280 300 320 340 360 380 400 420 440 460 480 500 520 540 560 580 600

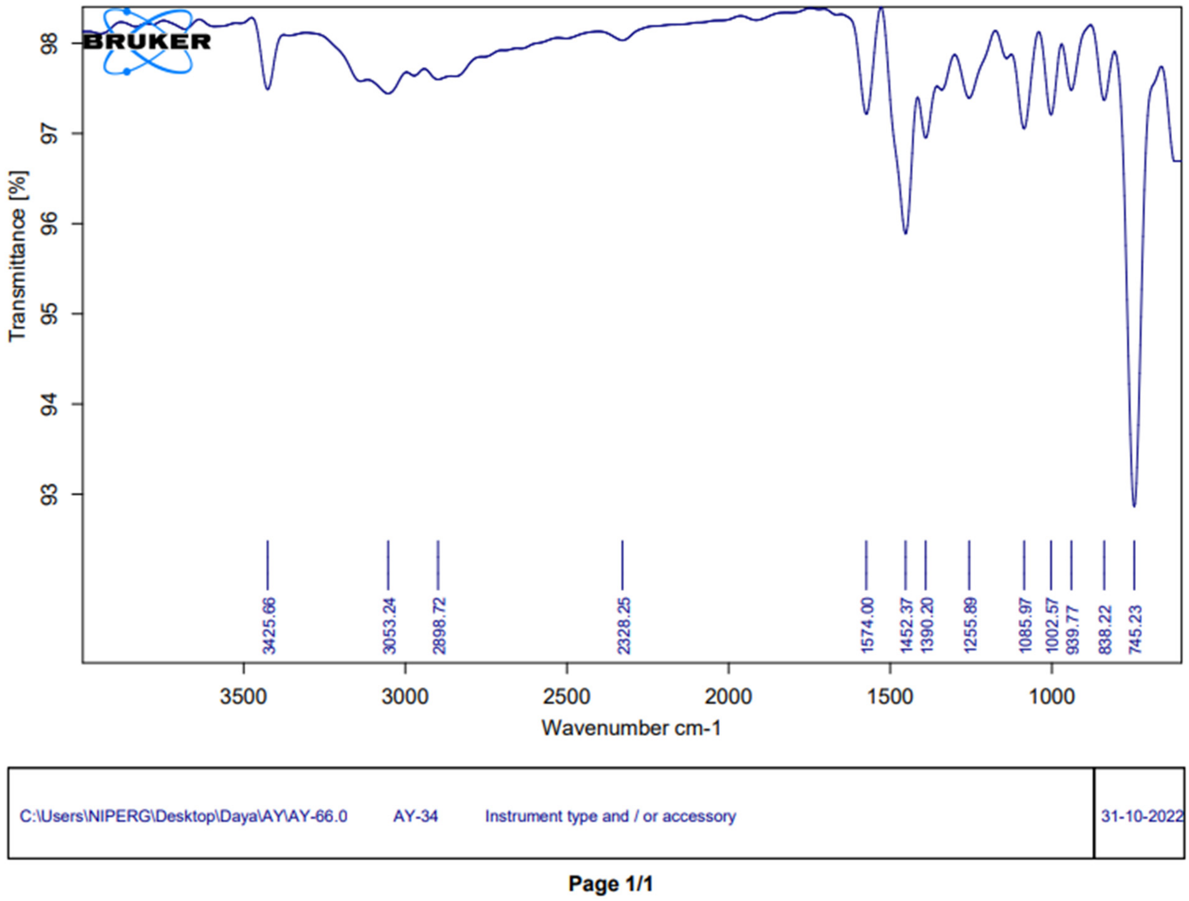
BRUKER
Transmittance [%]
98
97
96
95
94
93
3425.66
3053.24
2898.72
2328.25
1574.00
1452.37
1390.20
1255.89
1085.97
1002.57
939.77
838.22
745.23
3500 3000 2500 2000 1500 1000
Wavenumber cm-1
C:\Users\NIPERG\Desktop\Daya\AY\AY-66.0
AY-34
Instrument type and / or accessory
31-10-2022
Page 1/1

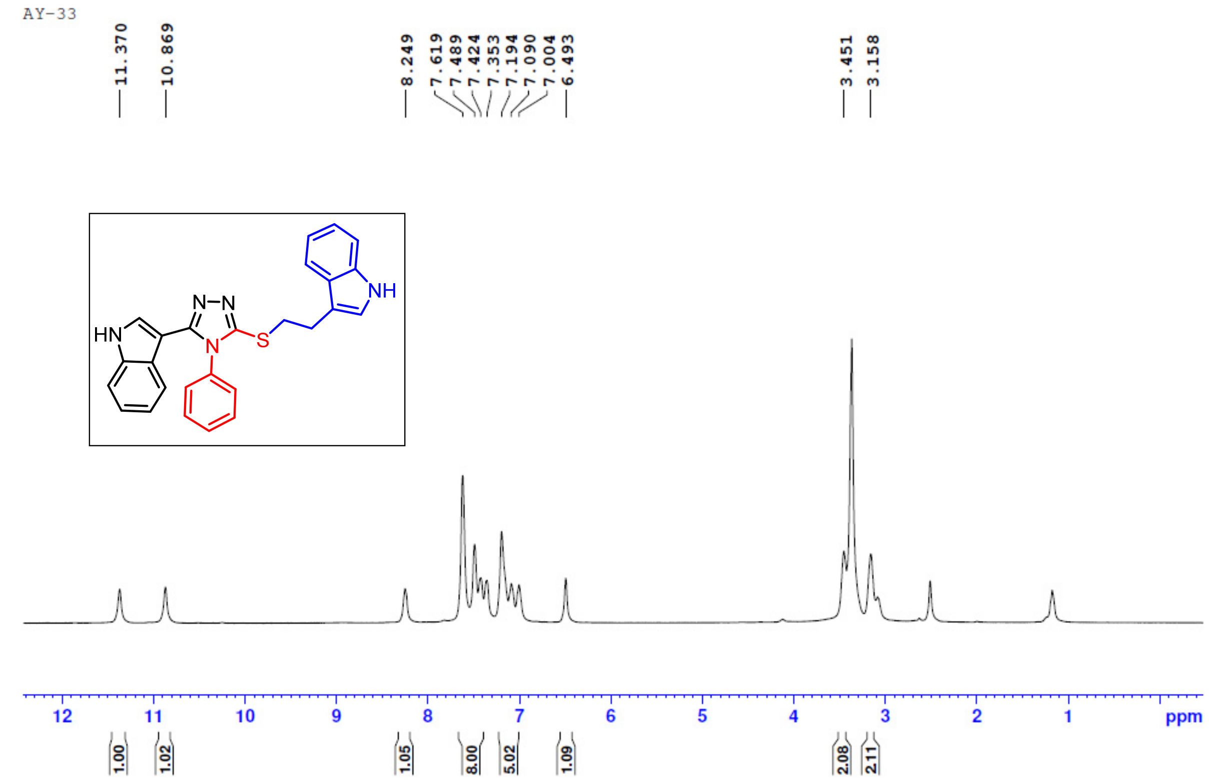
AY-33
11.370
10.869
8.249
7.619
7.489
7.424
7.353
7.194
7.090
7.004
6.493
3.451
3.158
HN
N–N
N
S
NH
12 11 10 9 8 7 6 5 4 3 2 1 ppm
1.00
1.02
1.05
8.00
5.02
1.09
2.08
2.11

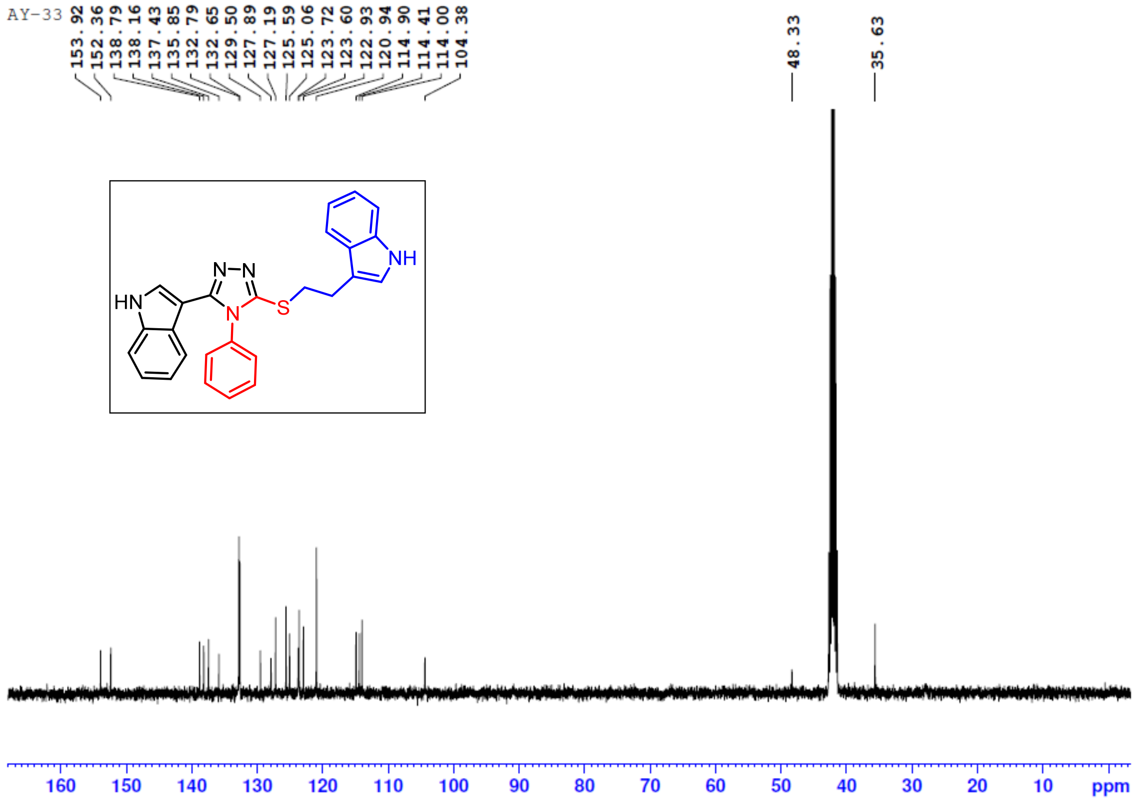
AY-33
153.92
152.36
138.79
138.16
137.43
135.85
132.79
132.65
129.50
127.89
127.19
125.59
125.06
123.72
123.60
122.93
120.94
114.90
114.41
114.00
104.38
48.33
35.63
HN
N–N
N
S
NH
160 150 140 130 120 110 100 90 80 70 60 50 40 30 20 10 ppm

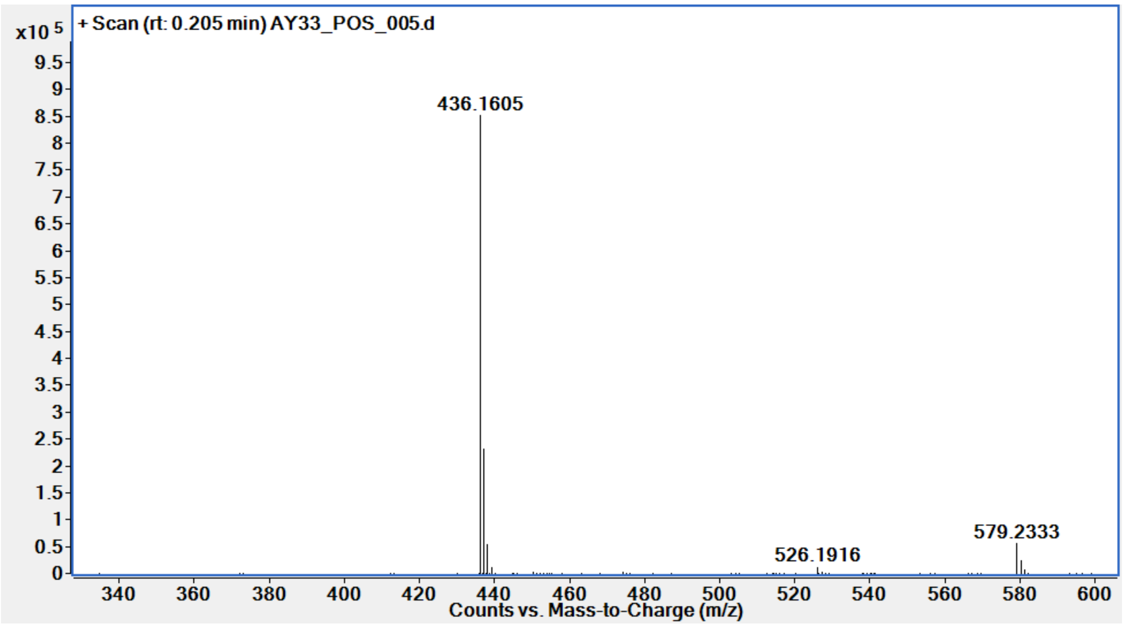
+ Scan (rt: 0.205 min) AY33_POS_005.d
x10 5
436.1605
526.1916
579.2333
Counts vs. Mass-to-Charge (m/z)

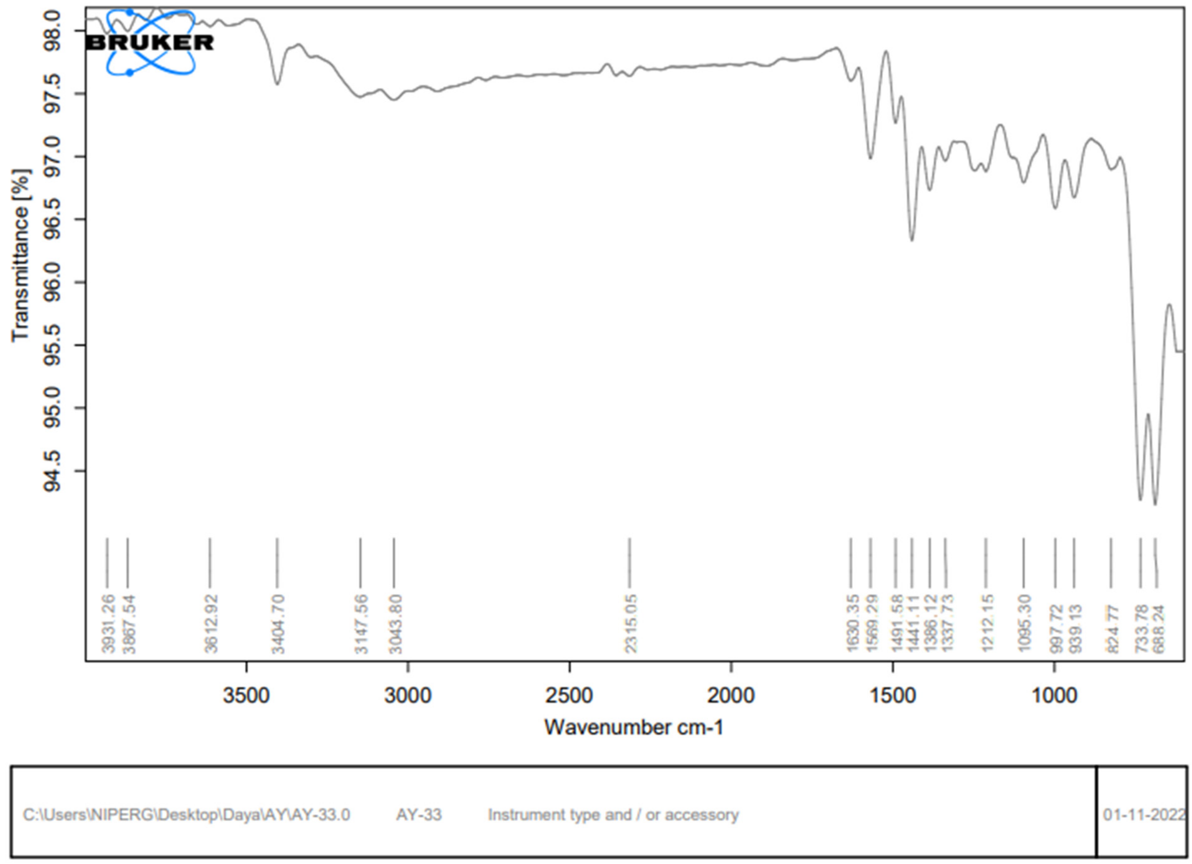
BRUKER
Transmittance [%]
Wavenumber cm-1
3931.26
3867.54
3612.92
3404.70
3147.56
3043.80
2315.05
1630.35
1569.29
1491.58
1441.11
1386.12
1337.73
1212.15
1095.30
997.72
939.13
824.77
733.78
688.24
C:\Users\NIPERG\Desktop\Daya\AY\AY-33.0
AY-33
Instrument type and / or accessory
01-11-2022

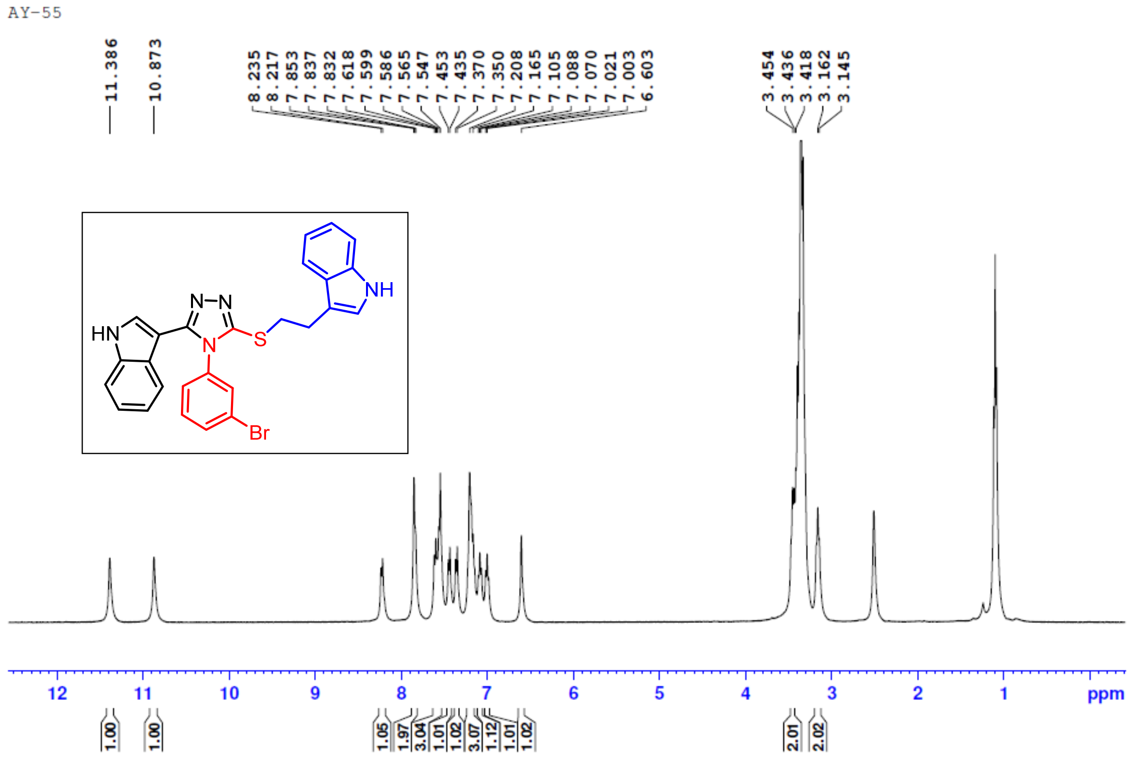
AY-55
11.386
10.873
8.235
8.217
7.853
7.837
7.832
7.618
7.599
7.586
7.565
7.547
7.453
7.435
7.370
7.350
7.208
7.165
7.105
7.088
7.070
7.021
7.003
6.603
3.454
3.436
3.418
3.162
3.145
N–N
HN
N
S
NH
Br
12 11 10 9 8 7 6 5 4 3 2 1 ppm
1.00
1.00
1.05
1.97
3.04
1.01
1.02
3.07
1.12
1.01
1.02
2.01
2.02

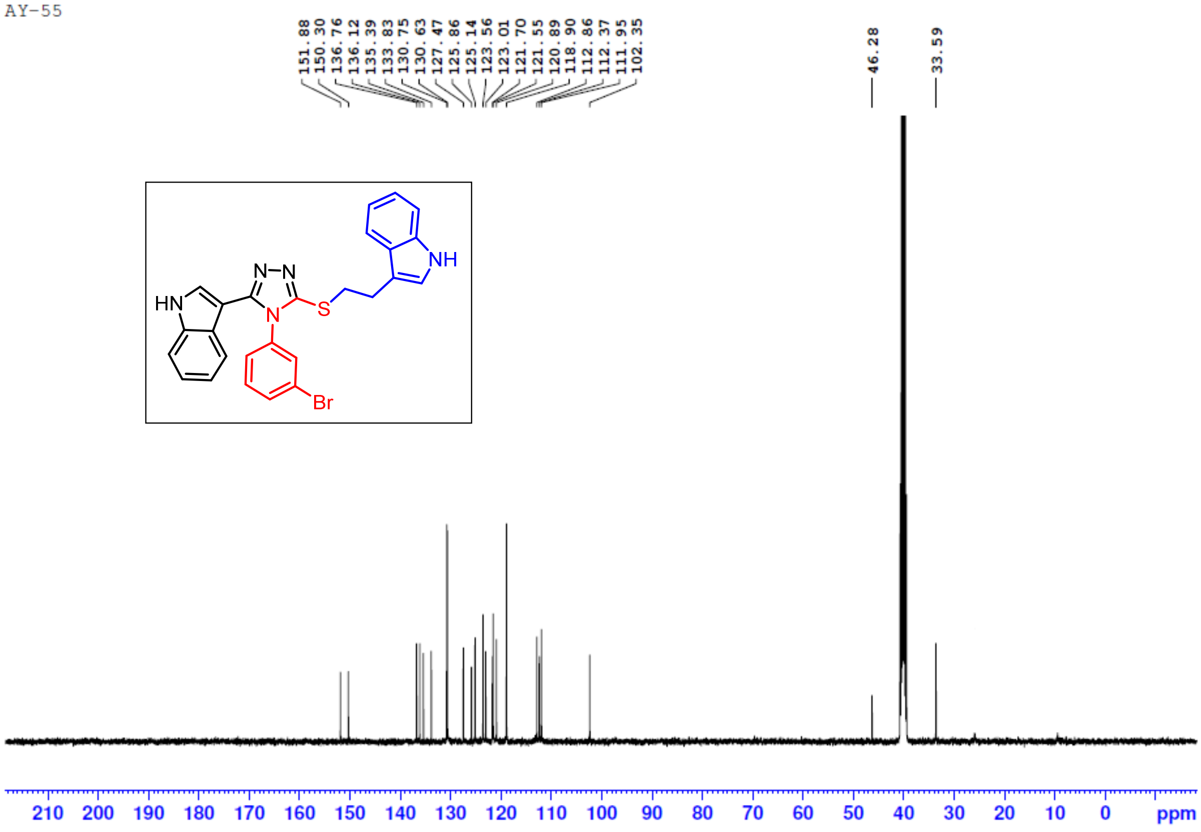
AY-55
151.88
150.30
136.76
136.12
135.39
133.83
130.75
130.63
127.47
125.86
125.14
123.56
123.01
121.70
121.55
120.89
118.90
112.86
112.37
111.95
102.35
46.28
33.59
N–N
HN
N
S
NH
Br
210 200 190 180 170 160 150 140 130 120 110 100 90 80 70 60 50 40 30 20 10 0 ppm

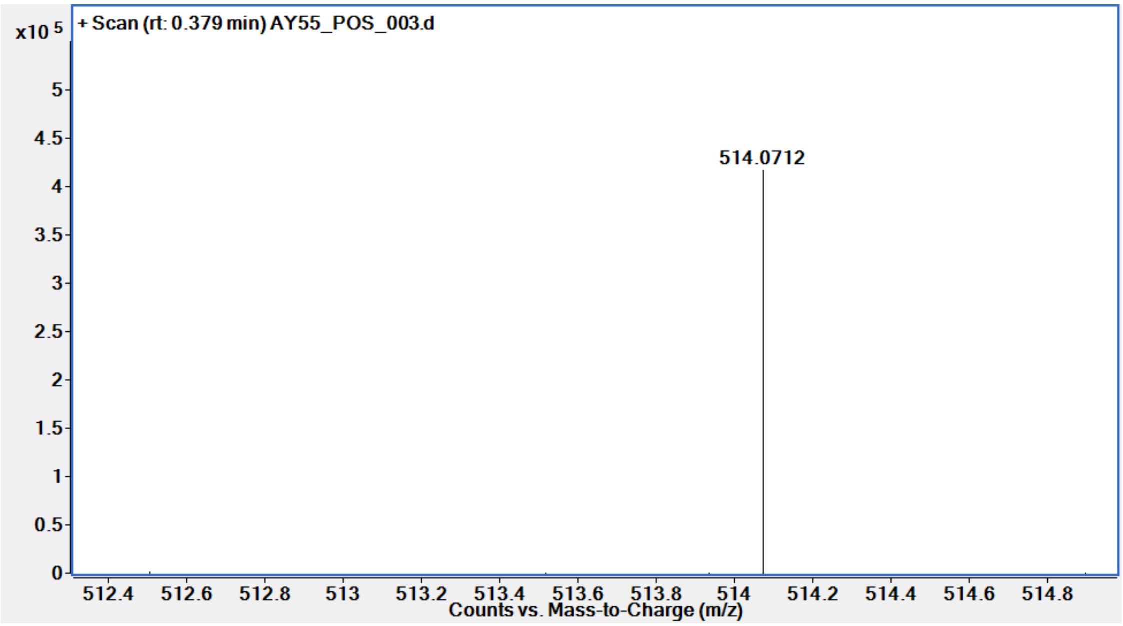
x10 5
+ Scan (rt: 0.379 min) AY55_POS_003.d
514.0712
Counts vs. Mass-to-Charge (m/z)

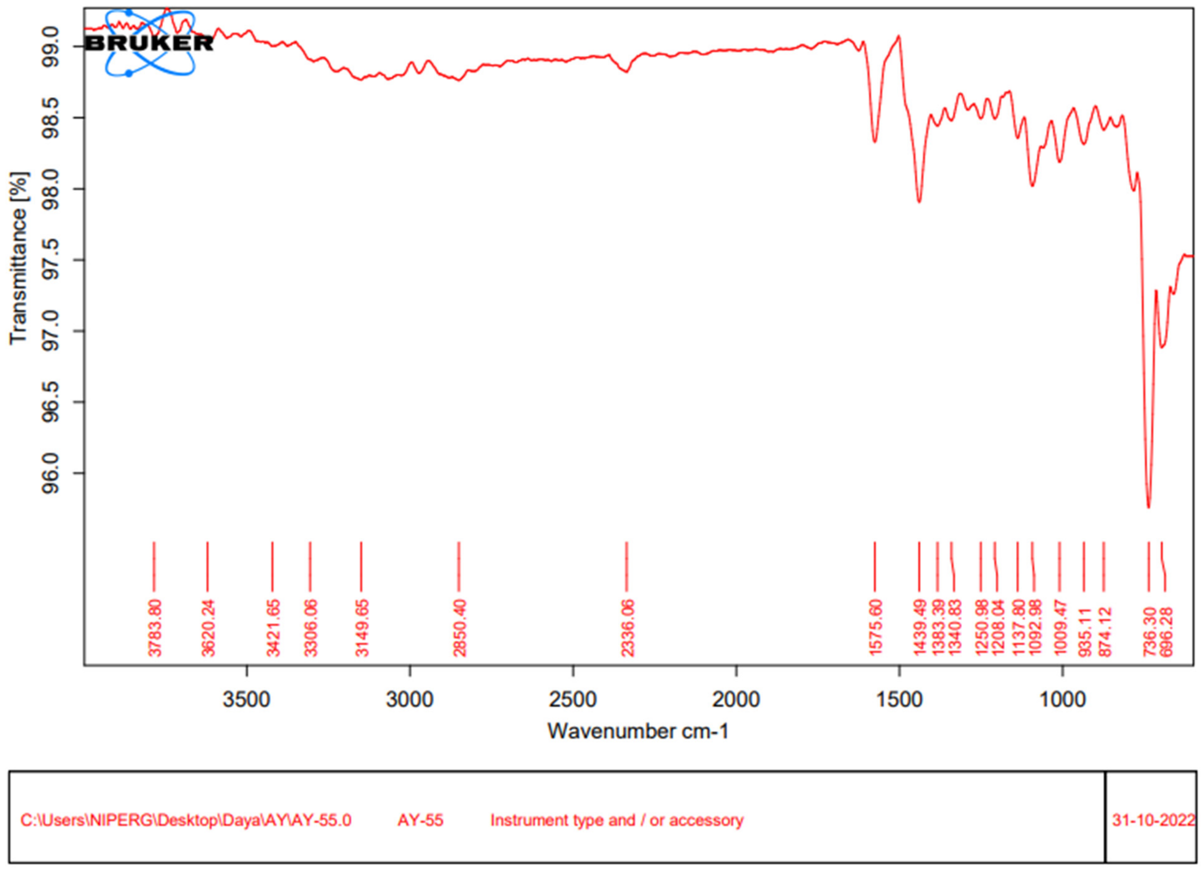
BRUKER
Transmittance [%]
Wavenumber cm-1
3783.80
3620.24
3421.65
3306.06
3149.65
2850.40
2336.06
1575.60
1439.49
1383.39
1340.83
1250.98
1208.04
1137.80
1092.98
1009.47
935.11
874.12
736.30
696.28
C:\Users\NIPERG\Desktop\Daya\AY\AY-55.0
AY-55
Instrument type and / or accessory
31-10-2022

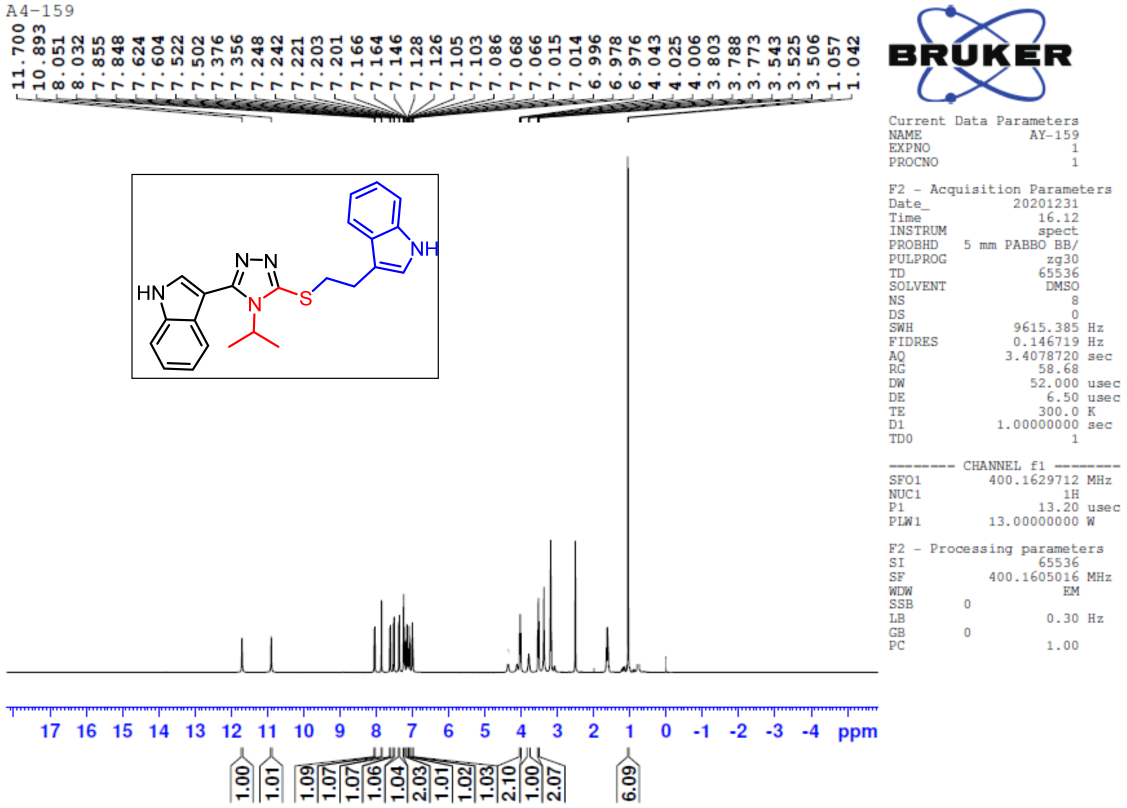
A4-159
11.700
10.893
8.051
8.032
7.855
7.848
7.624
7.604
7.522
7.502
7.376
7.356
7.248
7.242
7.221
7.203
7.201
7.166
7.164
7.146
7.128
7.126
7.105
7.103
7.086
7.068
7.066
7.015
7.014
6.996
6.978
6.976
4.043
4.025
4.006
3.803
3.788
3.773
3.543
3.525
3.506
1.057
1.042
HN
N–N
N
S
NH
BRUKER
Current Data Parameters
NAME AY-159
EXPNO 1
PROCNO 1
F2 - Acquisition Parameters
Date_ 20201231
Time 16.12
INSTRUM spect
PROBHD 5 mm PABBO BB/
PULPROG zg30
TD 65536
SOLVENT DMSO
NS 8
DS 0
SWH 9615.385 Hz
FIDRES 0.146719 Hz
AQ 3.4078720 sec
RG 58.68
DW 52.000 usec
DE 6.50 usec
TE 300.0 K
D1 1.00000000 sec
TD0 1
======== CHANNEL f1 ========
SFO1 400.1629712 MHz
NUC1 1H
P1 13.20 usec
PLW1 13.00000000 W
F2 - Processing parameters
SI 65536
SF 400.1605016 MHz
WDW EM
SSB 0
LB 0.30 Hz
GB 0
PC 1.00
17 16 15 14 13 12 11 10 9 8 7 6 5 4 3 2 1 0 -1 -2 -3 -4 ppm
1.00
1.01
1.09
1.07
1.07
1.06
1.04
2.03
1.01
1.02
1.03
2.10
1.00
2.07
6.09

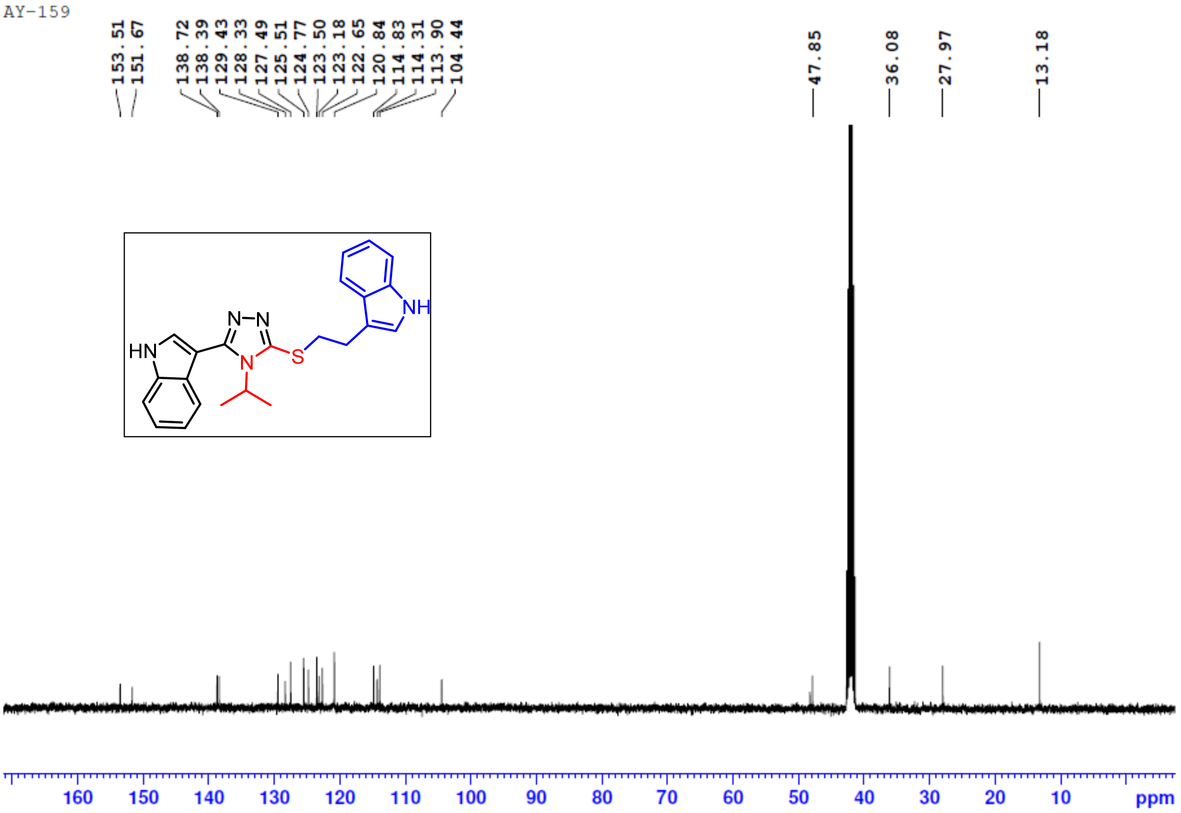
AY-159
153.51
151.67
138.72
138.39
129.43
128.33
127.49
125.51
124.77
123.50
123.18
122.65
120.84
114.83
114.31
113.90
104.44
47.85
36.08
27.97
13.18
HN
N–N
N
S
NH
160 150 140 130 120 110 100 90 80 70 60 50 40 30 20 10 ppm

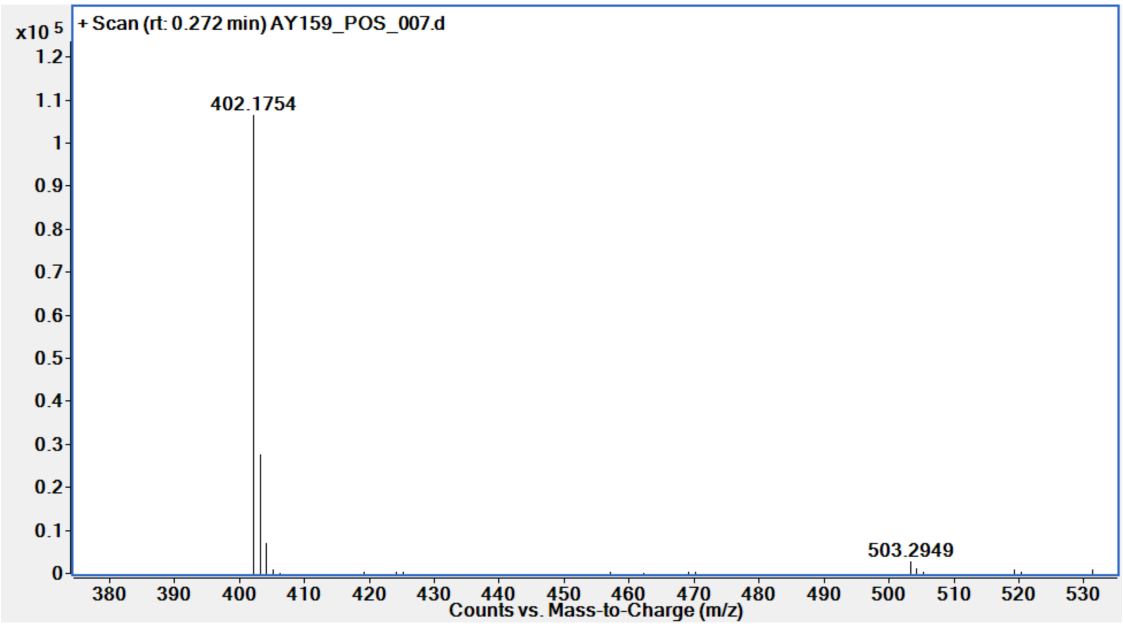
x10 5
+ Scan (rt: 0.272 min) AY159_POS_007.d
402.1754
503.2949
Counts vs. Mass-to-Charge (m/z)

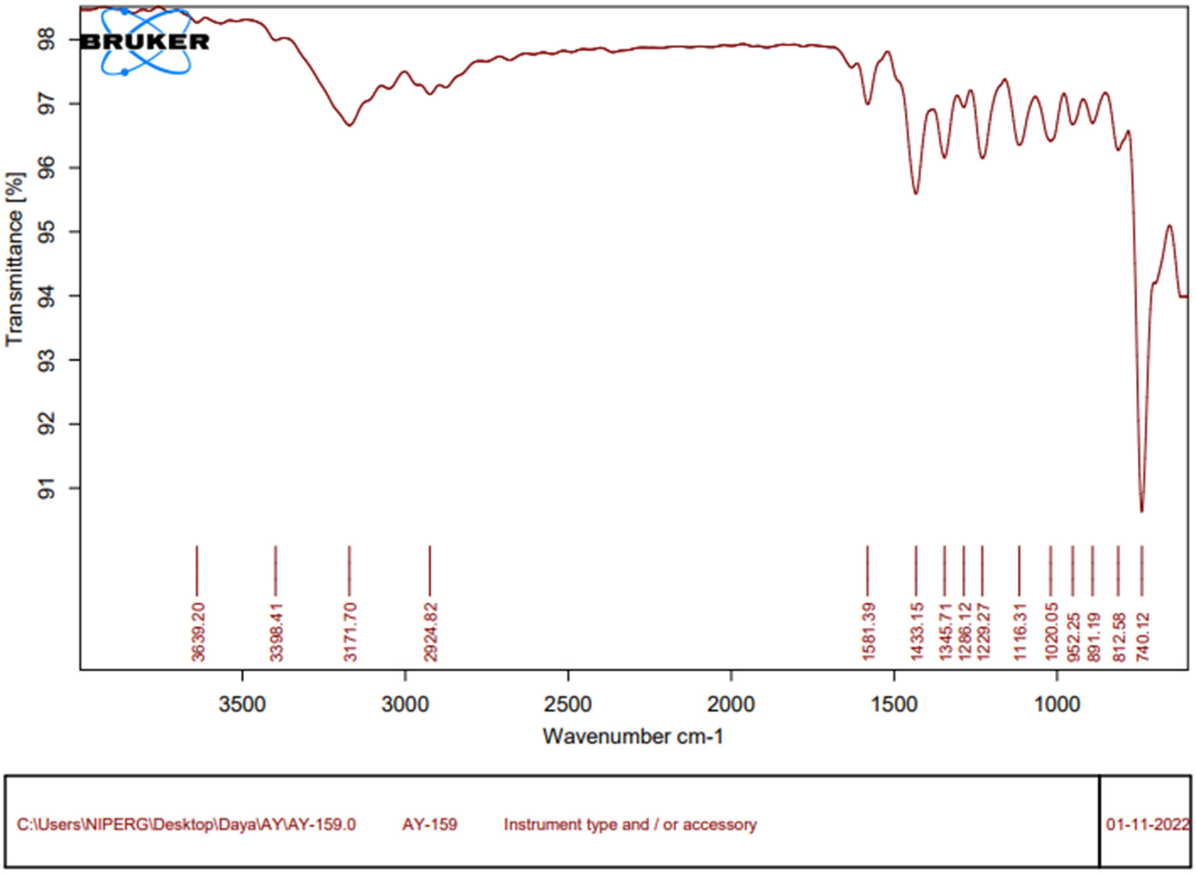
BRUKER
Transmittance [%]
3639.20
3398.41
3171.70
2924.82
1581.39
1433.15
1345.71
1286.12
1229.27
1116.31
1020.05
952.25
891.19
812.58
740.12
Wavenumber cm-1
C:\Users\NIPERG\Desktop\Daya\AY\AY-159.0
AY-159
Instrument type and / or accessory
01-11-2022

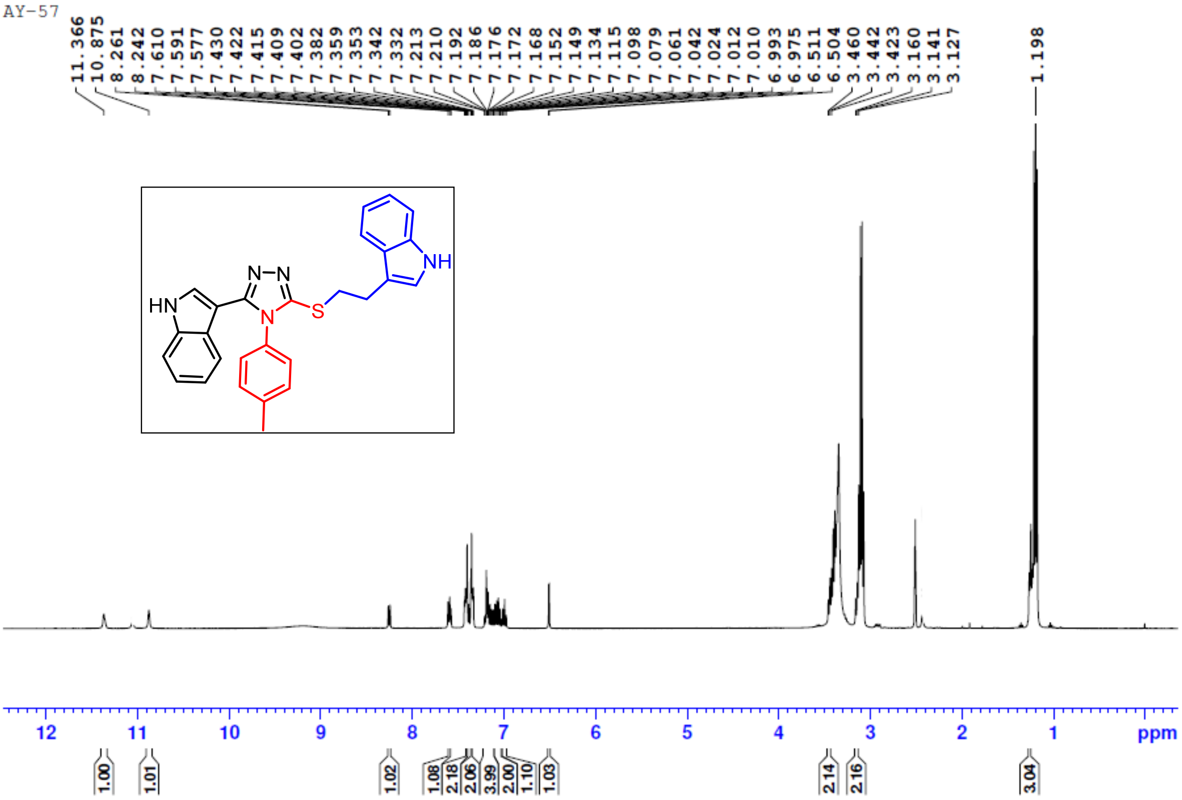

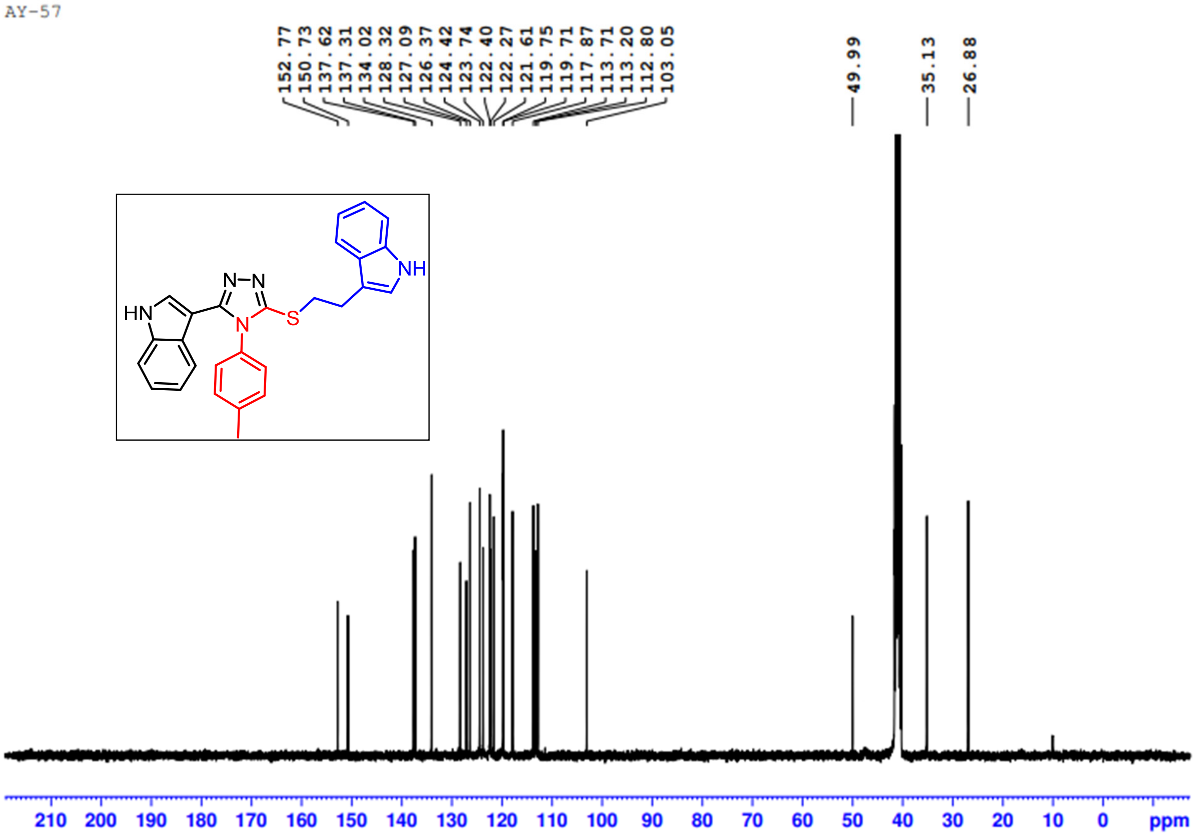

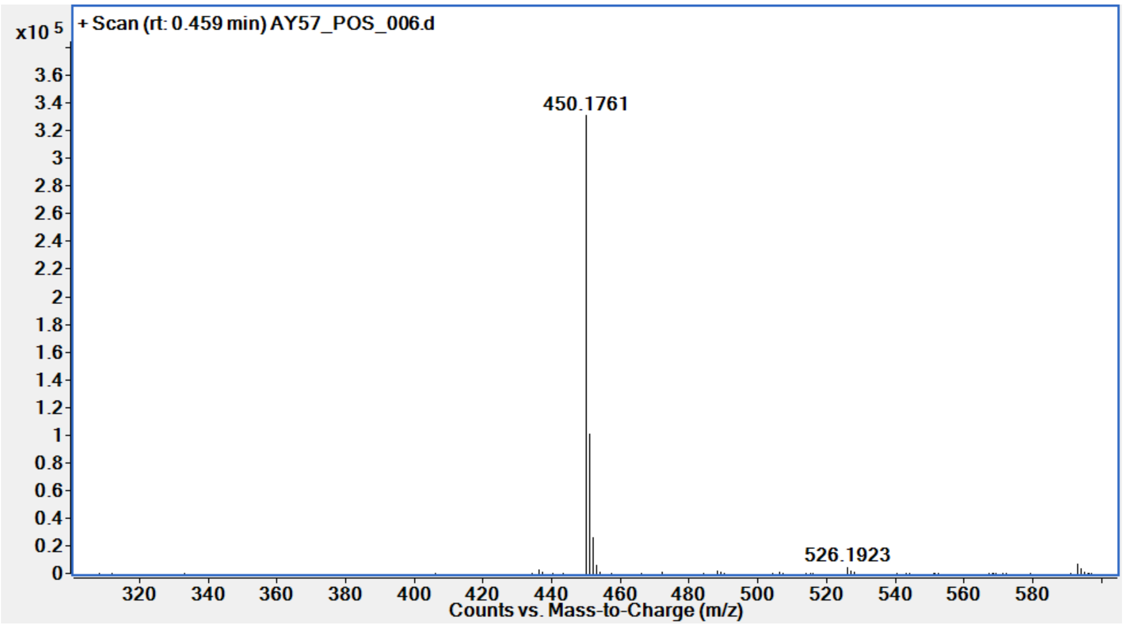

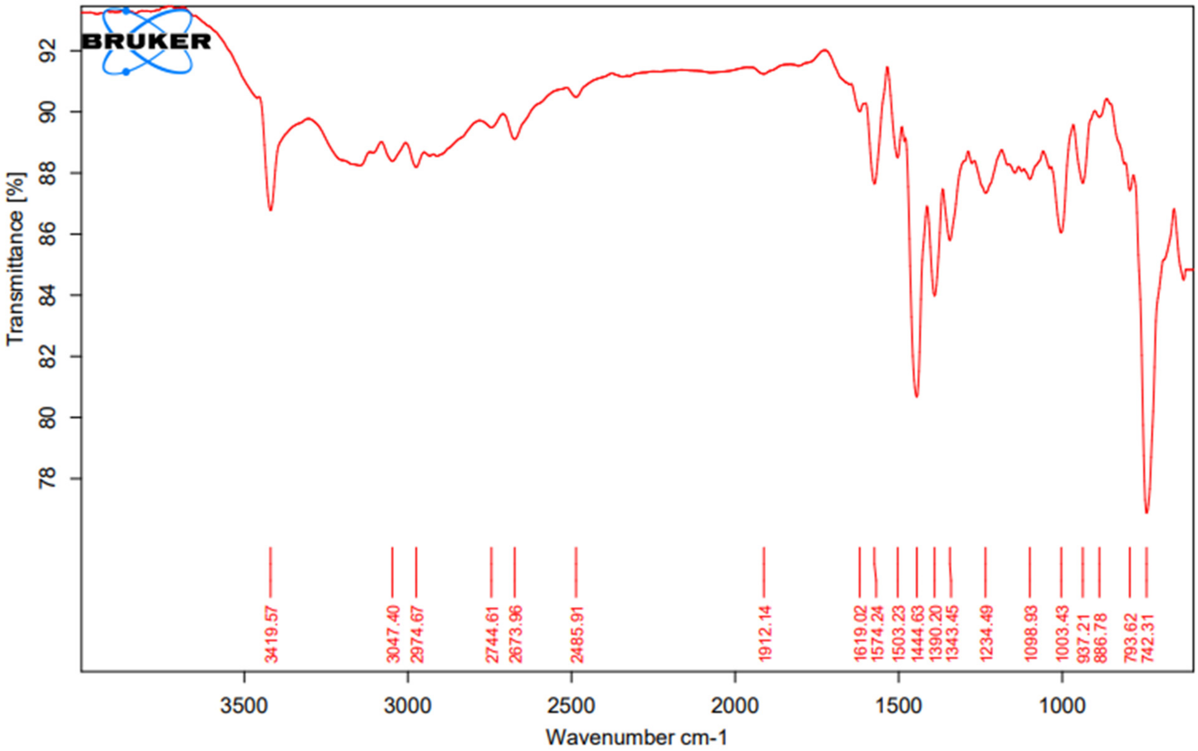

C:\Users\NIPERG\Desktop\Daya\AY\AY-57.0	AY-57	Instrument type and / or accessory	01-11-2022

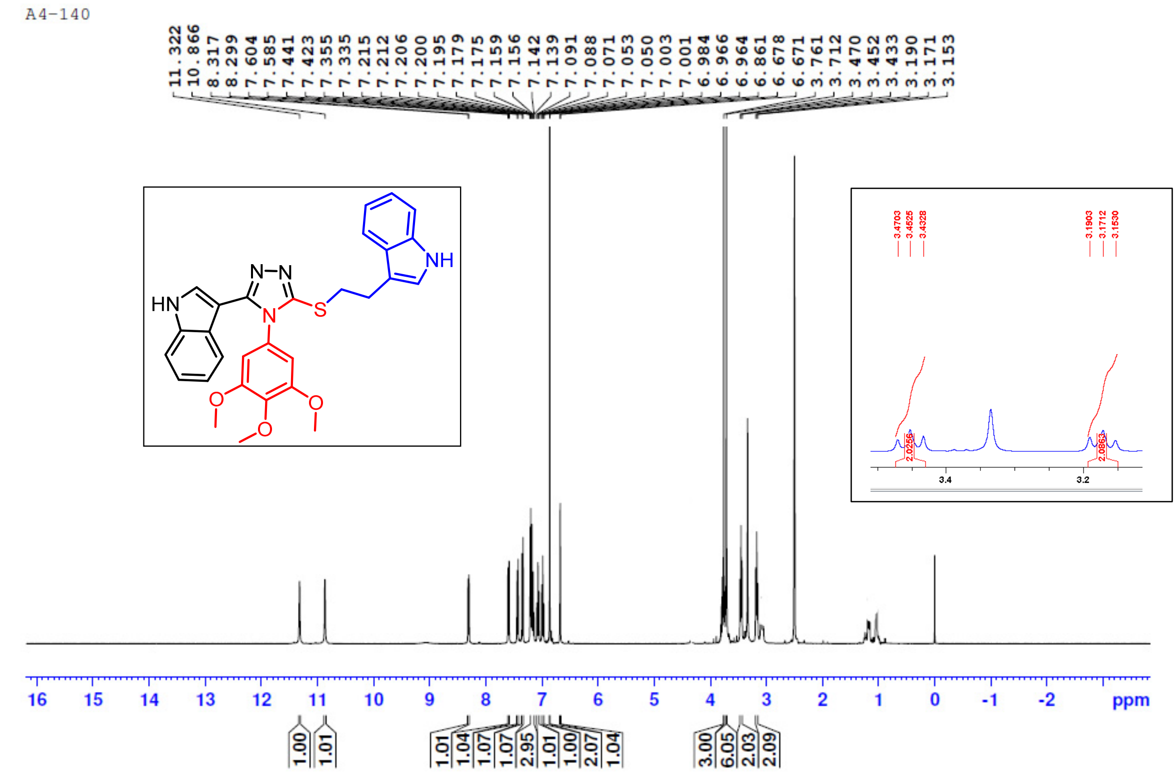
A4-140
11.322
10.866
8.317
8.299
7.604
7.585
7.441
7.423
7.355
7.335
7.215
7.212
7.206
7.200
7.195
7.179
7.175
7.159
7.156
7.142
7.139
7.091
7.088
7.071
7.053
7.050
7.003
7.001
6.984
6.966
6.964
6.861
6.678
6.671
3.761
3.712
3.470
3.452
3.433
3.190
3.171
3.153
HN
N–N
N
S
NH
O
O
O
3.4703
3.4525
3.4328
3.1903
3.1712
3.1530
3.4
3.2
ppm
1.00
1.01
1.01
1.04
1.07
1.07
2.95
1.01
1.00
2.07
1.04
3.00
6.05
2.03
2.09

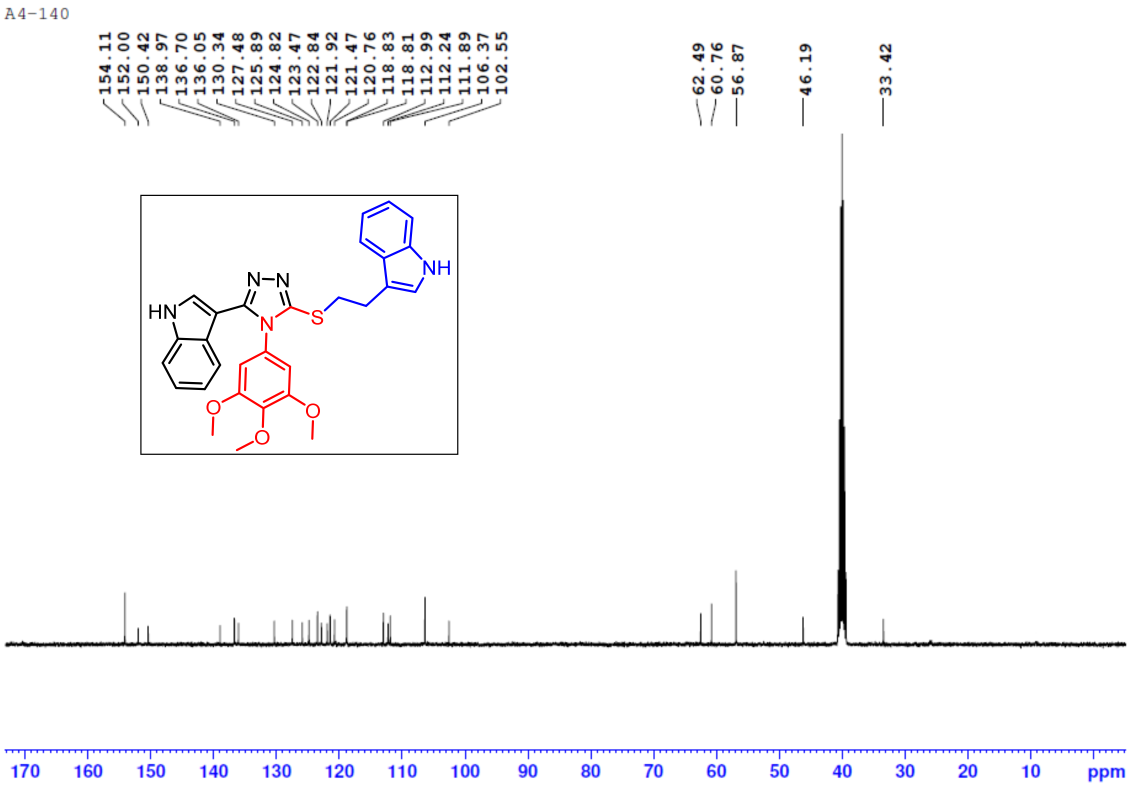
A4-140
154.11
152.00
150.42
138.97
136.70
136.05
130.34
127.48
125.89
124.82
123.47
122.84
121.92
121.47
120.76
118.83
118.81
112.99
112.24
111.89
106.37
102.55
62.49
60.76
56.87
46.19
33.42
HN
N–N
N
S
NH
O
O
O
ppm

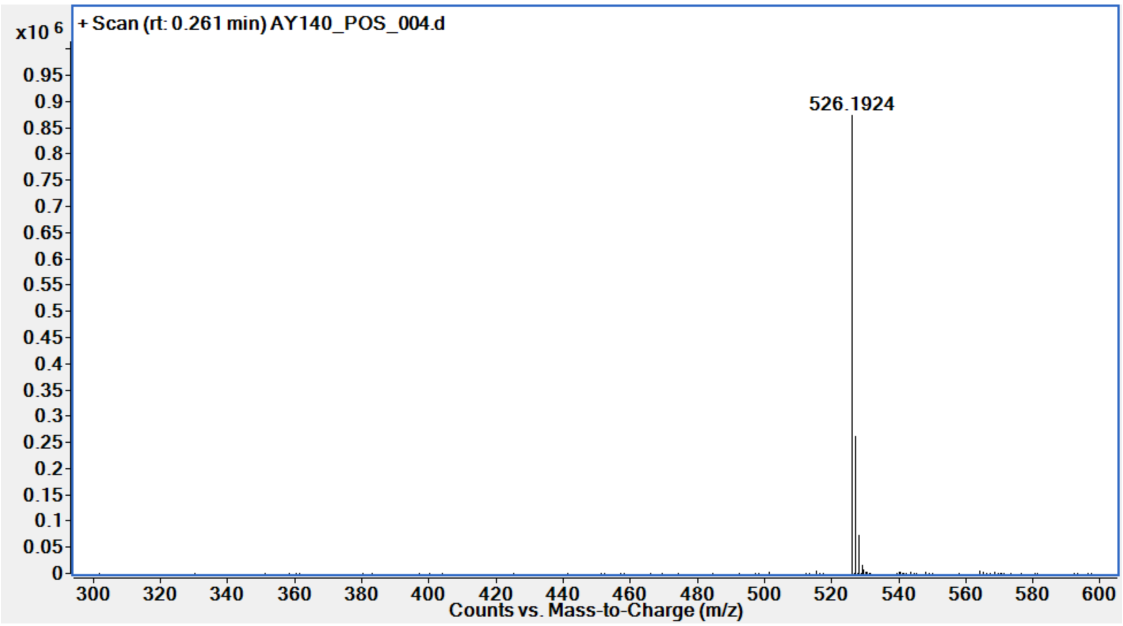
+ Scan (rt: 0.261 min) AY140_POS_004.d
526.1924
Counts vs. Mass-to-Charge (m/z)

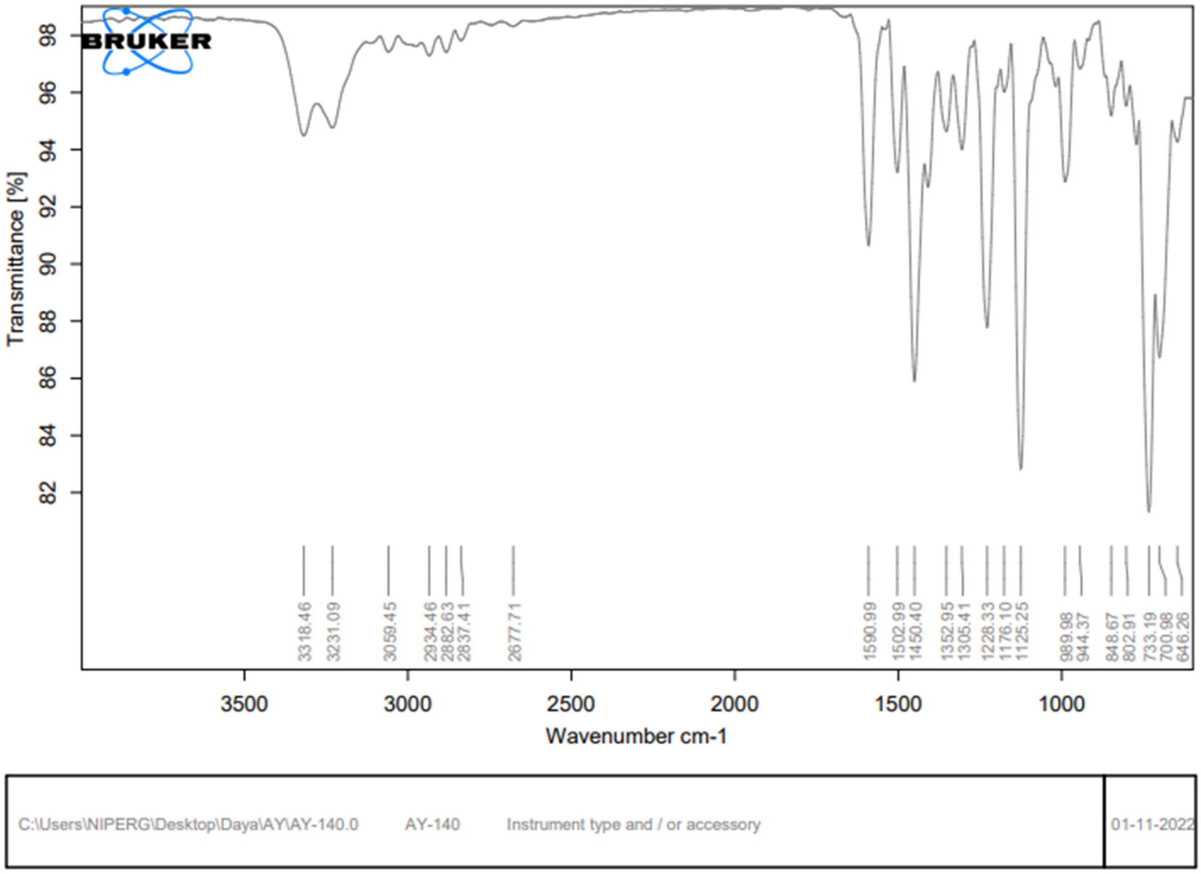
BRUKER
Transmittance [%]
Wavenumber cm-1
3318.46
3231.09
3059.45
2934.46
2882.63
2837.41
2677.71
1590.99
1502.99
1450.40
1352.95
1305.41
1228.33
1176.10
1125.25
989.98
944.37
848.67
802.91
733.19
700.98
646.26
C:\Users\NIPERG\Desktop\Daya\AY\AY-140.0
AY-140
Instrument type and / or accessory
01-11-2022

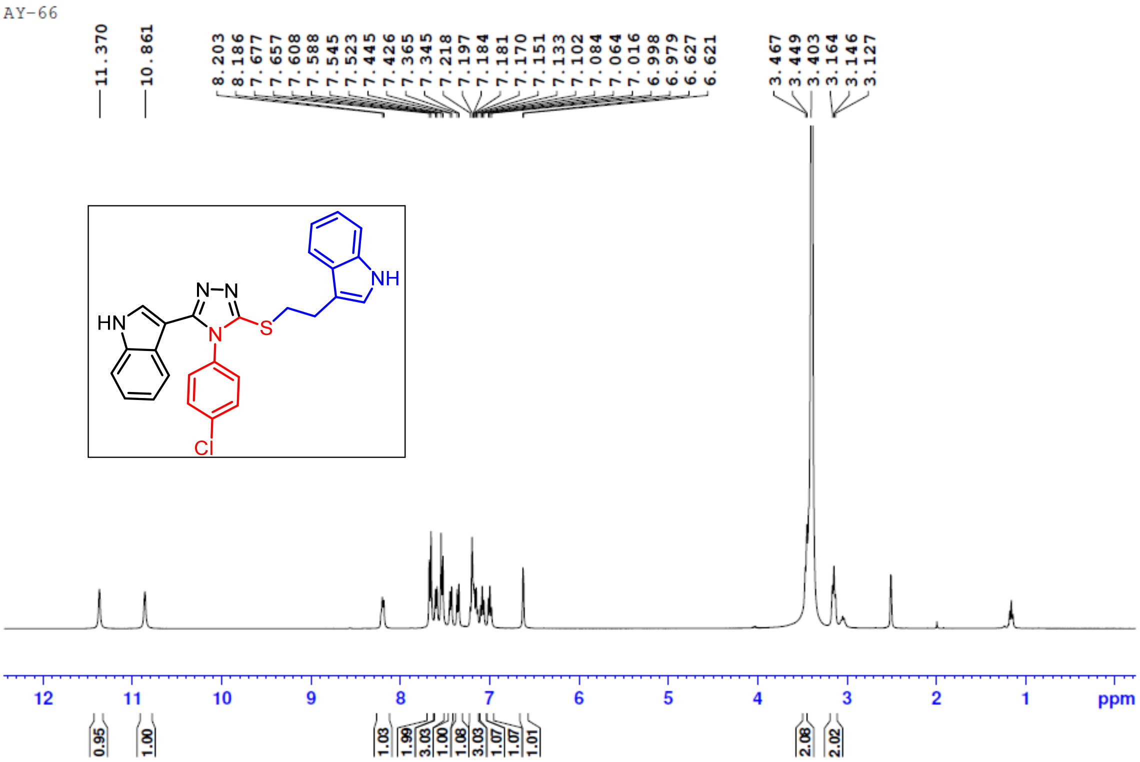
AY-66
11.370
10.861
8.203
8.186
7.677
7.657
7.608
7.588
7.545
7.523
7.445
7.426
7.365
7.345
7.218
7.197
7.184
7.181
7.170
7.151
7.133
7.102
7.084
7.064
7.016
6.998
6.979
6.627
6.621
3.467
3.449
3.403
3.164
3.146
3.127
HN
N–N
N
S
NH
Cl
ppm
0.95
1.00
1.03
1.99
3.03
1.00
1.08
3.03
1.07
1.07
1.01
2.08
2.02

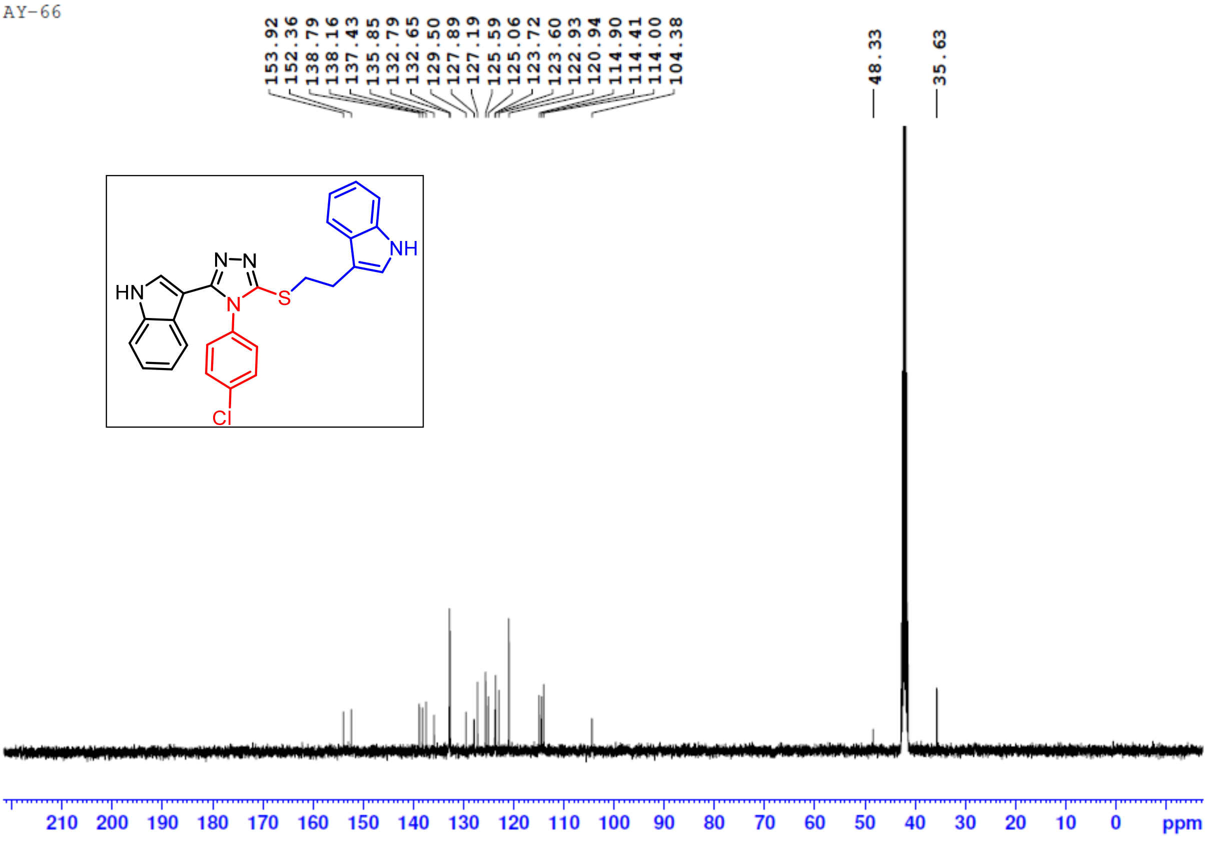
AY-66
153.92
152.36
138.79
138.16
137.43
135.85
132.79
132.65
129.50
127.89
127.19
125.59
125.06
123.72
123.60
122.93
120.94
114.90
114.41
114.00
104.38
48.33
35.63
HN
N–N
N
S
NH
Cl
ppm

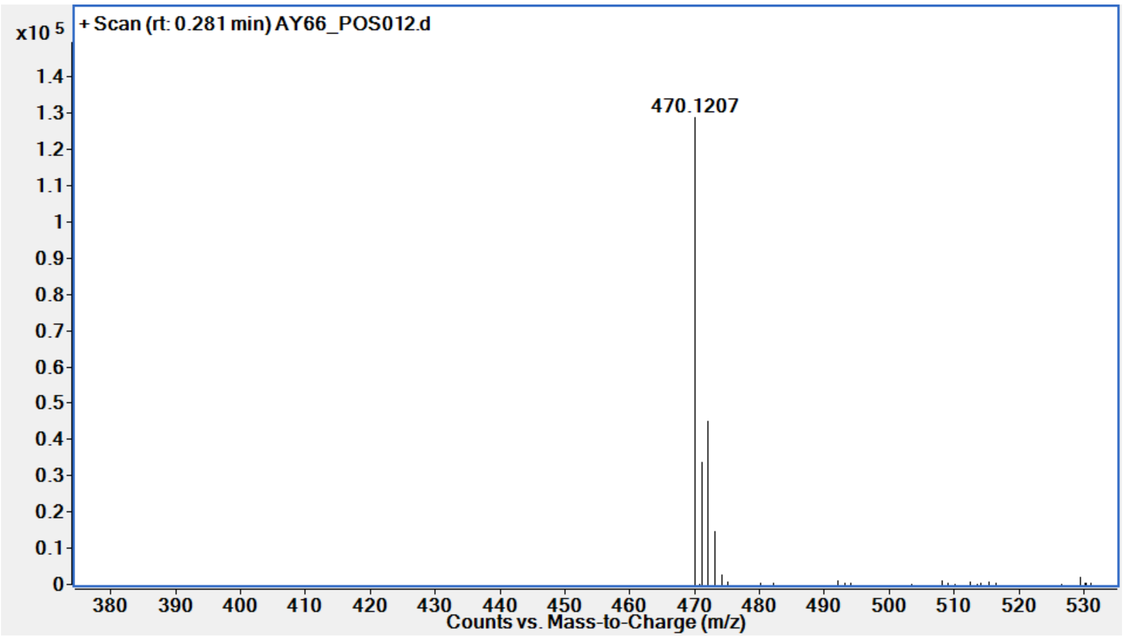

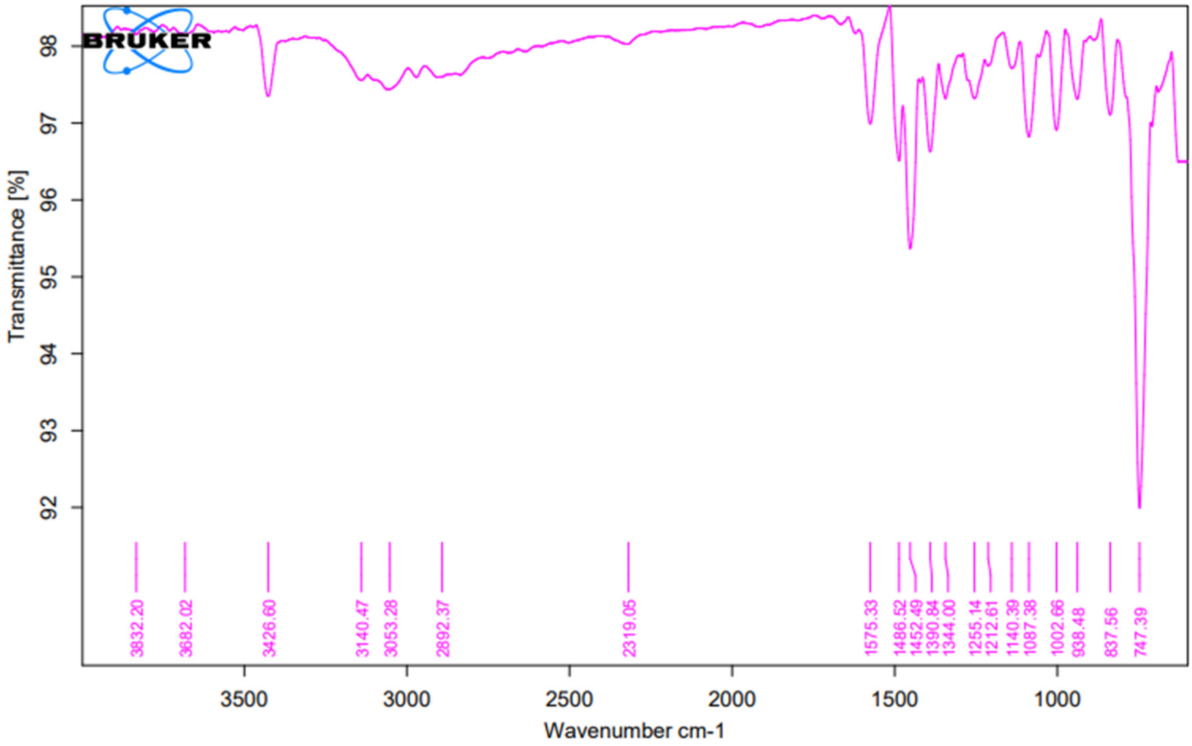

C:\Users\NIPERG\Desktop\Daya\AY\AY-66.0	AY-66	Instrument type and / or accessory	31-10-2022

AY-141

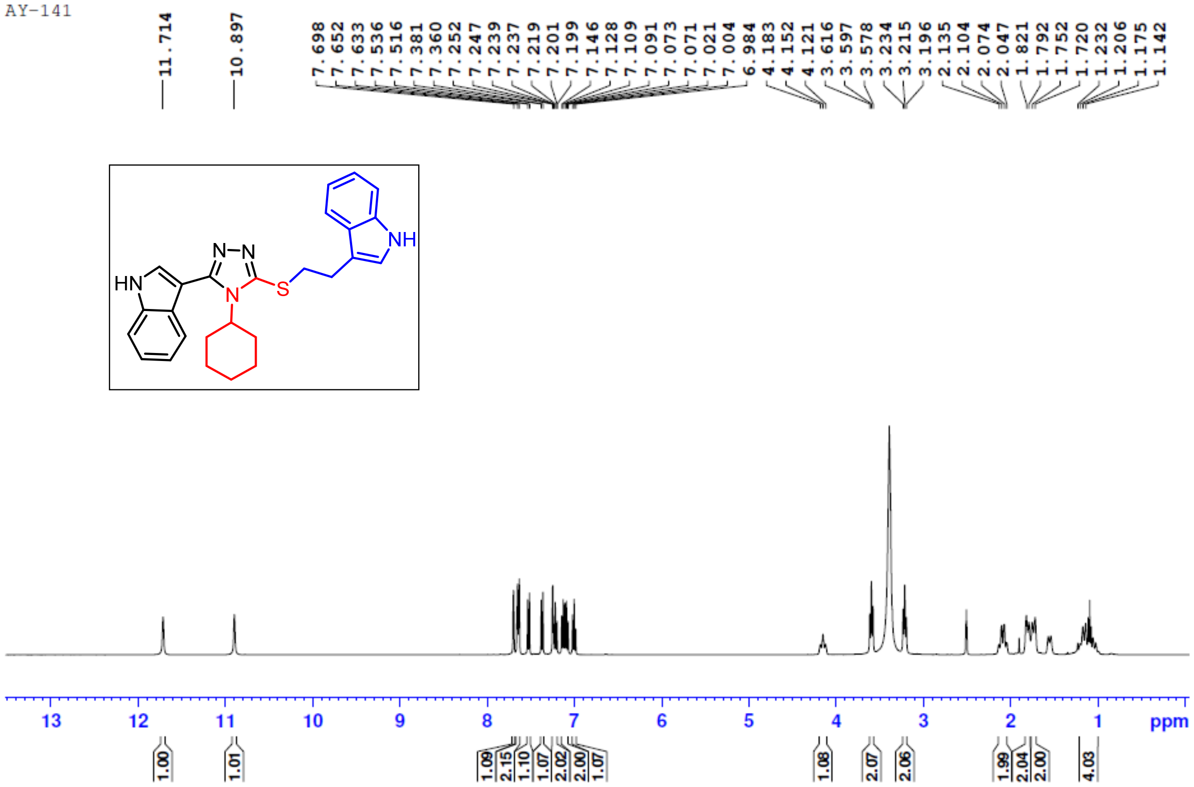

AY-141

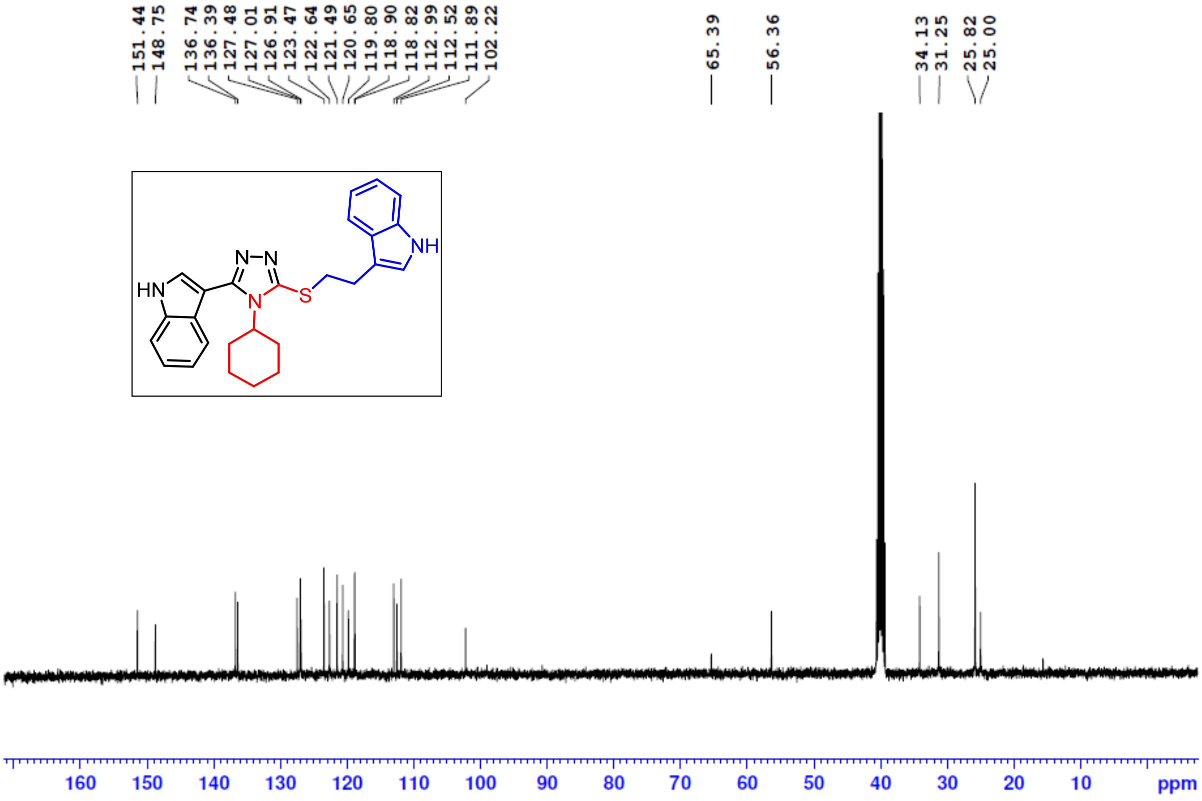

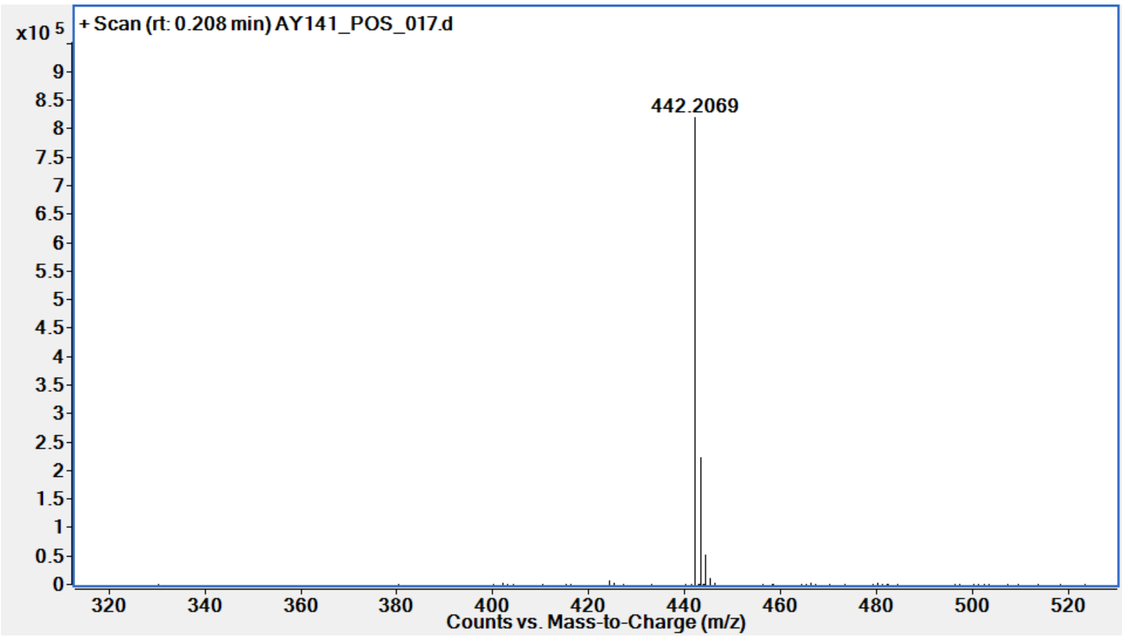

x10 5
+ Scan (rt: 0.208 min) AY141_POS_017.d
442.2069
Counts vs. Mass-to-Charge (m/z)

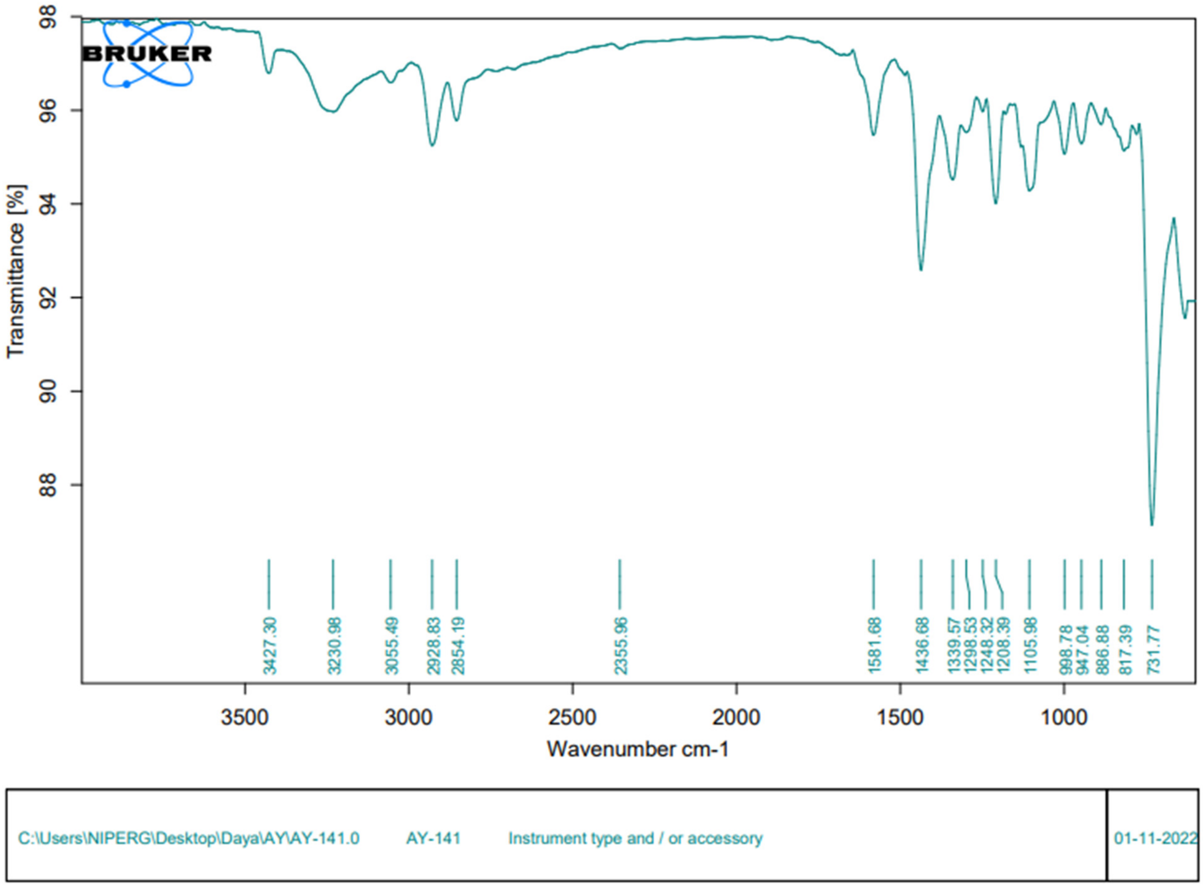

BRUKER
Transmittance [%]
Wavenumber cm-1
3427.30
3230.98
3055.49
2928.83
2854.19
2355.96
1581.68
1436.68
1339.57
1298.53
1248.32
1208.39
1105.98
998.78
947.04
886.88
817.39
731.77
C:\Users\NIPERG\Desktop\Daya\AY\AY-141.0
AY-141
Instrument type and / or accessory
01-11-2022

AY-35

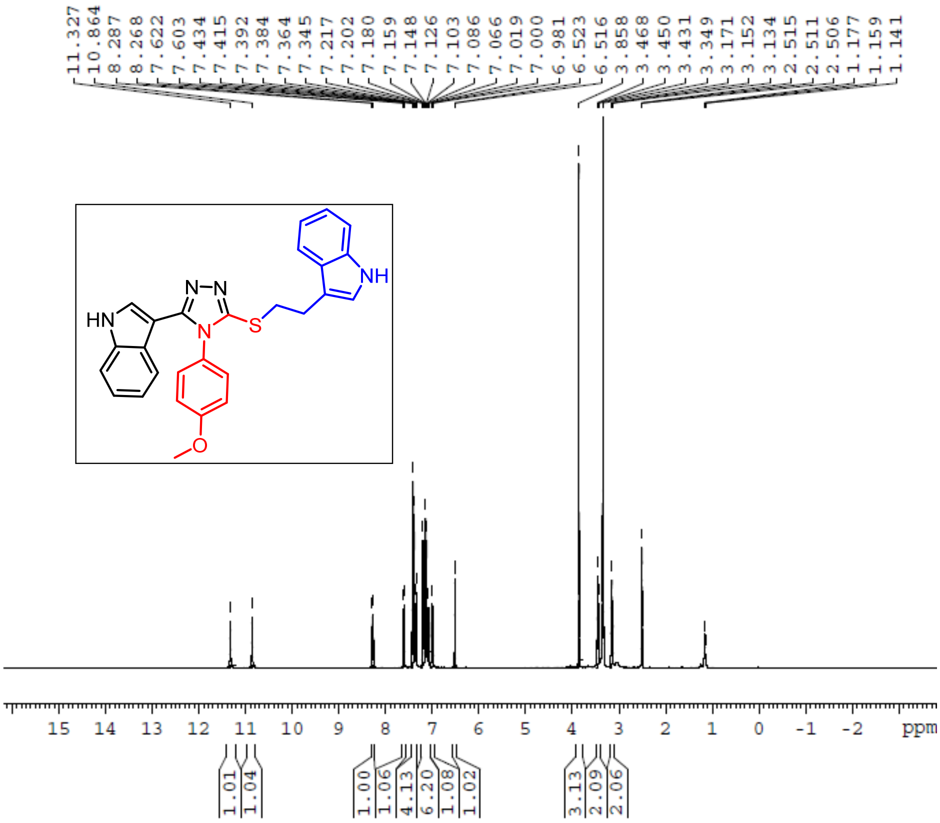

AY-35

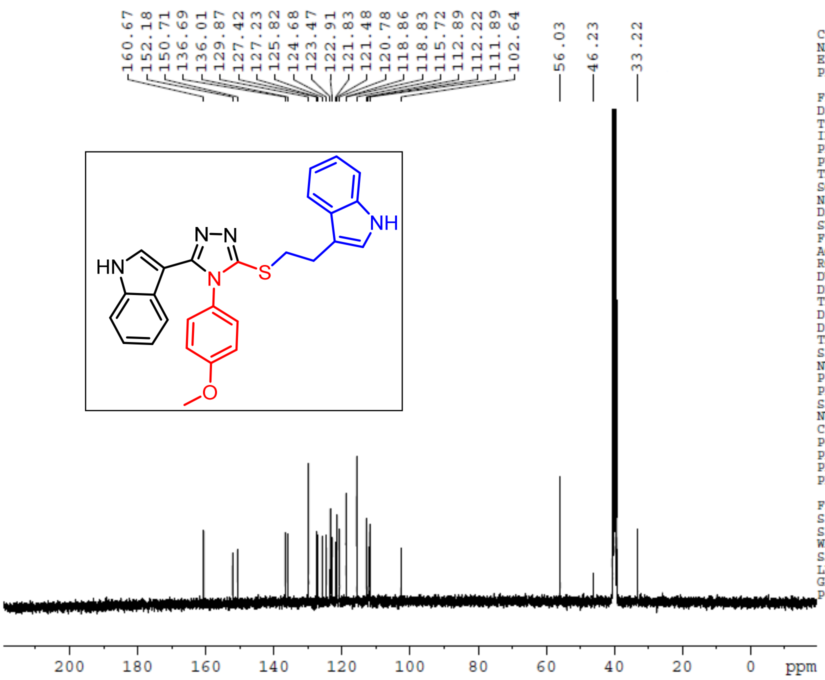

BRUKER

Current Data Parameters
NAME 21012021
EXPNO 12
PROCNO 1

F2 - Acquisition Parameters
Date_ 20210121
Time 18.31 h
INSTRUM spect
PROBHD Z108618_0646 (
PULPROG zgpg30
TD 65536
SOLVENT DMSO
NS 1024
DS 4
SWH 24038.461 Hz
FIDRES 0.733596 Hz
AQ 1.3631488 sec
RG 204
DW 20.800 usec
DE 6.50 usec
TE 298.0 K
D1 2.00000000 sec
D11 0.03000000 sec
TD0 1
SFO1 100.6228298 MHz
NUC1 13C
P1 10.00 usec
PLW1 48.70600128 W
SFO2 400.1316005 MHz
NUC2 1H
CPDPRG[2 waltz16
PCPD2 90.00 usec
PLW2 9.90270042 W
PLW12 0.27508000 W
PLW13 0.13835999 W

F2 - Processing parameters
SI 32768
SF 100.6127685 MHz
WDW EM
SSB 0
LB 1.00 Hz
GB 0
PC 1.40

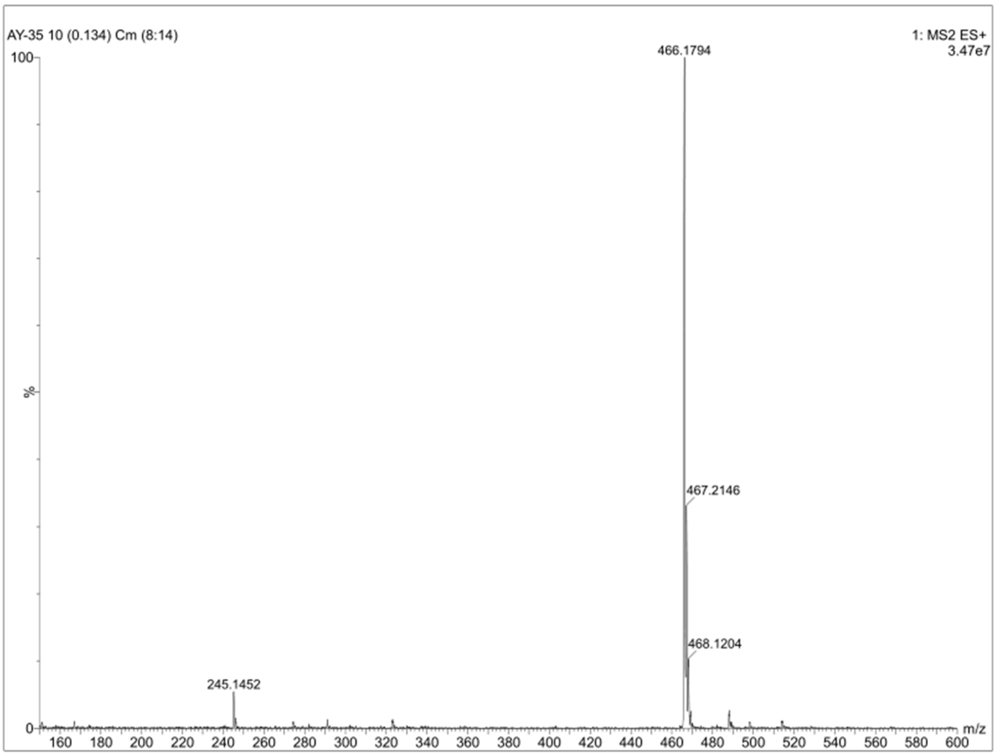

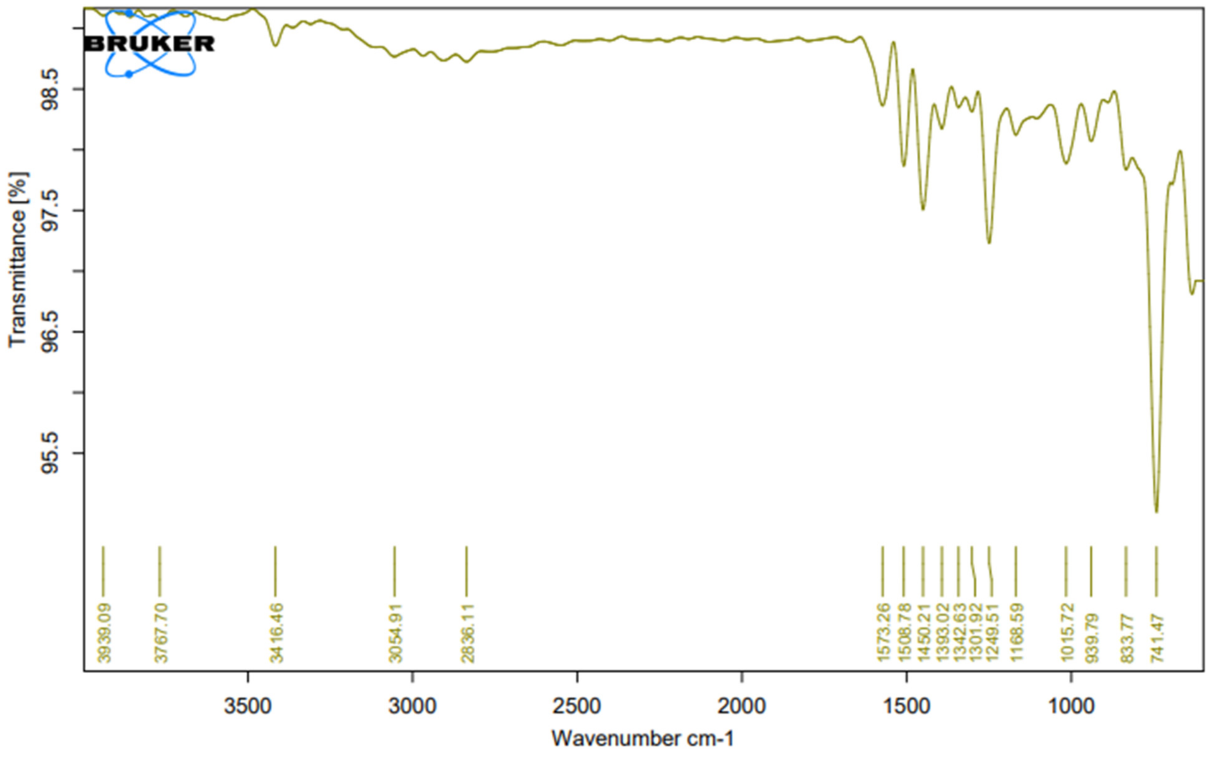

C:\Users\NIPERG\Desktop\Daya\AY\AY-35.0 AY-35 Instrument type and / or accessory	01-11-2022

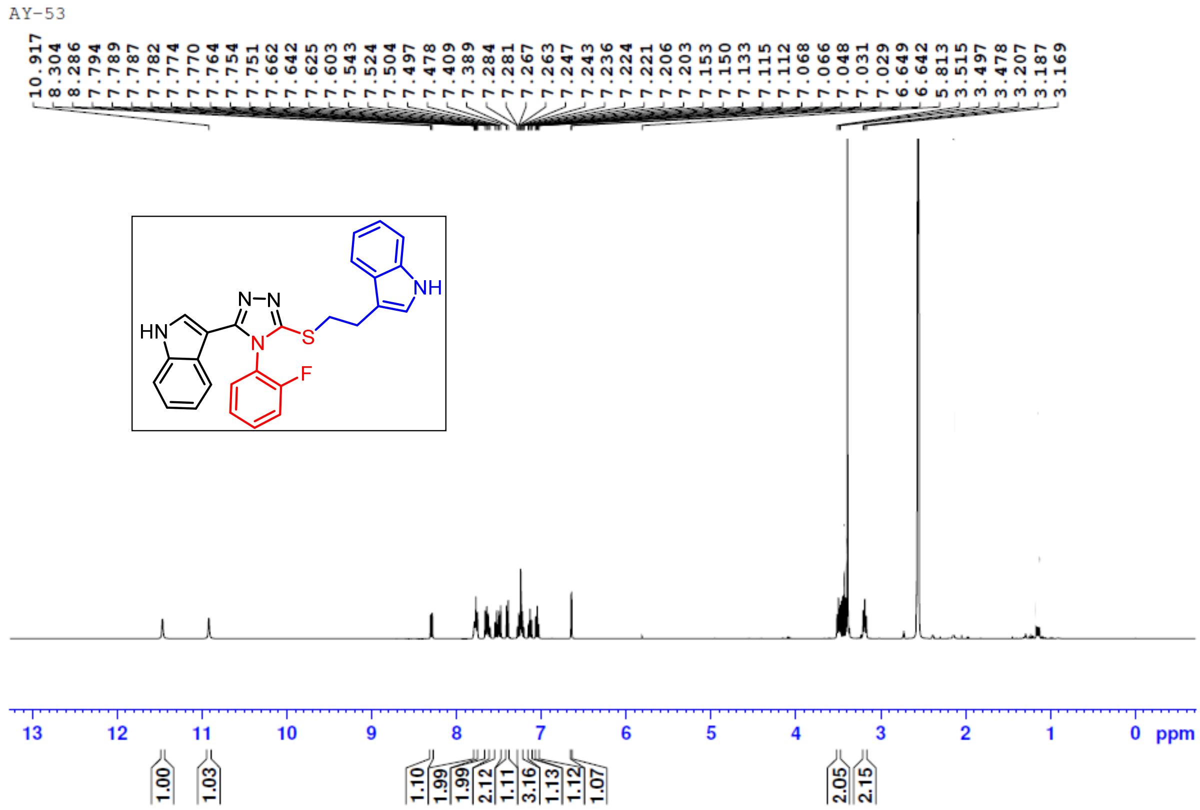
AY-53
10.917 8.304 8.286 7.794 7.789 7.787 7.782 7.774 7.770 7.764 7.754 7.751 7.662 7.642 7.625 7.603 7.543 7.524 7.504 7.497 7.478 7.409 7.389 7.284 7.281 7.267 7.263 7.247 7.243 7.236 7.224 7.221 7.206 7.203 7.153 7.150 7.133 7.115 7.112 7.068 7.066 7.048 7.031 7.029 6.649 6.642 5.813 3.515 3.497 3.478 3.207 3.187 3.169
HN
N–N
NH
S
N
F
13 12 11 10 9 8 7 6 5 4 3 2 1 0 ppm
1.00 1.03 1.10 1.99 1.99 2.12 1.11 3.16 1.13 1.12 1.07 2.05 2.15

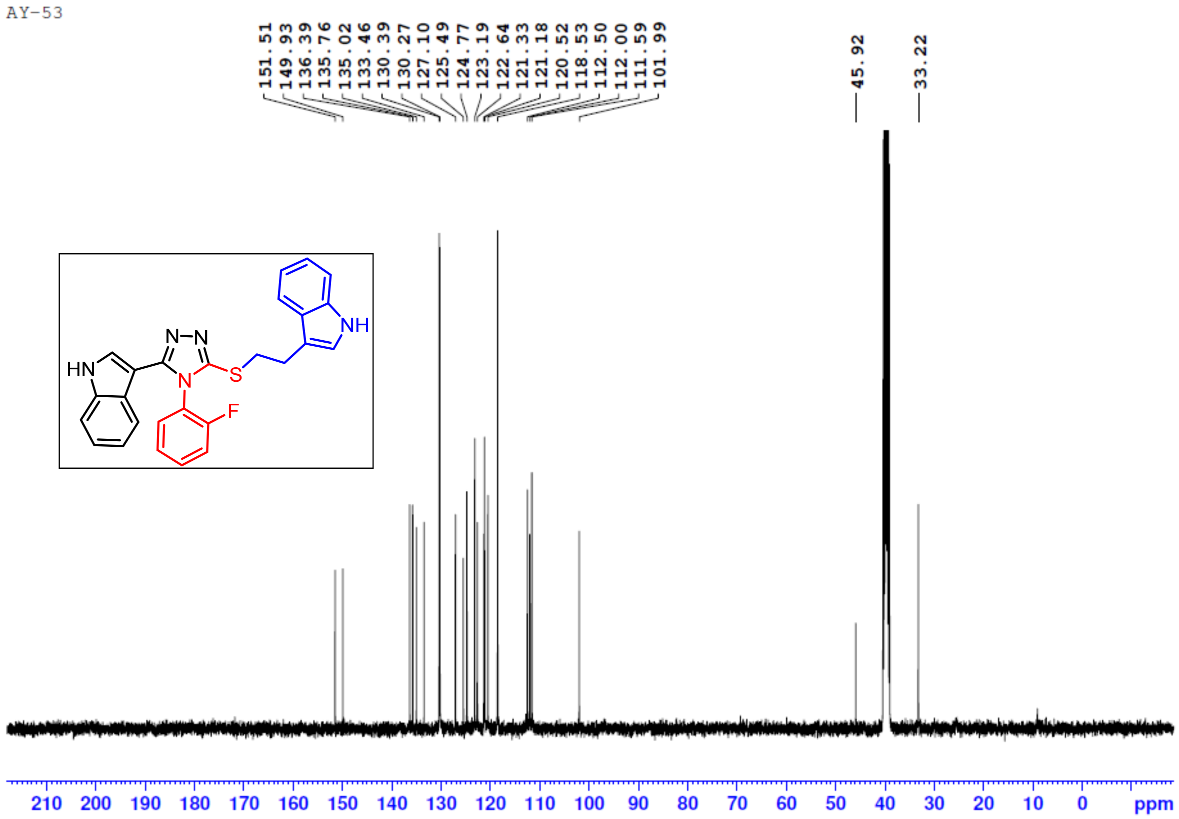
AY-53
151.51 149.93 136.39 135.76 135.02 133.46 130.39 130.27 127.10 125.49 124.77 123.19 122.64 121.33 121.18 120.52 118.53 118.50 112.00 111.59 101.99
45.92
33.22
HN
N–N
NH
S
N
F
210 200 190 180 170 160 150 140 130 120 110 100 90 80 70 60 50 40 30 20 10 0 ppm

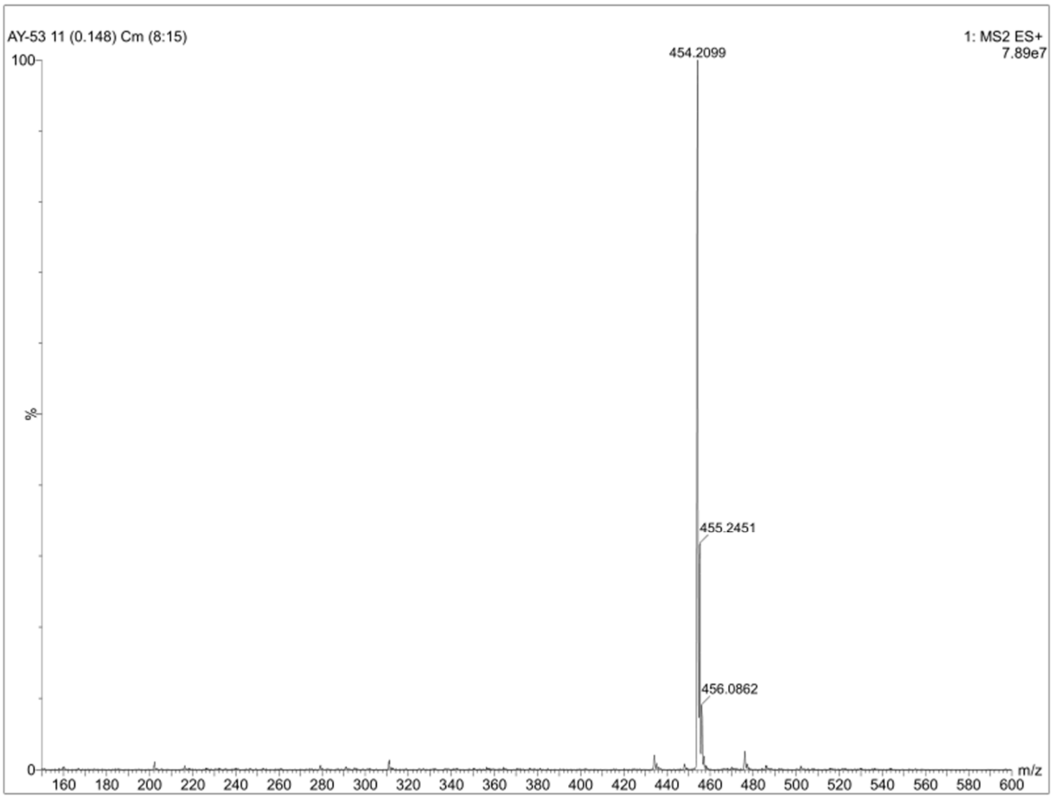
AY-53 11 (0.148) Cm (8:15)
1: MS2 ES+
7.89e7
454.2099
455.2451
456.0862
%
m/z

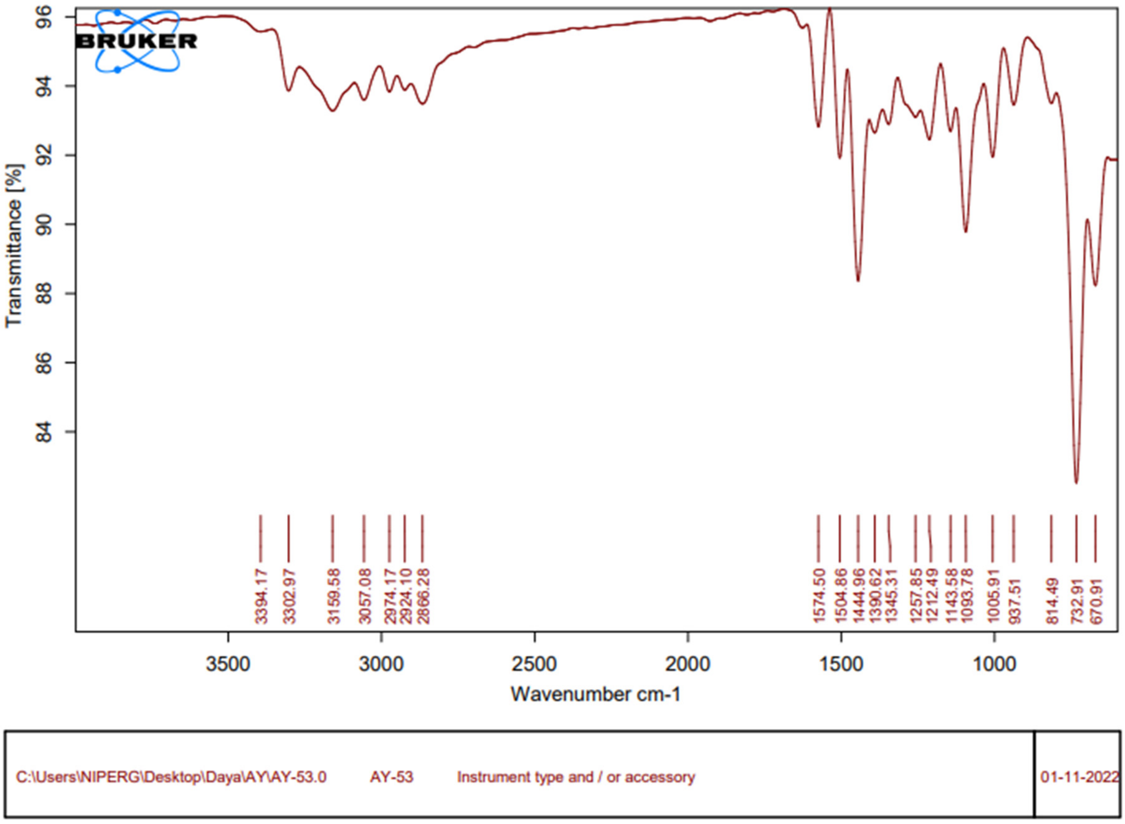
BRUKER
Transmittance [%]
Wavenumber cm-1
3394.17
3302.97
3159.58
3057.08
2974.17
2924.10
2866.28
1574.50
1504.86
1444.96
1390.62
1345.31
1257.85
1212.49
1143.58
1093.78
1005.91
937.51
814.49
732.91
670.91
C:\Users\NIPERG\Desktop\Daya\AY\AY-53.0
AY-53
Instrument type and / or accessory
01-11-2022

AY-52

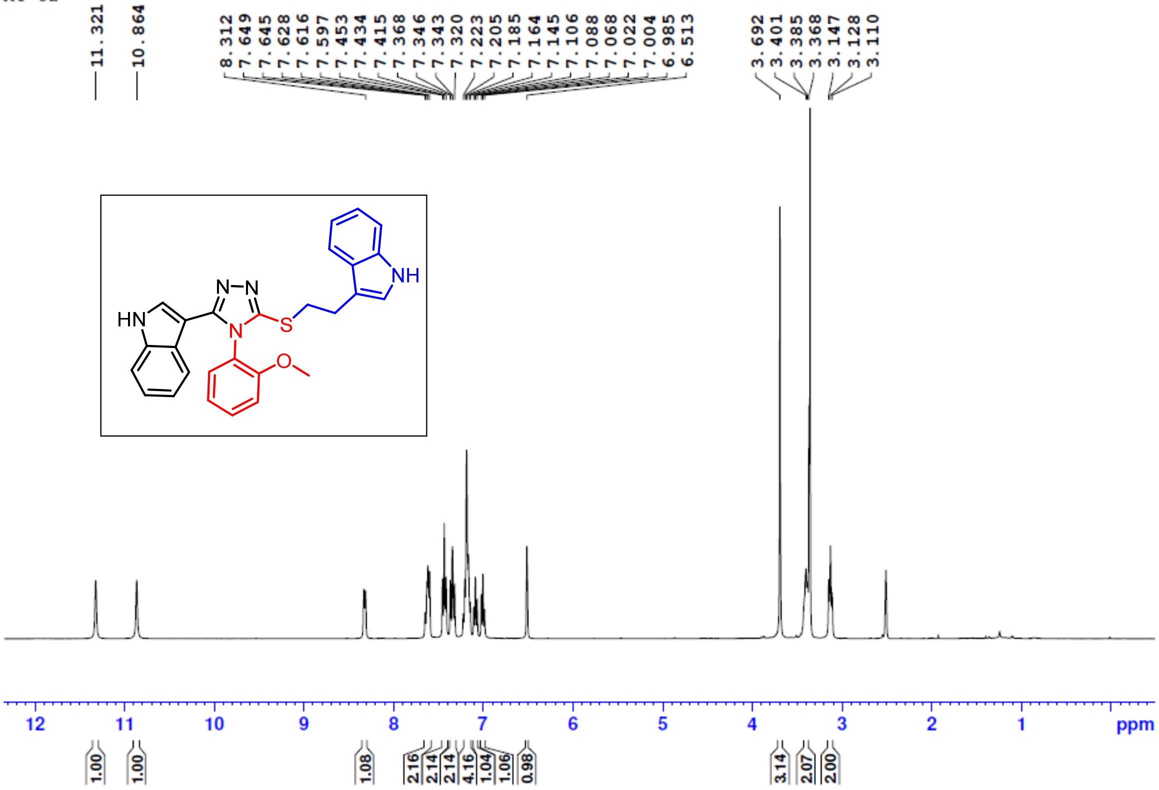
11.321
10.864
8.312
7.649
7.645
7.628
7.616
7.597
7.453
7.434
7.415
7.368
7.346
7.343
7.320
7.223
7.205
7.185
7.164
7.145
7.106
7.088
7.068
7.022
7.004
6.985
6.513
3.692
3.401
3.385
3.368
3.147
3.128
3.110
HN
N–N
N
S
O
NH
12
11
10
9
8
7
6
5
4
3
2
1
ppm
1.00
1.00
1.08
2.16
2.14
2.14
4.16
1.04
1.06
0.98
3.14
2.07
2.00

AY-52

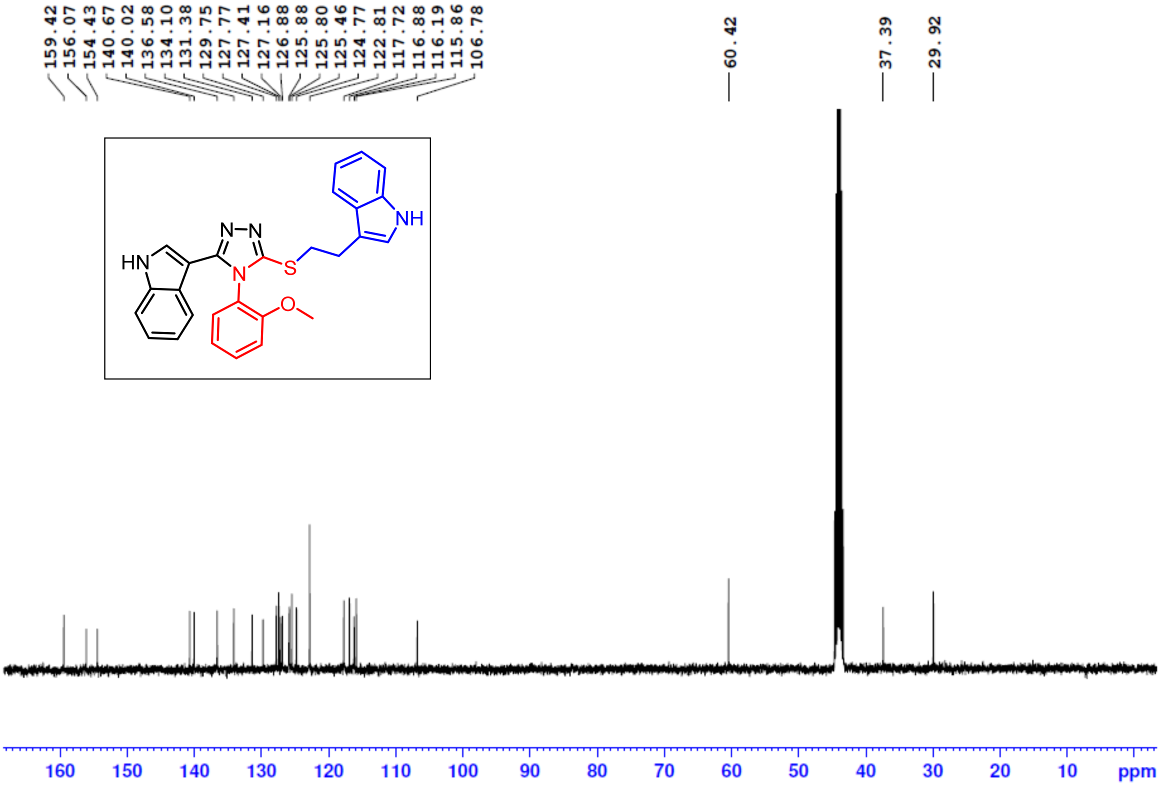
159.42
156.07
154.43
140.67
140.02
136.58
134.10
131.38
129.75
127.77
127.41
127.16
126.88
125.88
125.80
125.46
124.77
122.81
117.72
116.88
116.19
115.86
106.78
60.42
37.39
29.92
HN
N–N
N
S
O
NH
160
150
140
130
120
110
100
90
80
70
60
50
40
30
20
10
ppm

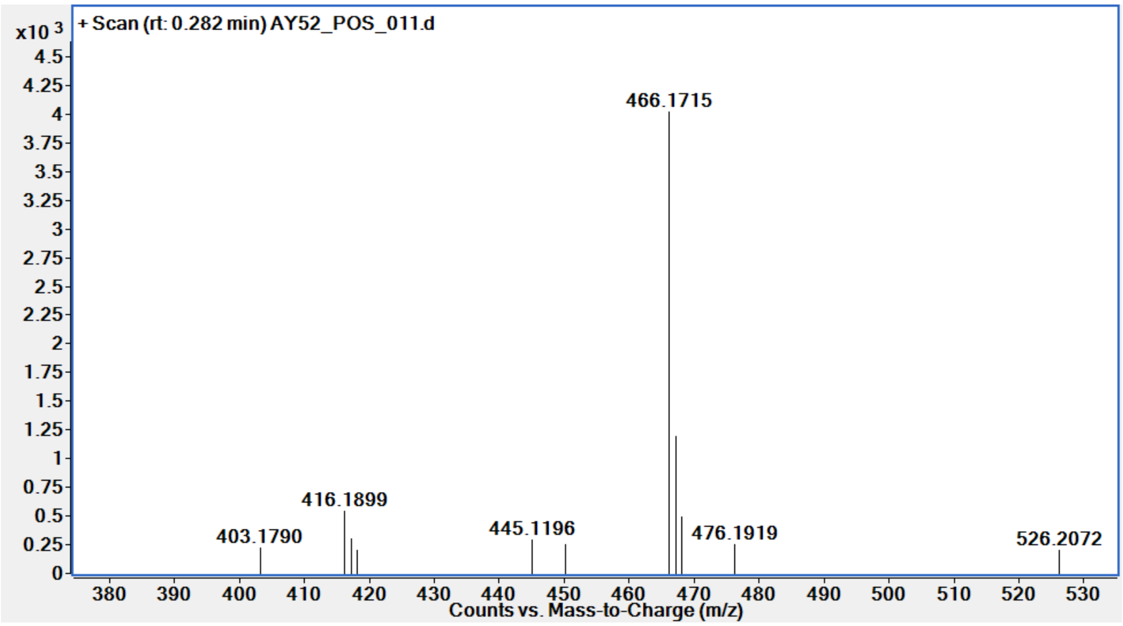
x10 3
+ Scan (rt: 0.282 min) AY52_POS_011.d
466.1715
416.1899
403.1790
445.1196
476.1919
526.2072
Counts vs. Mass-to-Charge (m/z)

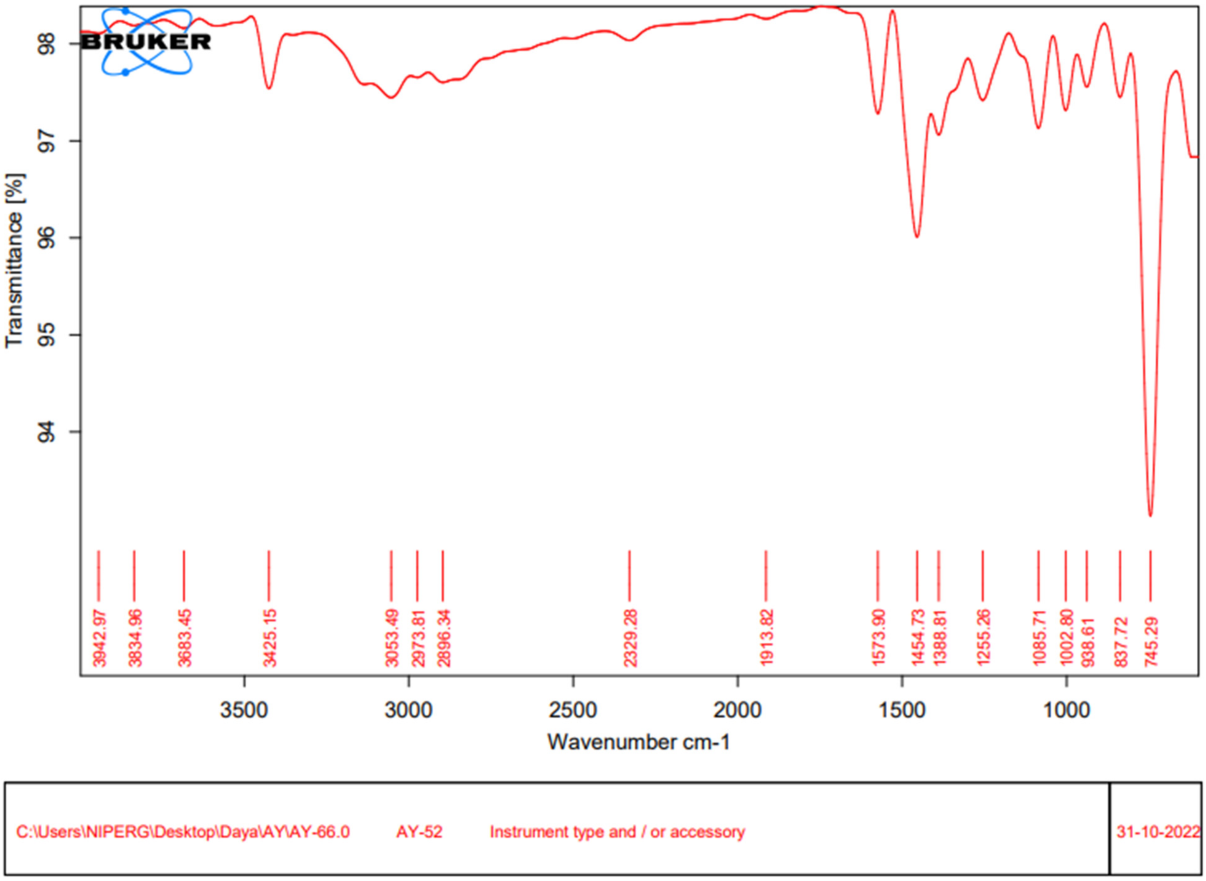
BRUKER
Transmittance [%]
Wavenumber cm-1
3942.97
3834.96
3683.45
3425.15
3053.49
2973.81
2896.34
2329.28
1913.82
1573.90
1454.73
1388.81
1255.26
1085.71
1002.80
938.61
837.72
745.29
C:\Users\NIPERG\Desktop\Daya\AY\AY-66.0
AY-52
Instrument type and / or accessory
31-10-2022

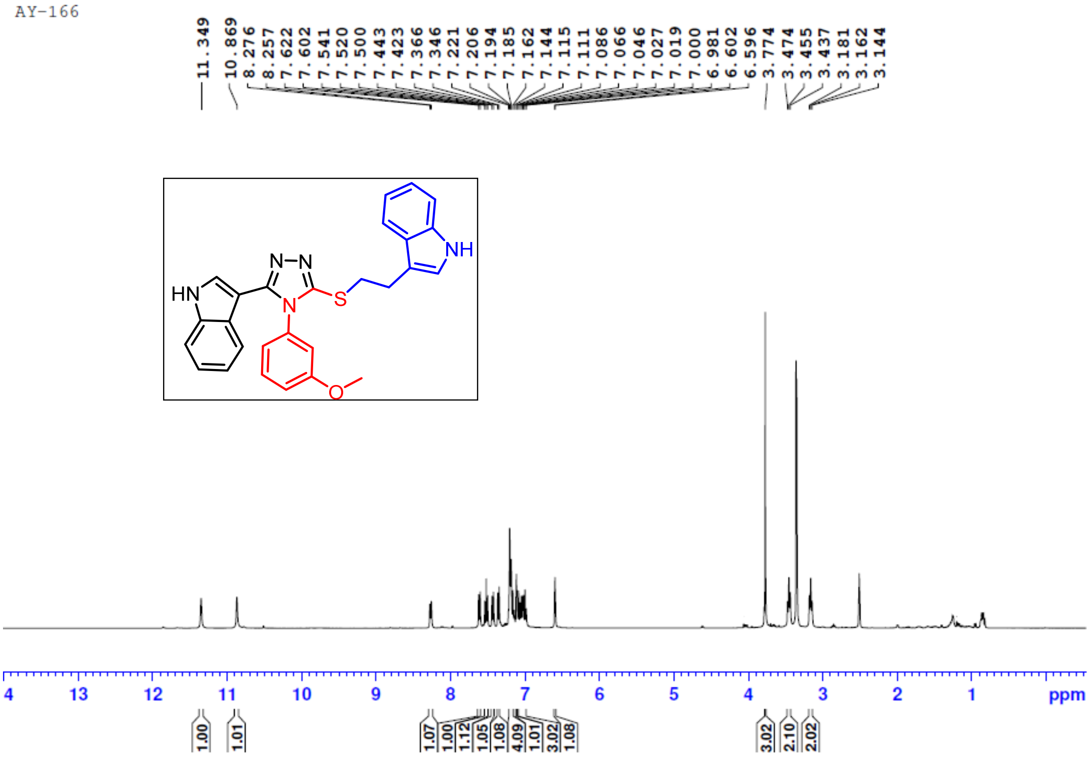
AY-166
11.349
10.869
8.276
8.257
7.622
7.602
7.541
7.520
7.500
7.443
7.423
7.366
7.346
7.221
7.206
7.194
7.185
7.162
7.144
7.115
7.111
7.086
7.066
7.046
7.027
7.019
7.000
6.981
6.602
6.596
3.774
3.474
3.455
3.437
3.181
3.162
3.144
HN
N–N
N
S
NH
O
ppm
1.00
1.01
1.07
1.00
1.12
1.05
1.08
4.09
1.01
3.02
1.08
3.02
2.10
2.02

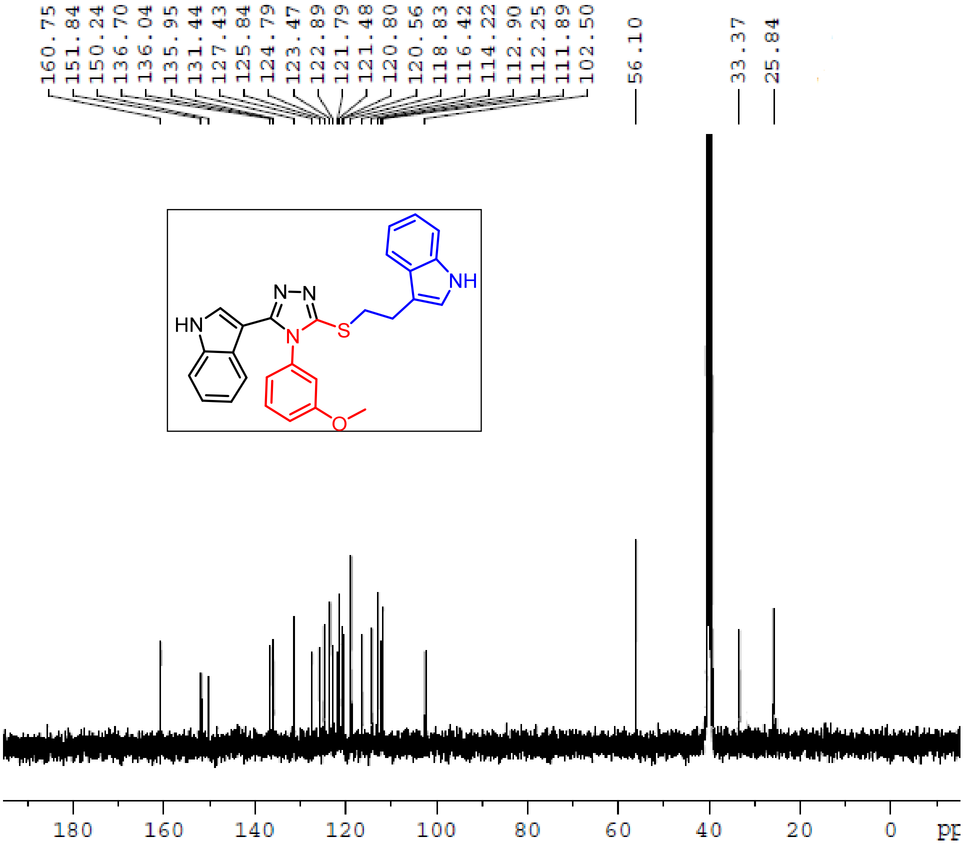
160.75
151.84
150.24
136.70
136.04
135.95
131.44
127.43
125.84
124.79
123.47
122.89
121.79
121.48
120.80
120.56
118.83
116.42
114.22
112.90
112.25
111.89
102.50
56.10
33.37
25.84
HN
N–N
N
S
NH
O

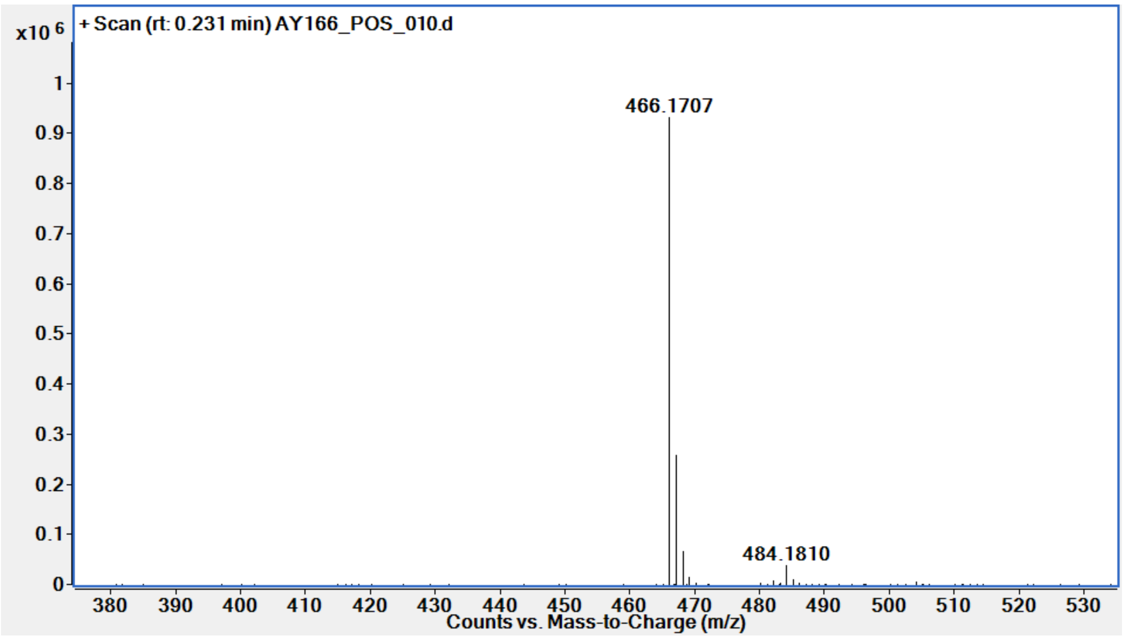

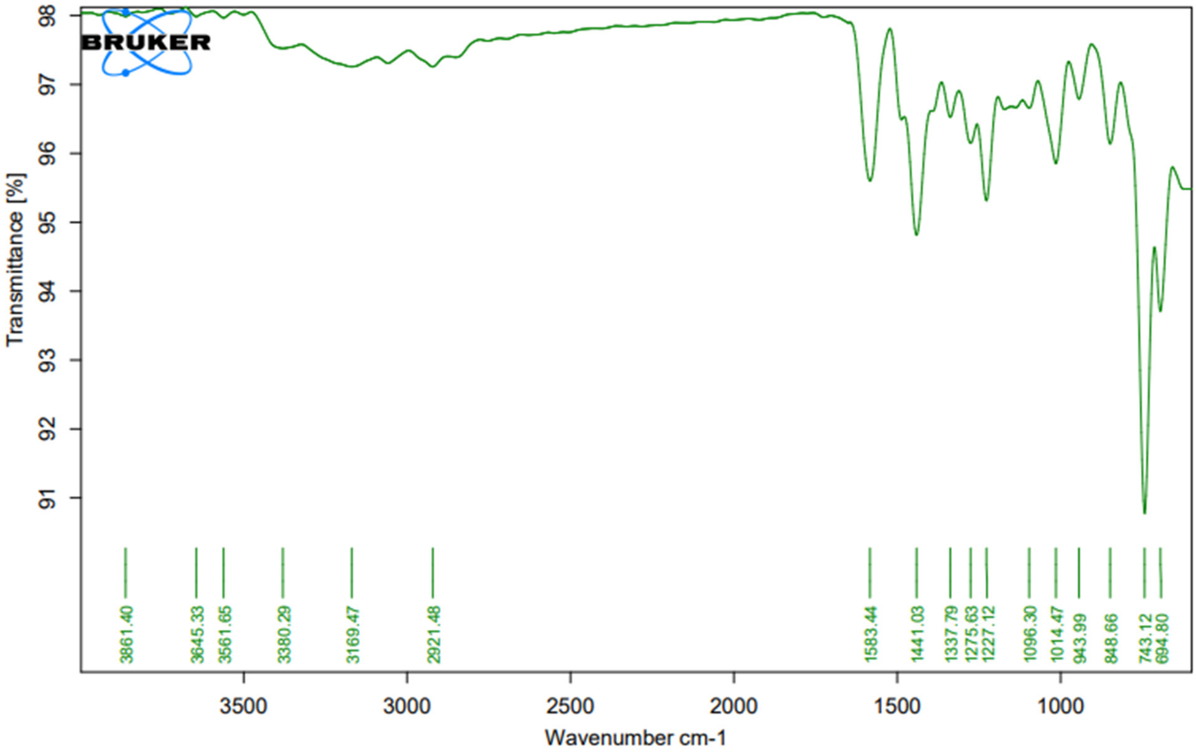

C:\Users\NIPERG\Desktop\Daya\AY\AY-166.0	AY-166	Instrument type and / or accessory	01-11-2022

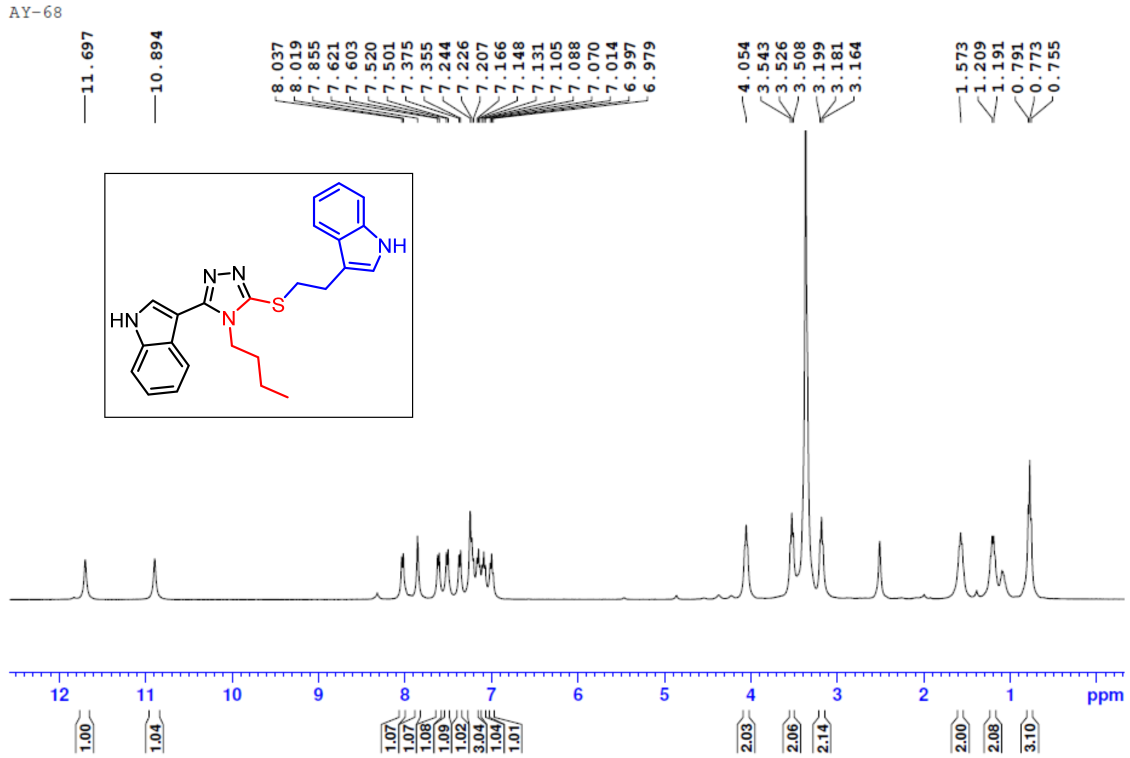

AY-68
11.697
10.894
8.037
8.019
7.855
7.621
7.603
7.520
7.501
7.375
7.355
7.244
7.226
7.207
7.166
7.148
7.131
7.105
7.088
7.070
7.014
6.997
6.979
4.054
3.543
3.526
3.508
3.199
3.181
3.164
1.573
1.209
1.191
0.791
0.773
0.755
HN
N
N
N
S
NH
12
11
10
9
8
7
6
5
4
3
2
1
ppm
1.00
1.04
1.07
1.07
1.08
1.09
1.02
3.04
1.04
1.01
2.03
2.06
2.14
2.00
2.08
3.10

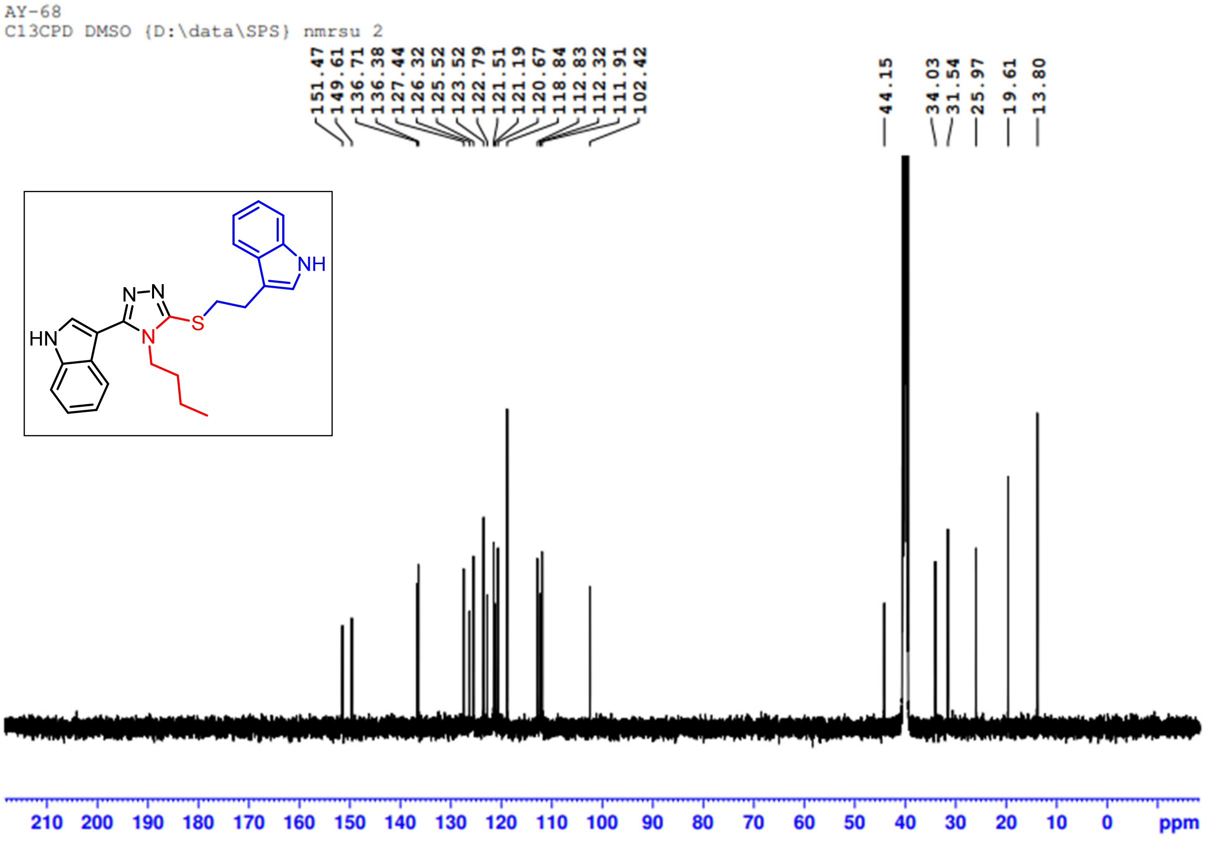

AY-68
C13CPD DMSO {D:\data\SPS} nmrsu 2
151.47
149.61
136.71
136.38
127.44
126.32
125.52
123.52
122.79
121.51
121.19
120.67
118.84
112.83
112.32
111.91
102.42
44.15
34.03
31.54
25.97
19.61
13.80
HN
N
N
N
S
NH
210
200
190
180
170
160
150
140
130
120
110
100
90
80
70
60
50
40
30
20
10
0
ppm

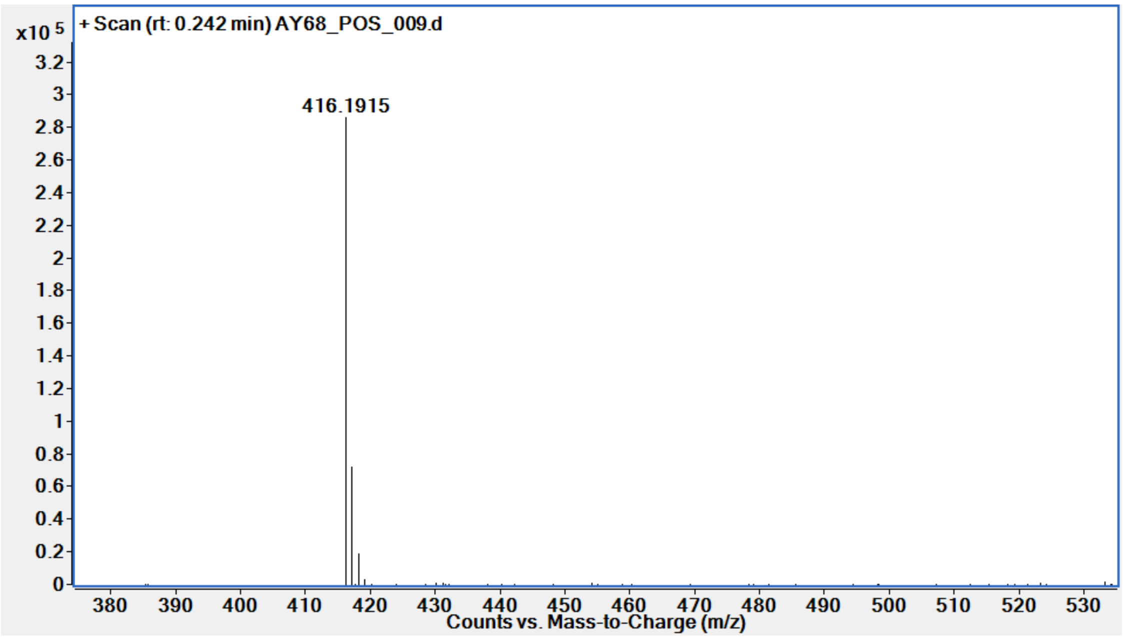
x10 5
+ Scan (rt: 0.242 min) AY68_POS_009.d
416.1915
Counts vs. Mass-to-Charge (m/z)

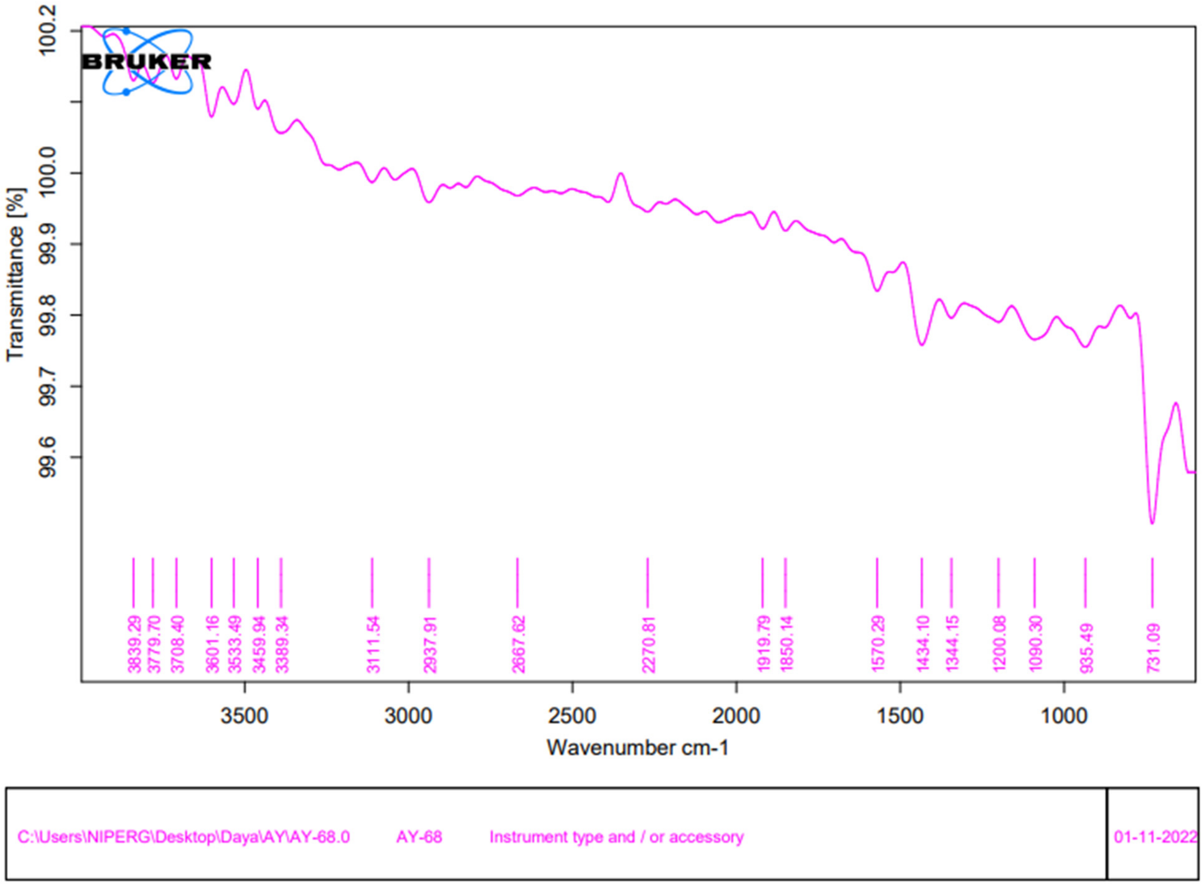
BRUKER
Transmittance [%]
Wavenumber cm-1
3839.29
3779.70
3708.40
3601.16
3533.49
3459.94
3389.34
3111.54
2937.91
2667.62
2270.81
1919.79
1850.14
1570.29
1434.10
1344.15
1200.08
1090.30
935.49
731.09
C:\Users\NIPERG\Desktop\Daya\AY\AY-68.0
AY-68
Instrument type and / or accessory
01-11-2022

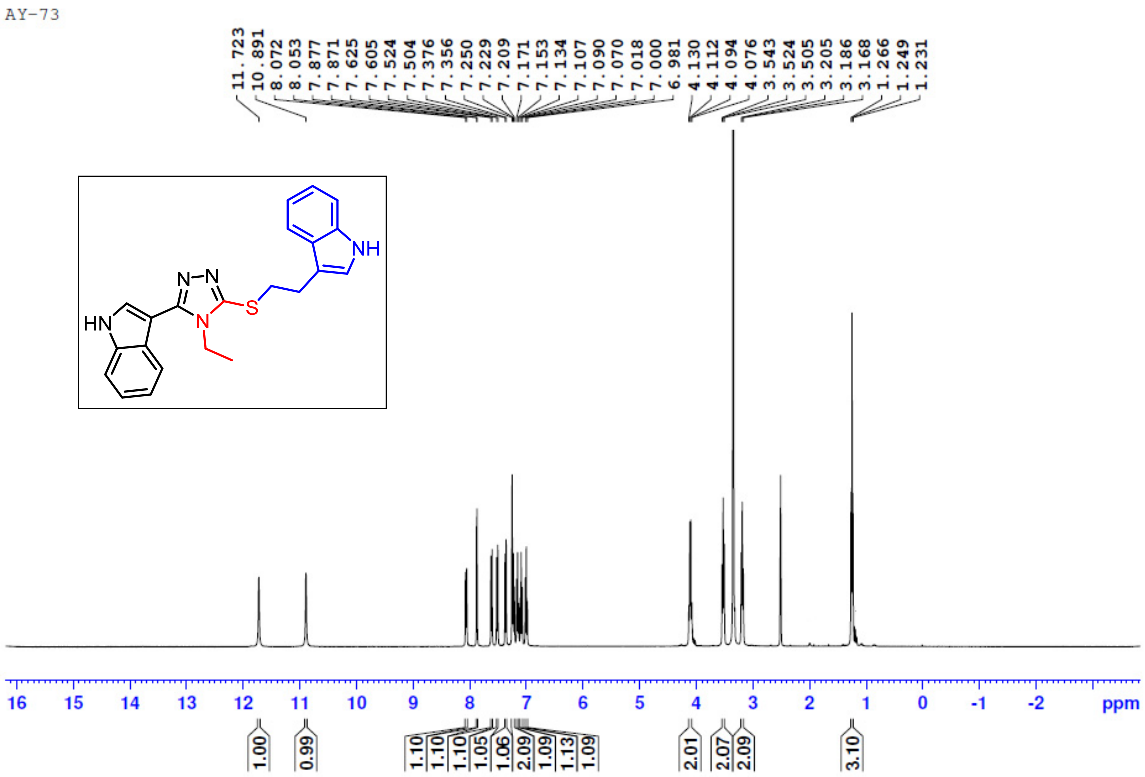
AY-73
11.723
10.891
8.072
8.053
7.877
7.871
7.625
7.605
7.524
7.504
7.376
7.356
7.250
7.229
7.209
7.171
7.153
7.134
7.107
7.090
7.070
7.018
7.000
6.981
4.130
4.112
4.094
4.076
3.543
3.524
3.505
3.205
3.186
3.168
1.266
1.249
1.231
NH
HN
N–N
S
N
ppm
1.00
0.99
1.10
1.10
1.10
1.05
1.06
2.09
1.09
1.13
1.09
2.01
2.07
2.09
3.10

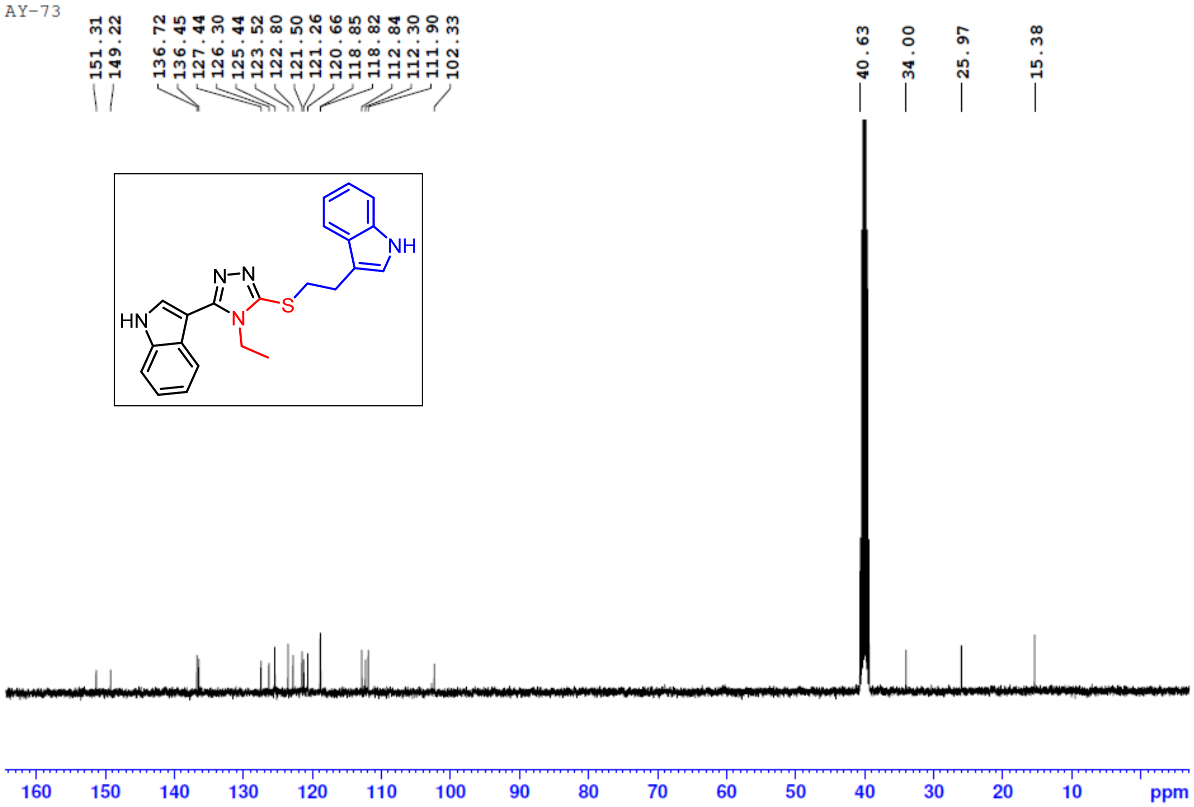
AY-73
151.31
149.22
136.72
136.45
127.44
126.30
125.44
123.52
122.80
121.50
121.26
120.66
118.85
118.82
112.84
112.30
111.90
102.33
40.63
34.00
25.97
15.38
NH
HN
N–N
S
N
ppm

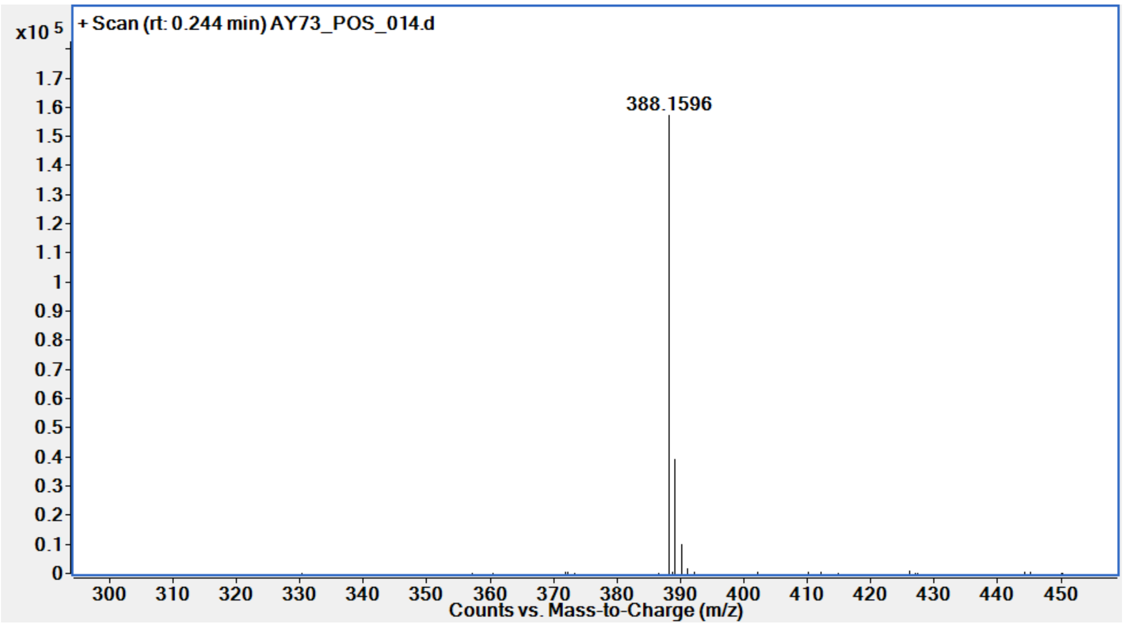
x10 5
+ Scan (rt: 0.244 min) AY73_POS_014.d
388.1596
Counts vs. Mass-to-Charge (m/z)

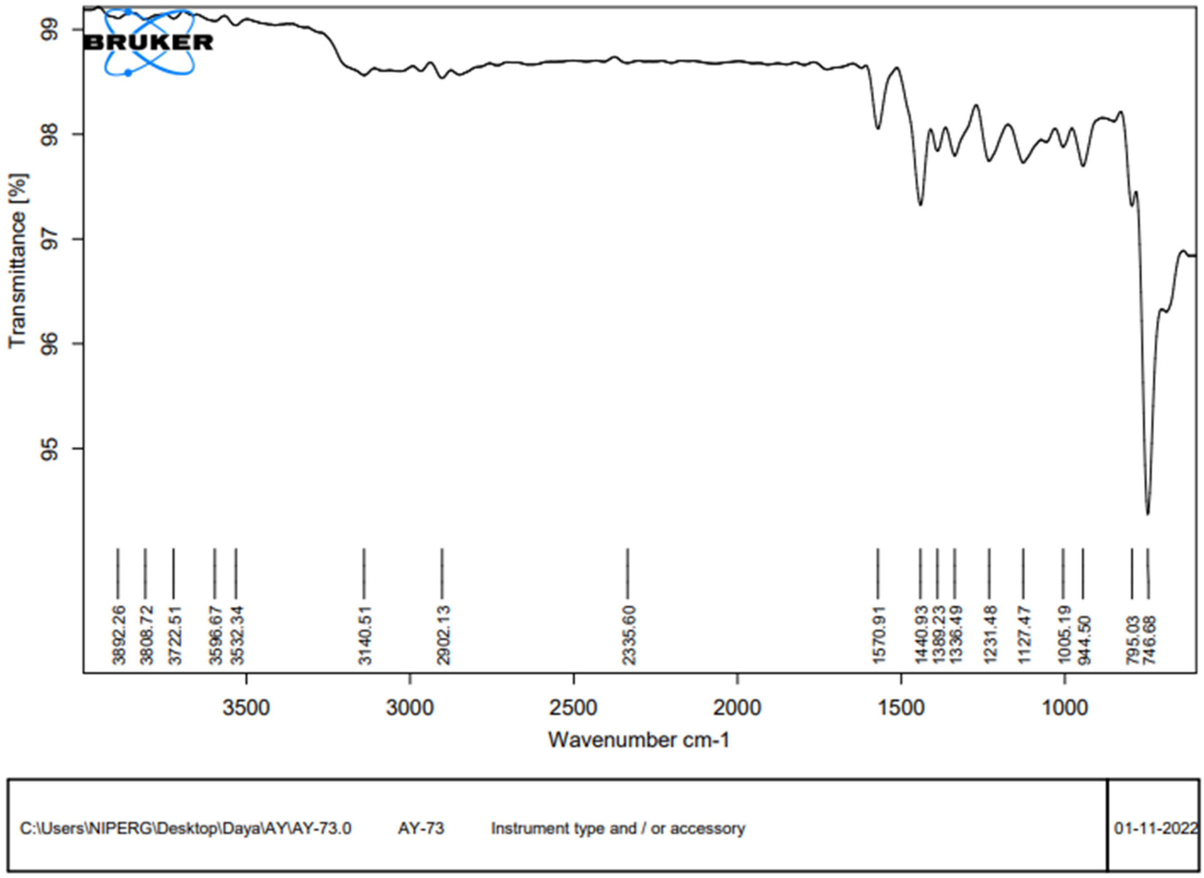
BRUKER
Transmittance [%]
Wavenumber cm-1
3892.26
3808.72
3722.51
3596.67
3532.34
3140.51
2902.13
2335.60
1570.91
1440.93
1389.23
1336.49
1231.48
1127.47
1005.19
944.50
795.03
746.68
C:\Users\NIPERG\Desktop\Daya\AY\AY-73.0
AY-73
Instrument type and / or accessory
01-11-2022

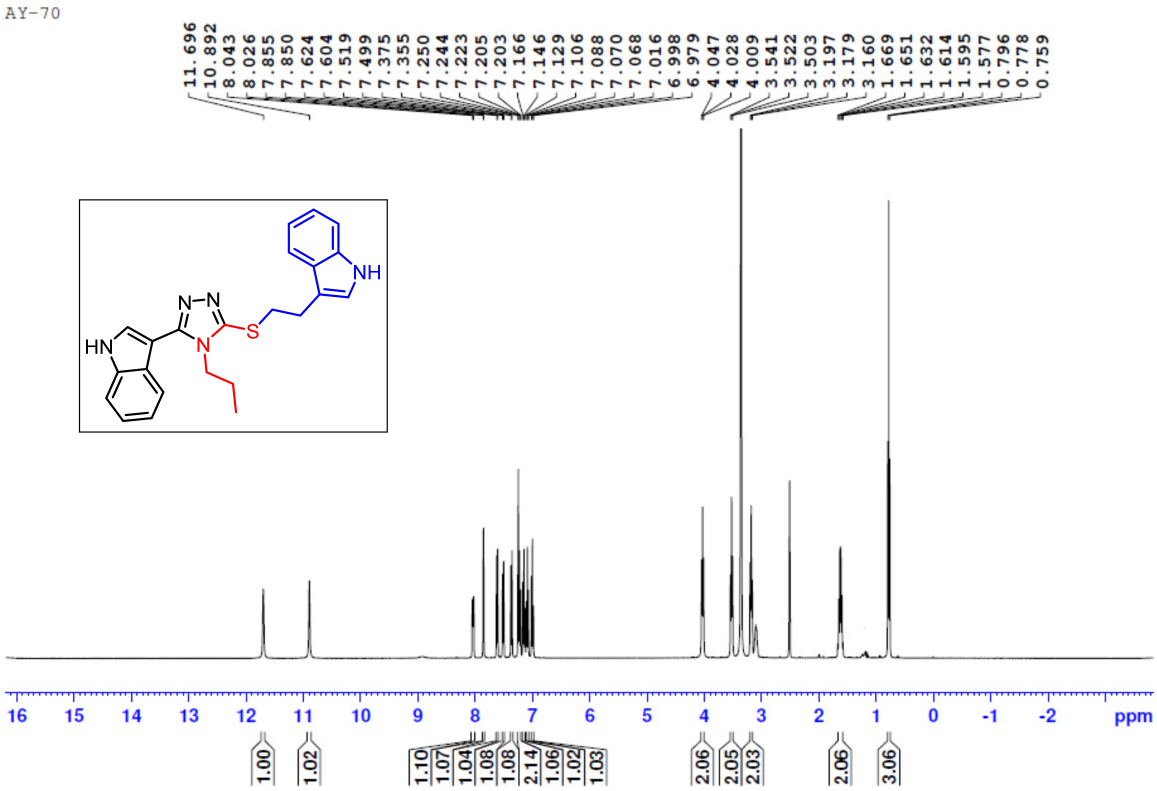
AY-70
11.696
10.892
8.043
8.026
7.855
7.850
7.624
7.604
7.519
7.499
7.375
7.355
7.250
7.244
7.223
7.205
7.203
7.166
7.146
7.129
7.106
7.088
7.070
7.068
7.016
6.998
6.979
4.047
4.028
4.009
3.541
3.522
3.503
3.197
3.179
3.160
1.669
1.651
1.632
1.614
1.595
1.577
0.796
0.778
0.759
N–N
NH
HN
N
S
16 15 14 13 12 11 10 9 8 7 6 5 4 3 2 1 0 -1 -2 ppm
1.00
1.02
1.10
1.07
1.04
1.08
1.08
2.14
1.06
1.02
1.03
2.06
2.05
2.03
2.06
3.06

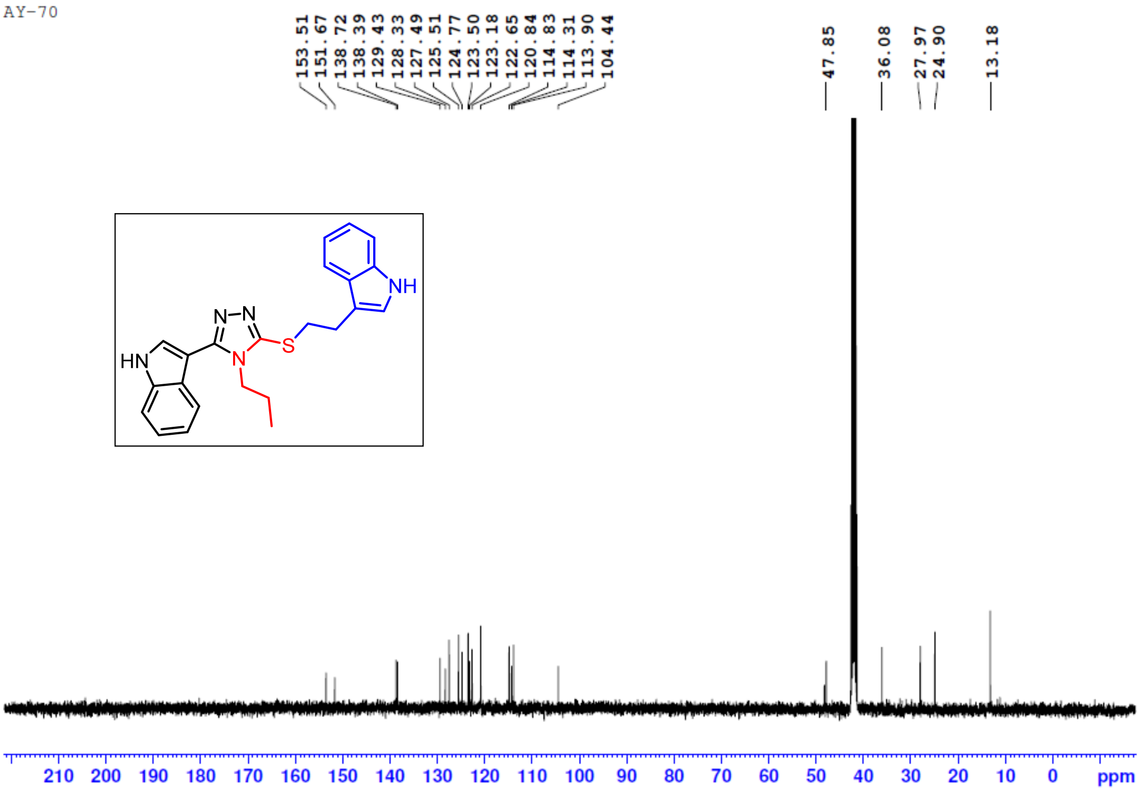
AY-70
153.51
151.67
138.72
138.39
129.43
128.33
127.49
125.51
124.77
123.50
123.18
122.65
120.84
114.83
114.31
113.90
104.44
47.85
36.08
27.97
24.90
13.18
N–N
NH
HN
N
S
210 200 190 180 170 160 150 140 130 120 110 100 90 80 70 60 50 40 30 20 10 0 ppm

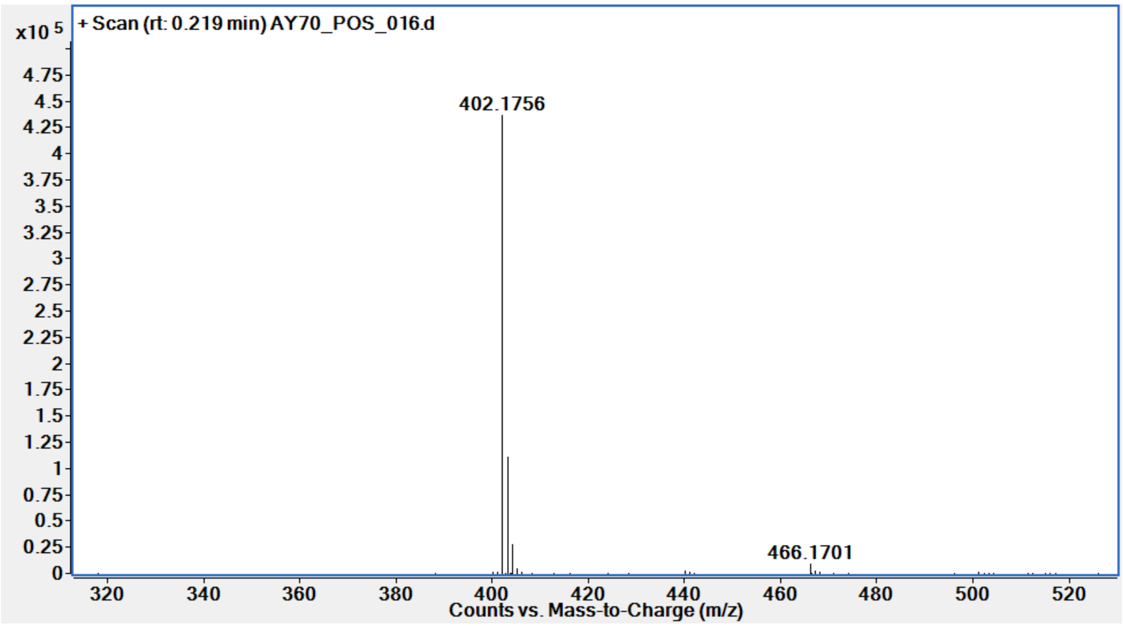
x10 5
+ Scan (rt: 0.219 min) AY70_POS_016.d
402.1756
466.1701
Counts vs. Mass-to-Charge (m/z)

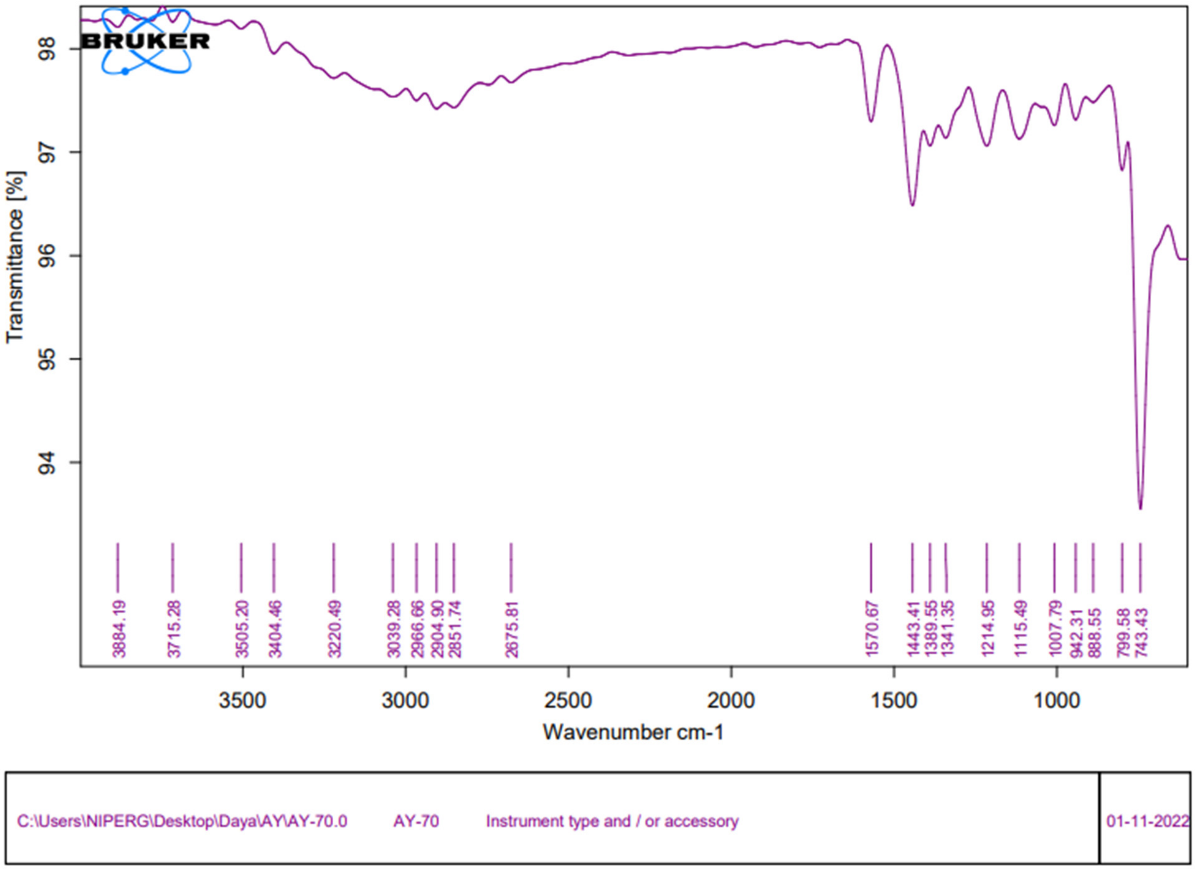
BRUKER
Transmittance [%]
Wavenumber cm-1
3884.19
3715.28
3505.20
3404.46
3220.49
3039.28
2966.66
2904.90
2851.74
2675.81
1570.67
1443.41
1389.55
1341.35
1214.95
1115.49
1007.79
942.31
888.55
799.58
743.43
C:\Users\NIPERG\Desktop\Daya\AY\AY-70.0
AY-70
Instrument type and / or accessory
01-11-2022

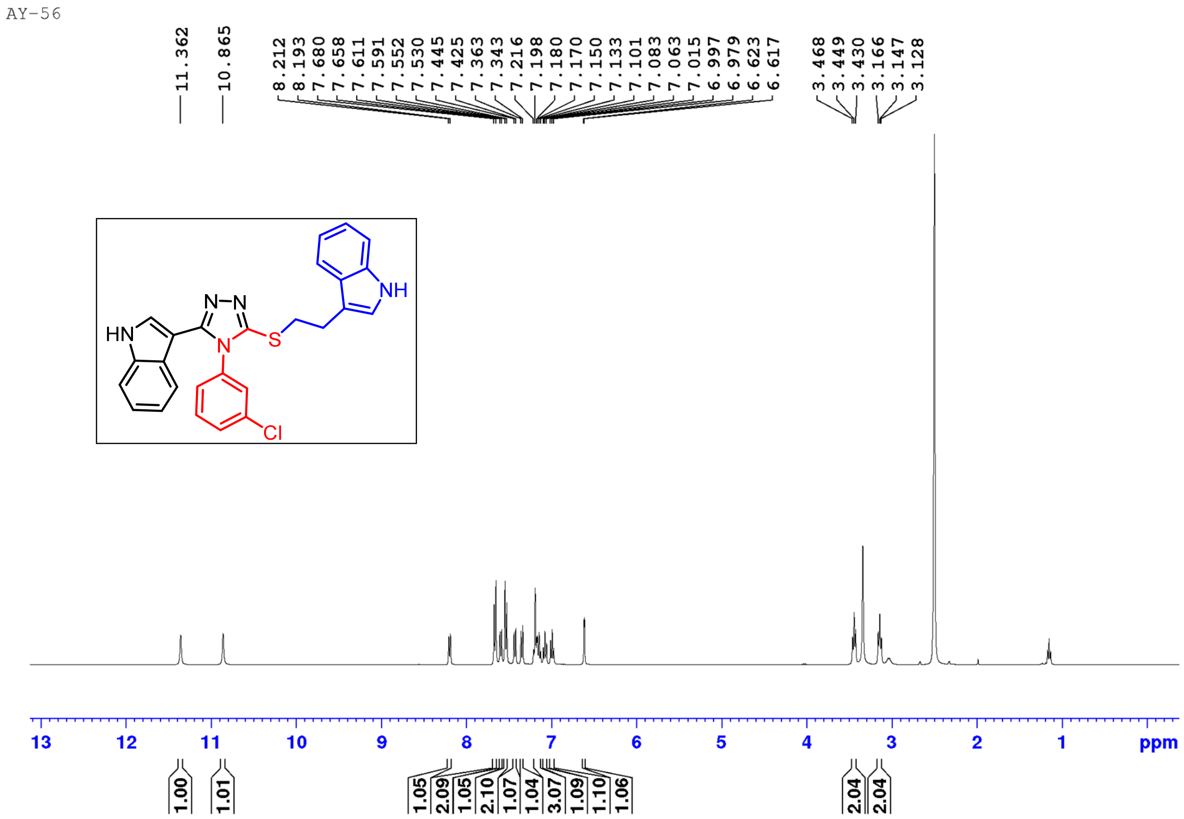
AY-56
11.362
10.865
8.212
8.193
7.680
7.658
7.611
7.591
7.552
7.530
7.445
7.425
7.363
7.343
7.216
7.198
7.180
7.170
7.150
7.133
7.101
7.083
7.063
7.015
6.997
6.979
6.623
6.617
3.468
3.449
3.430
3.166
3.147
3.128
HN
N–N
N
S
NH
Cl
13 12 11 10 9 8 7 6 5 4 3 2 1 ppm
1.00
1.01
1.05
2.09
1.05
2.10
1.07
1.04
3.07
1.09
1.10
1.06
2.04
2.04

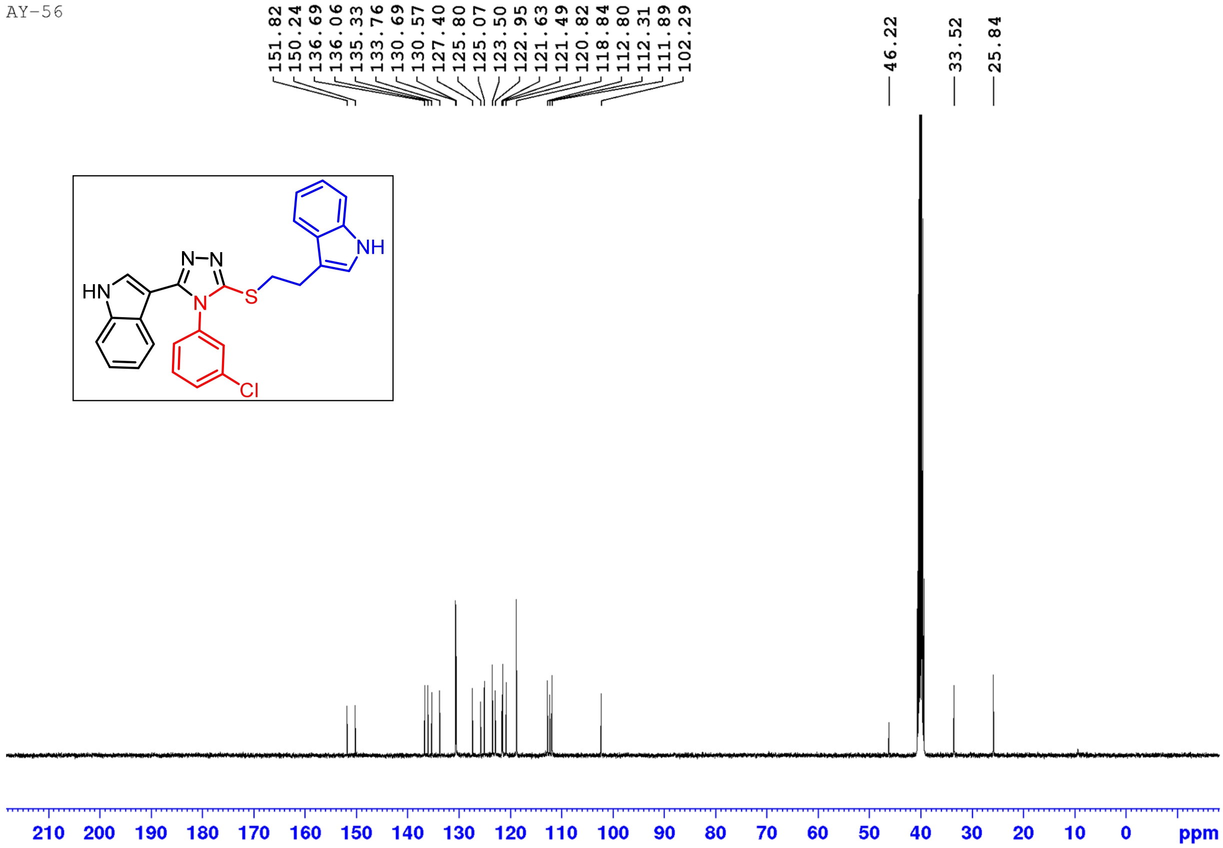
AY-56
151.82
150.24
136.69
136.06
135.33
133.76
130.69
130.57
127.40
125.80
125.07
123.50
122.95
121.63
121.49
120.82
118.84
112.80
112.31
111.89
102.29
46.22
33.52
25.84
HN
N–N
N
S
NH
Cl
210 200 190 180 170 160 150 140 130 120 110 100 90 80 70 60 50 40 30 20 10 0 ppm

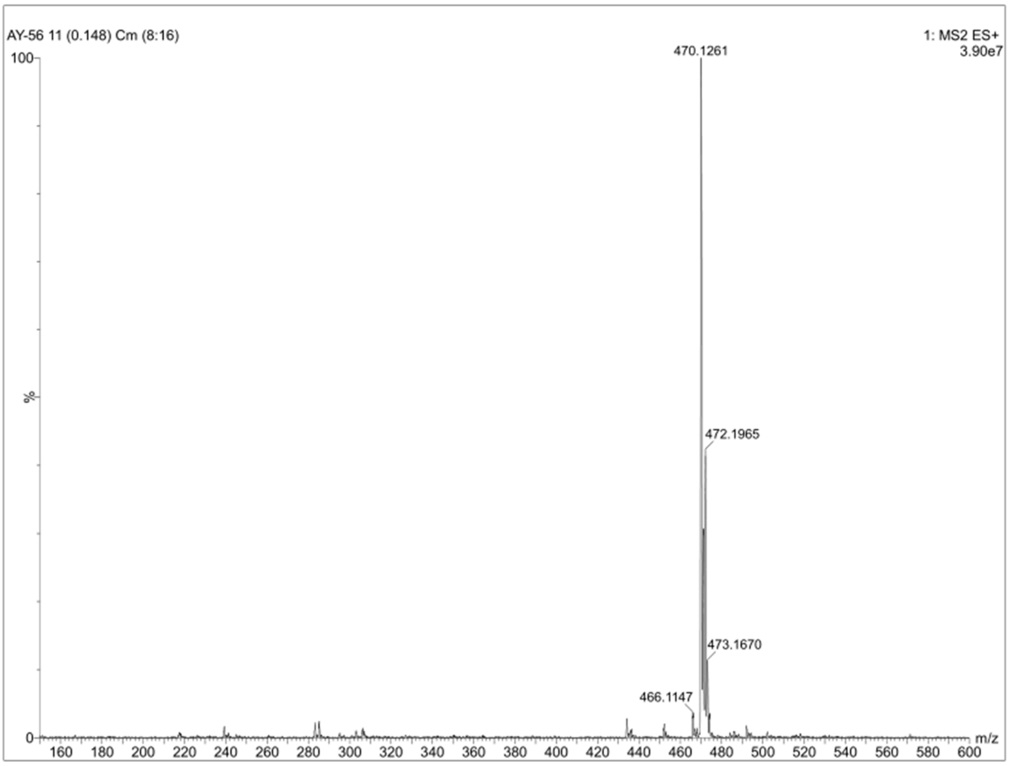
AY-56 11 (0.148) Cm (8:16)
1: MS2 ES+
3.90e7
470.1261
472.1965
473.1670
466.1147
100
%
0
m/z
160 180 200 220 240 260 280 300 320 340 360 380 400 420 440 460 480 500 520 540 560 580 600

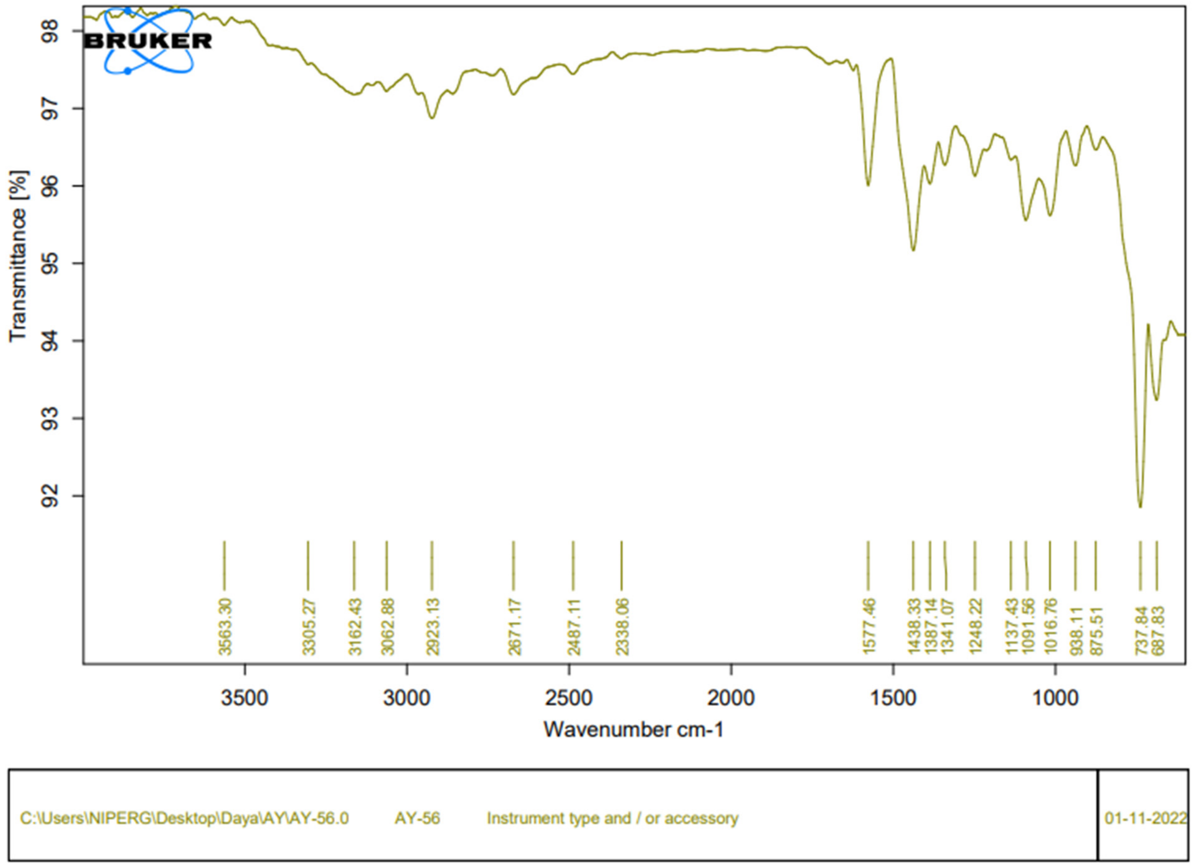
BRUKER
Transmittance [%]
98
97
96
95
94
93
92
3563.30
3305.27
3162.43
3062.88
2923.13
2671.17
2487.11
2338.06
1577.46
1438.33
1387.14
1341.07
1248.22
1137.43
1091.56
1016.76
938.11
875.51
737.84
687.83
3500
3000
2500
2000
1500
1000
Wavenumber cm-1
C:\Users\NIPERG\Desktop\Daya\AY\AY-56.0
AY-56
Instrument type and / or accessory
01-11-2022

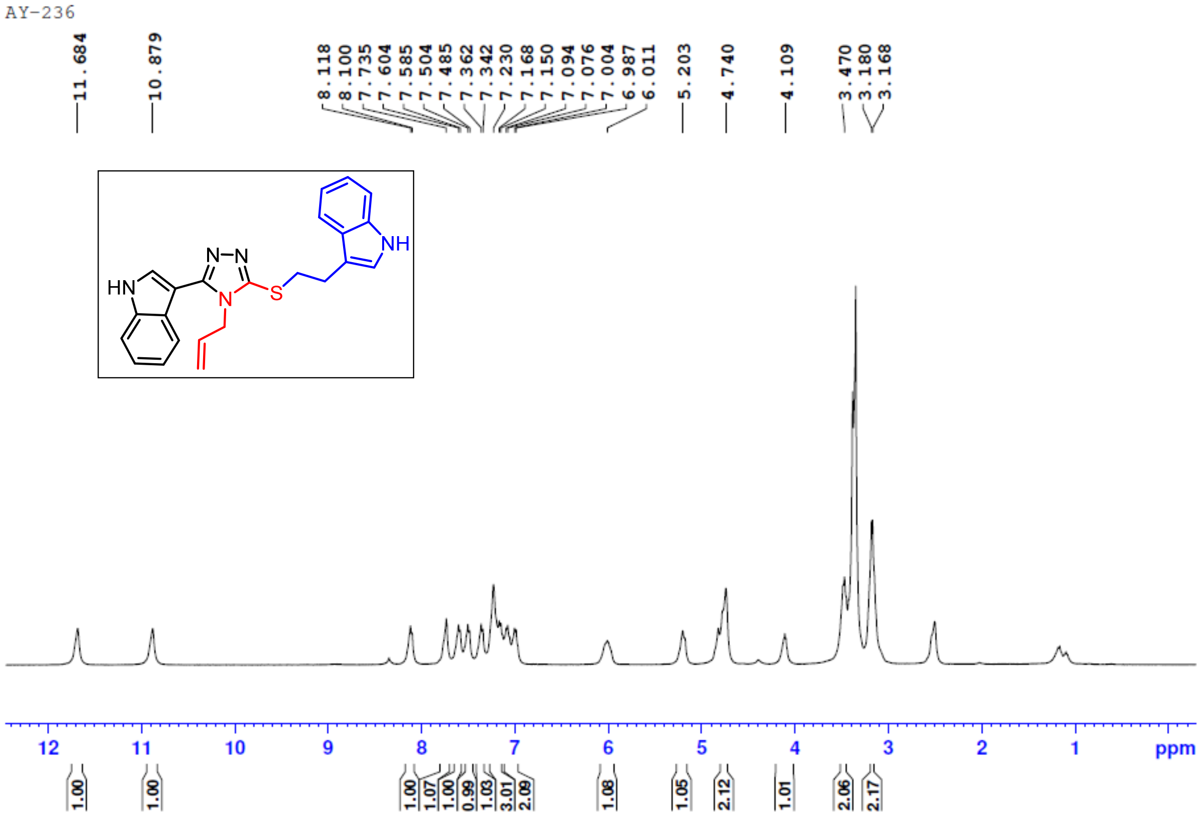
AY-236
11.684
10.879
8.118
8.100
7.735
7.604
7.585
7.504
7.485
7.362
7.342
7.230
7.168
7.150
7.094
7.076
7.004
6.987
6.011
5.203
4.740
4.109
3.470
3.180
3.168
HN
N–N
N
S
NH
12
11
10
9
8
7
6
5
4
3
2
1
ppm
1.00
1.00
1.00
1.07
1.00
0.99
1.03
3.01
2.09
1.08
1.05
2.12
1.01
2.06
2.17

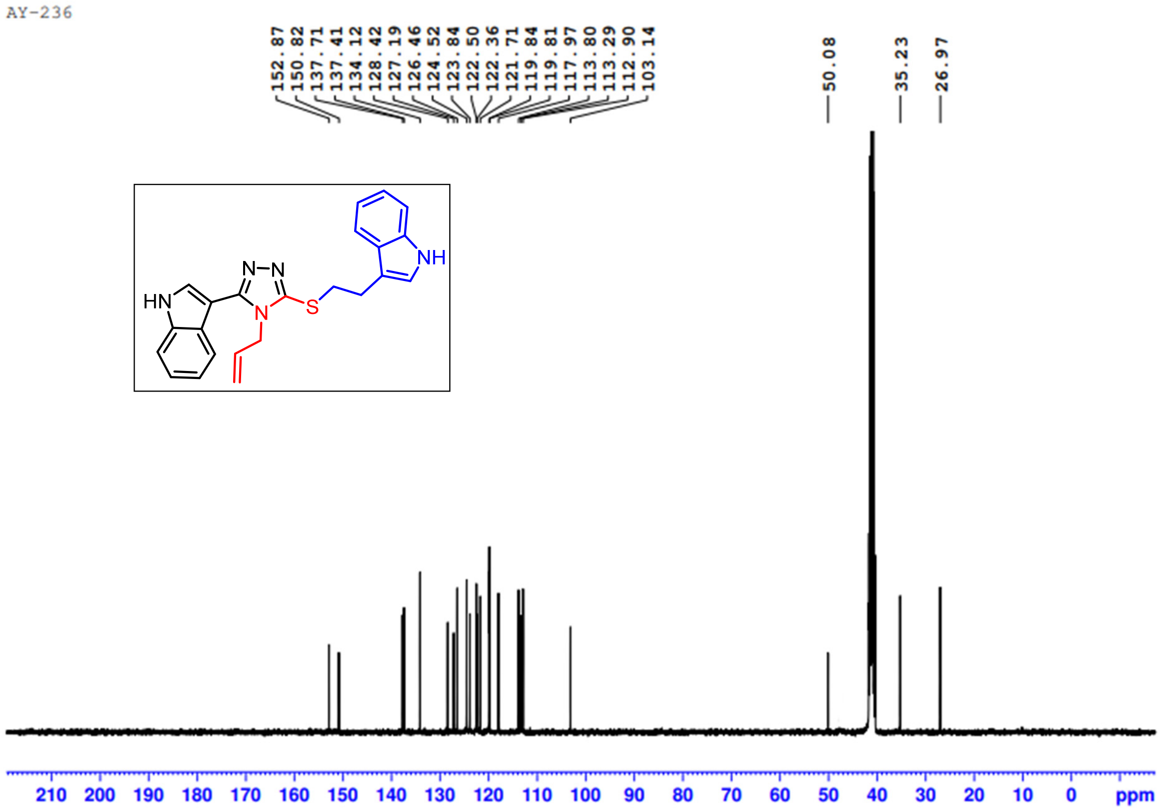
AY-236
152.87
150.82
137.71
137.41
134.12
128.42
127.19
126.46
124.52
123.84
122.50
122.36
121.71
119.84
119.81
117.97
113.80
113.29
112.90
103.14
50.08
35.23
26.97
HN
N–N
N
S
NH
210
200
190
180
170
160
150
140
130
120
110
100
90
80
70
60
50
40
30
20
10
0
ppm

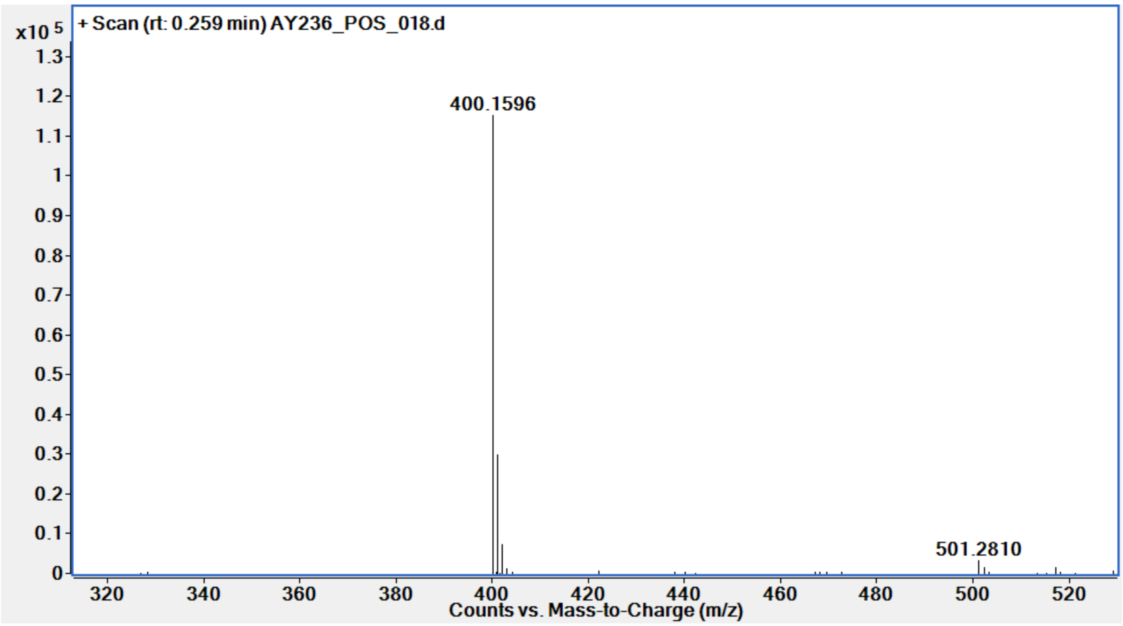

x10 5
+ Scan (rt: 0.259 min) AY236_POS_018.d
400.1596
501.2810
Counts vs. Mass-to-Charge (m/z)

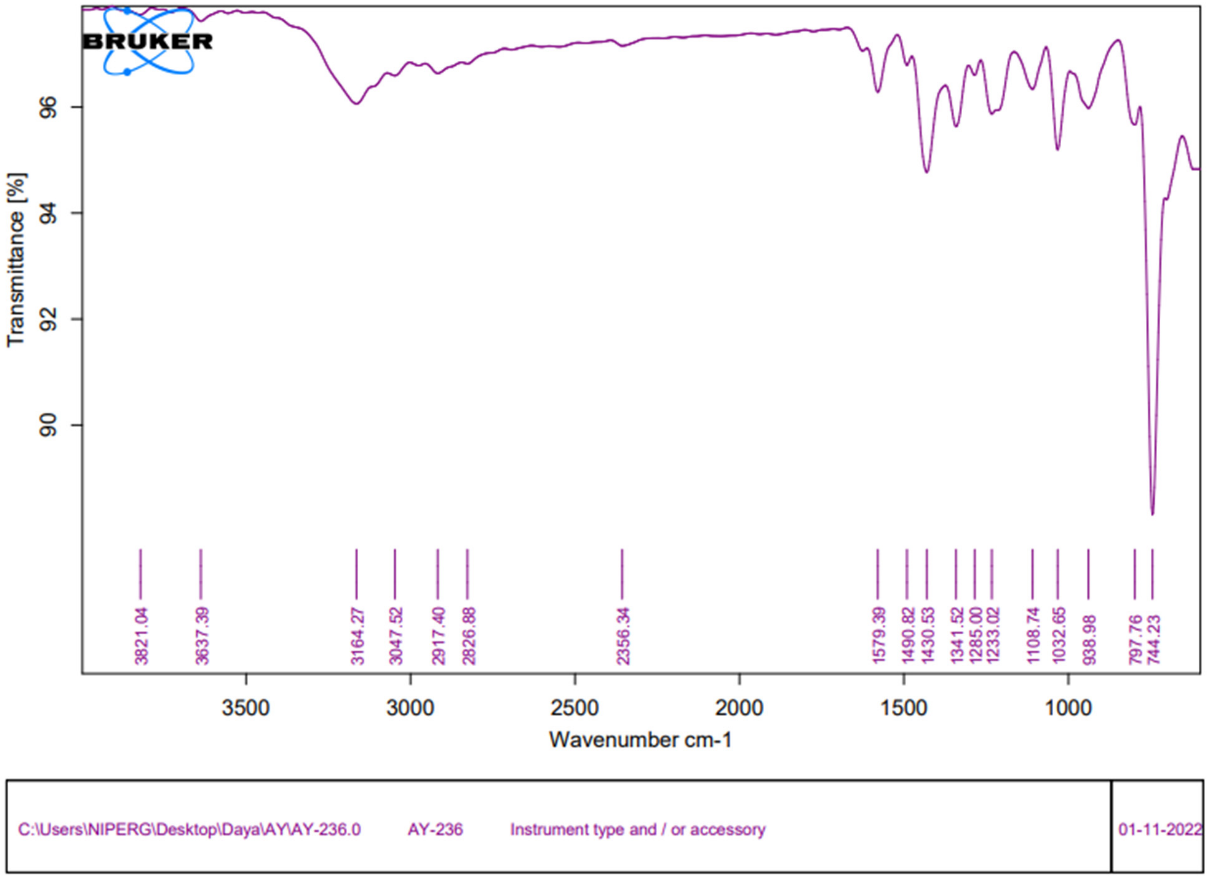

BRUKER
Transmittance [%]
Wavenumber cm-1
3821.04
3637.39
3164.27
3047.52
2917.40
2826.88
2356.34
1579.39
1490.82
1430.53
1341.52
1285.00
1233.02
1108.74
1032.65
938.98
797.76
744.23
C:\Users\NIPERG\Desktop\Daya\AY\AY-236.0
AY-236
Instrument type and / or accessory
01-11-2022

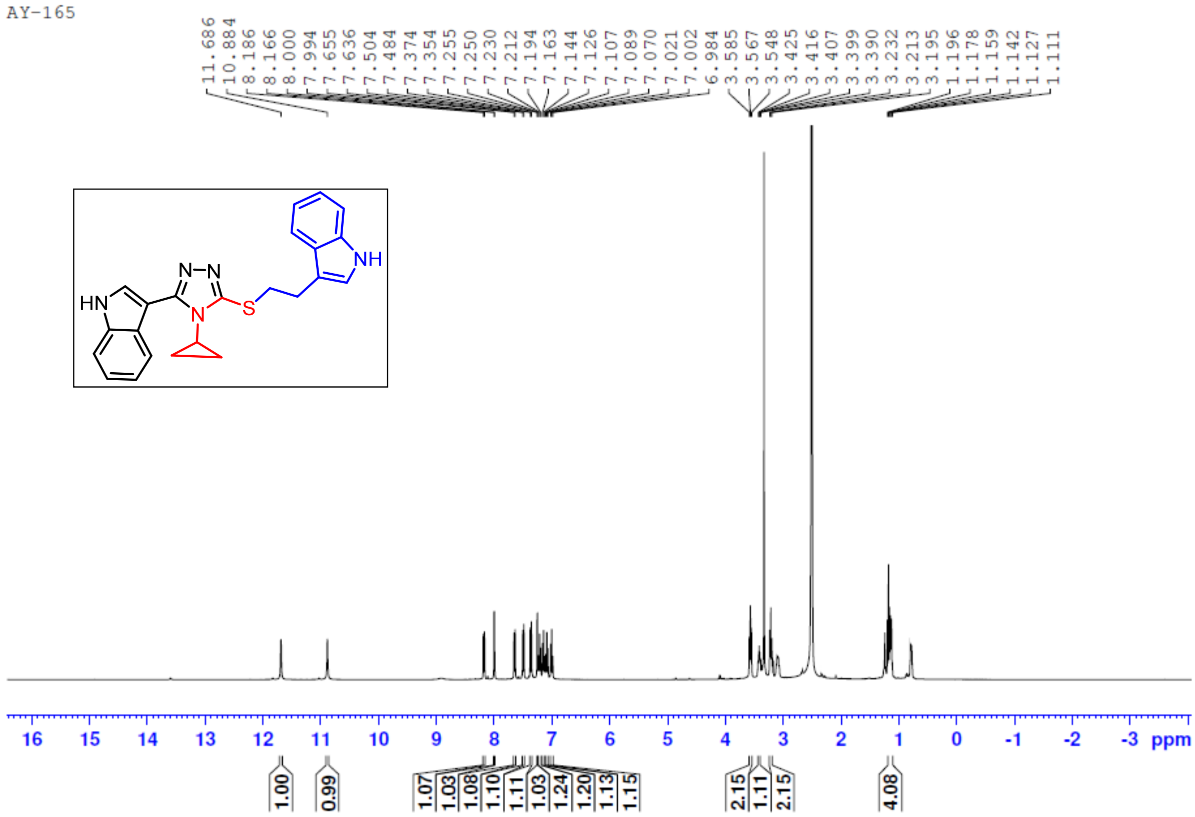
AY-165
11.686
10.884
8.186
8.166
8.000
7.994
7.655
7.636
7.504
7.484
7.374
7.354
7.255
7.250
7.230
7.212
7.194
7.163
7.144
7.126
7.107
7.089
7.070
7.021
7.002
6.984
3.585
3.567
3.548
3.425
3.416
3.407
3.399
3.390
3.390
3.232
3.213
3.195
1.196
1.178
1.159
1.142
1.127
1.111
HN
N–N
N
S
NH
16 15 14 13 12 11 10 9 8 7 6 5 4 3 2 1 0 -1 -2 -3 ppm
1.00
0.99
1.07
1.03
1.08
1.10
1.11
1.03
1.24
1.20
1.13
1.15
2.15
1.11
2.15
4.08

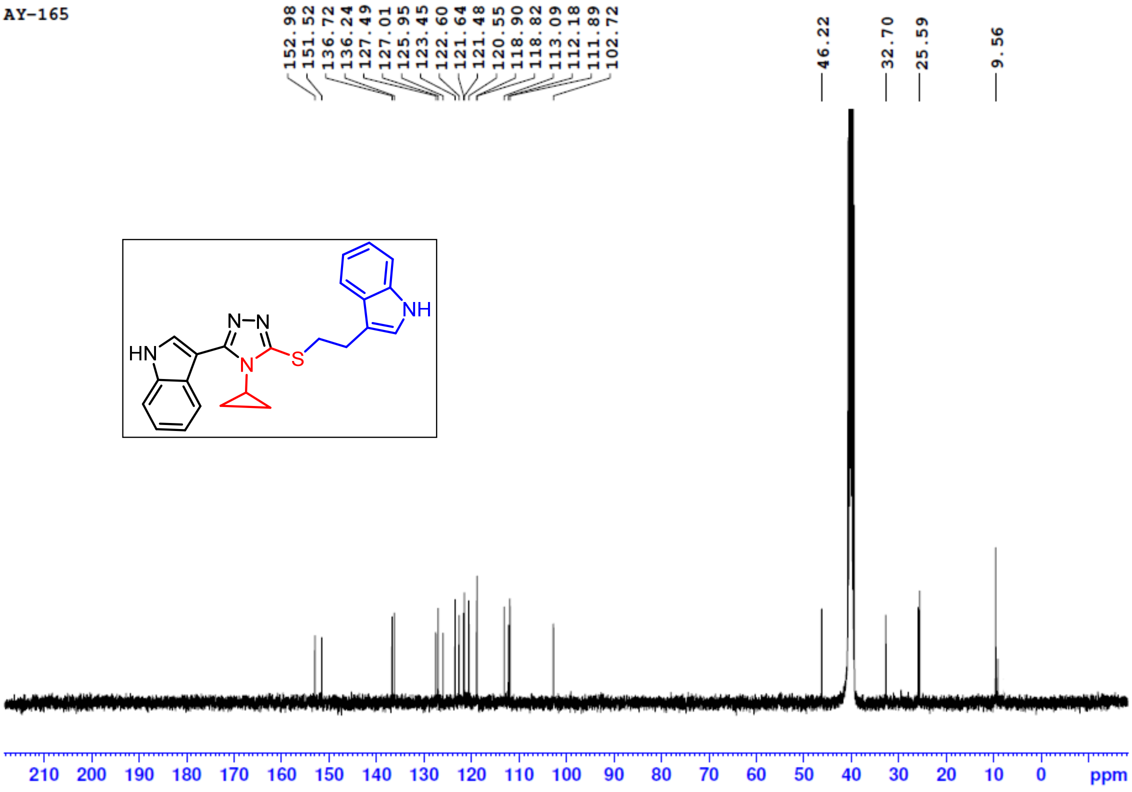
AY-165
152.98
151.52
136.72
136.24
127.49
127.01
125.95
123.45
122.60
121.64
121.48
120.55
118.90
118.82
113.09
112.18
111.89
102.72
46.22
32.70
25.59
9.56
HN
N–N
N
S
NH
210 200 190 180 170 160 150 140 130 120 110 100 90 80 70 60 50 40 30 20 10 0 ppm

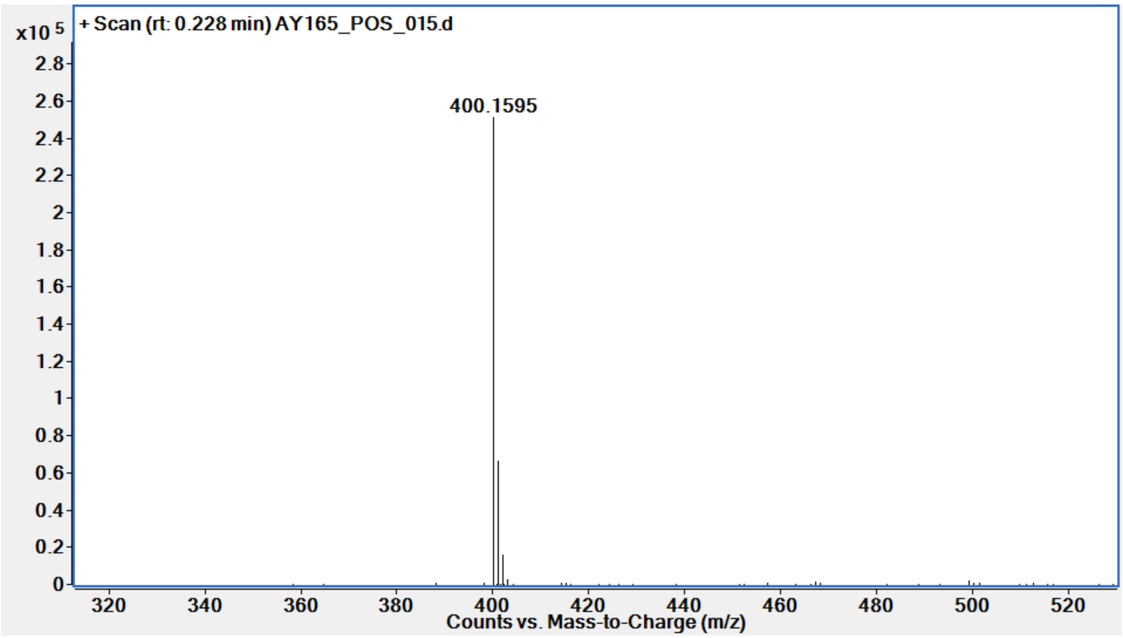

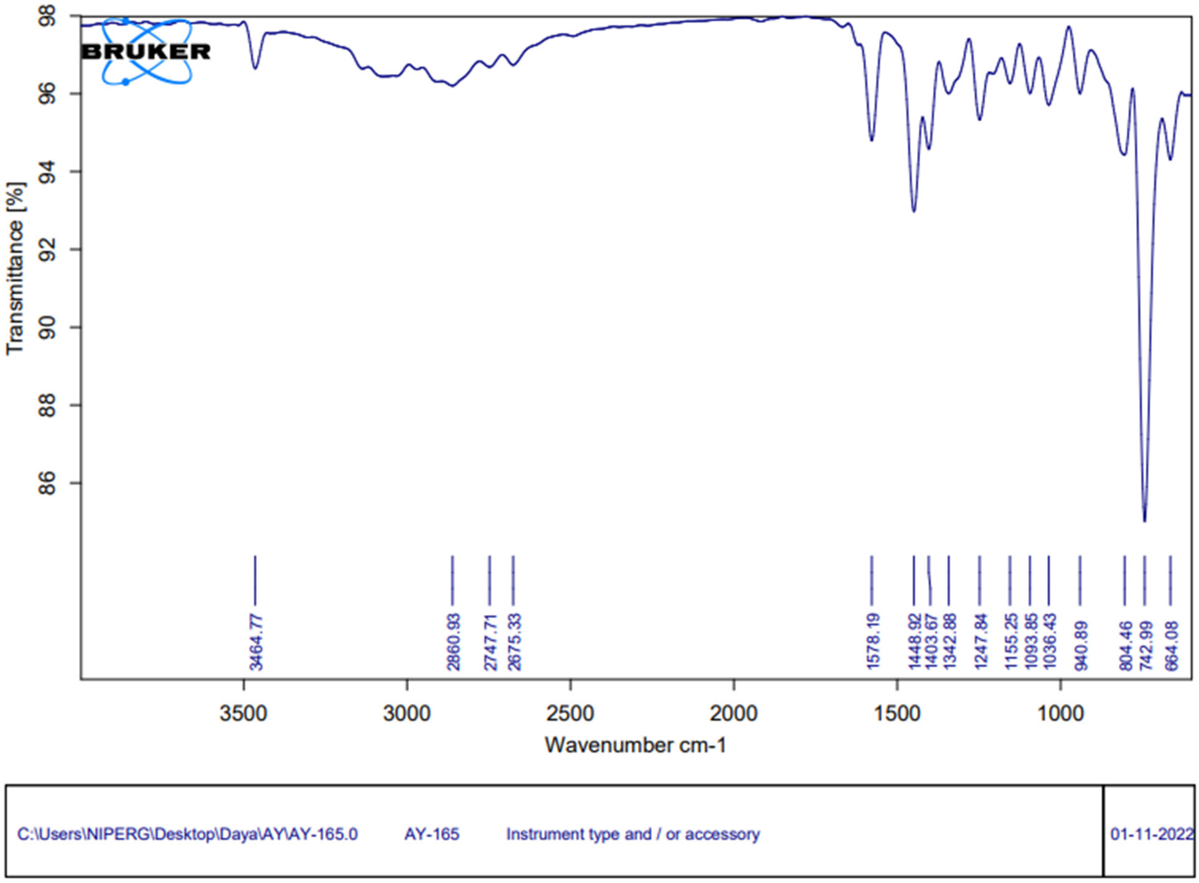

Figure S3: ^{1}H, ^{13}C NMR and Mass copies of all products.
